# Erector spinae plane block vs. paravertebral block for postoperative analgesia in breast surgery: a meta-analysis of randomized trials

**DOI:** 10.1016/j.bjane.2026.844767

**Published:** 2026-05-28

**Authors:** Burhan Dost, Yunus Emre Karapinar, Muzeyyen Beldagli, Esra Turunc, Cengiz Kaya, Alessandro De Cassai

**Affiliations:** a Ondokuz Mayis University Faculty of Medicine, Department of Anesthesiology and Reanimation, Samsun, Turkey; b Istanbul University-Cerrahpasa, Cerrahpasa Faculty of Medicine, Department of Anesthesiology and Reanimation, Istanbul, Turkey; c Samsun Training and Research Hospital, Department of Anesthesiology and Reanimation, Samsun, Turkey; d University of Padua, Department of Medicine (DIMED), Padua, Italy; e University Hospital of Padua, Institute of Anesthesia and Intensive Care Unit, Padua, Italy

Dear Editor,

Effective analgesia after breast surgery is essential because acute postoperative pain may impair recovery and contribute to chronic postsurgical pain.^1^ Paravertebral Block (PVB) remains a recommended regional analgesic technique for breast surgery, but its technical complexity and potential complications have prompted interest in simpler alternatives such as the Erector Spinae Plane Block (ESPB).^2^ Although previous meta-analyses have suggested broadly comparable analgesic effects between ESPB and PVB, available evidence has been limited by small trial numbers, heterogeneous populations, and variable methodology.^3^^,^^4^ In light of the recently published Cochrane network meta-analysis showing no clinically meaningful difference between ESPB and PVB for 24-hour postoperative pain, we designed our analysis as a focused direct comparison of ESPB versus PVB in adult breast surgery.^5^ Our aim was not to establish equivalence, but to examine whether the available head-to-head randomized evidence demonstrates a clinically meaningful difference in 24-hour opioid consumption, incorporating Trial Sequential Analysis (TSA), exploratory meta-regression, and a prespecified Minimum Clinically Important Difference (MCID) to improve clinical interpretation.

We conducted a prospectively registered systematic review and meta-analysis (PROSPERO CRD420251118777) of randomized trials comparing ESPB with PVB in adult breast surgery. PubMed, Scopus, Embase, CENTRAL, and Web of Science were searched to 4 August 2025. The primary outcome was 24-hour opioid consumption, converted to intravenous Morphine Milligram Equivalents (MME). Random-effects meta-analysis was performed, with GRADE certainty assessment, sensitivity/subgroup analyses, meta-regression, and TSA using a prespecified MCID of 10 mg intravenous MME.^6^ The search strategy, PRISMA flow diagram, study characteristics, risk-of-bias assessment, forest and funnel plots, meta-regression, subgroup and sensitivity analyses, TSA, and certainty-of-evidence assessment are provided in the Supplementary Material for consultation.

27 RCTs published between 2019 and 2025 were included, comprising 2015 patients: 1004 receiving ESPB and 1011 receiving PVB. Risk of bias was judged as low in nine studies, with some concerns in ten, and high in eight. 17 RCTs (including 1021 patients) reported 24-hour opioid consumption. There was no significant difference between ESPB and PVB (MD = 0.62 MME, 95% CI -0.23 to 1.46; p = 0.152, *I*^2^ = 86.8%) (Table 1, Fig. 1). Egger’s test suggested possible small-study effects (p = 0.023). TSA suggested that the cumulative evidence crossed the required information size. However, this result should not be interpreted as definitive, given the very low certainty of evidence, substantial heterogeneity, and possible small-study effects.

**Table 1:** Overall analysis.

Outcome	N	ESPB	PVB	Total effect (95% CI)	*I*^2^ (%)	p-value	Publication bias
**Main outcome**							
**MME**	17	508	513	MD 0.62 (-0.23; 1.46)	86.8	0.152	0.023
**Pain at rest at extubation**	16	495	500	MD 0.23 (0.01; 0.45)	81.4	0.039	0.085
**Pain at rest at 6h**	16	501	500	MD 0.02 (-0.24; 0.28)	81.1	0.905	0.737
**Pain at rest at 12h**	17	559	558	MD 0.00 (-0.31; 0.32)	91	0.995	0.913
**Pain at rest at 24h**	16	501	500	MD -0.01 (-0.23; 0.22)	77.1	0.965	0.449
**Pain on movement at extubation**	8	235	240	MD 0.36 (-0.11; 0.84)	82.1	0.132	Not assessed
**Pain on movement at 6h**	7	211	210	MD 0.23 (-0.12; 0.58)	80.7	0.204	Not assessed
**Pain on movement at 12h**	7	211	210	MD 0.34 (-0.03; 0.71)	82.8	0.060	Not assessed
**Pain on movement at 24h**	7	211	210	MD 0.44 (-0.06; 0.93)	84.6	0.080	Not assessed
**PONV**	13	409	408	OR 0.93 (0.61; 1.42)	0	0.737	0.448
**Rescue Analgesics**	6	164	163	OR 1.13 (0.63; 2.05)	42.7	0.675	Not assessed

ESPB, Erector Spinae Plane Block; PVB, Paravertebral Block; CI, Confidence Interval; N, Number of studies; MD, Mean Difference; MME, Morphine Milligram Equivalents; OR, Odds Ratio; PONV, Postoperative Nausea and Vomiting.

**Figure 1:**
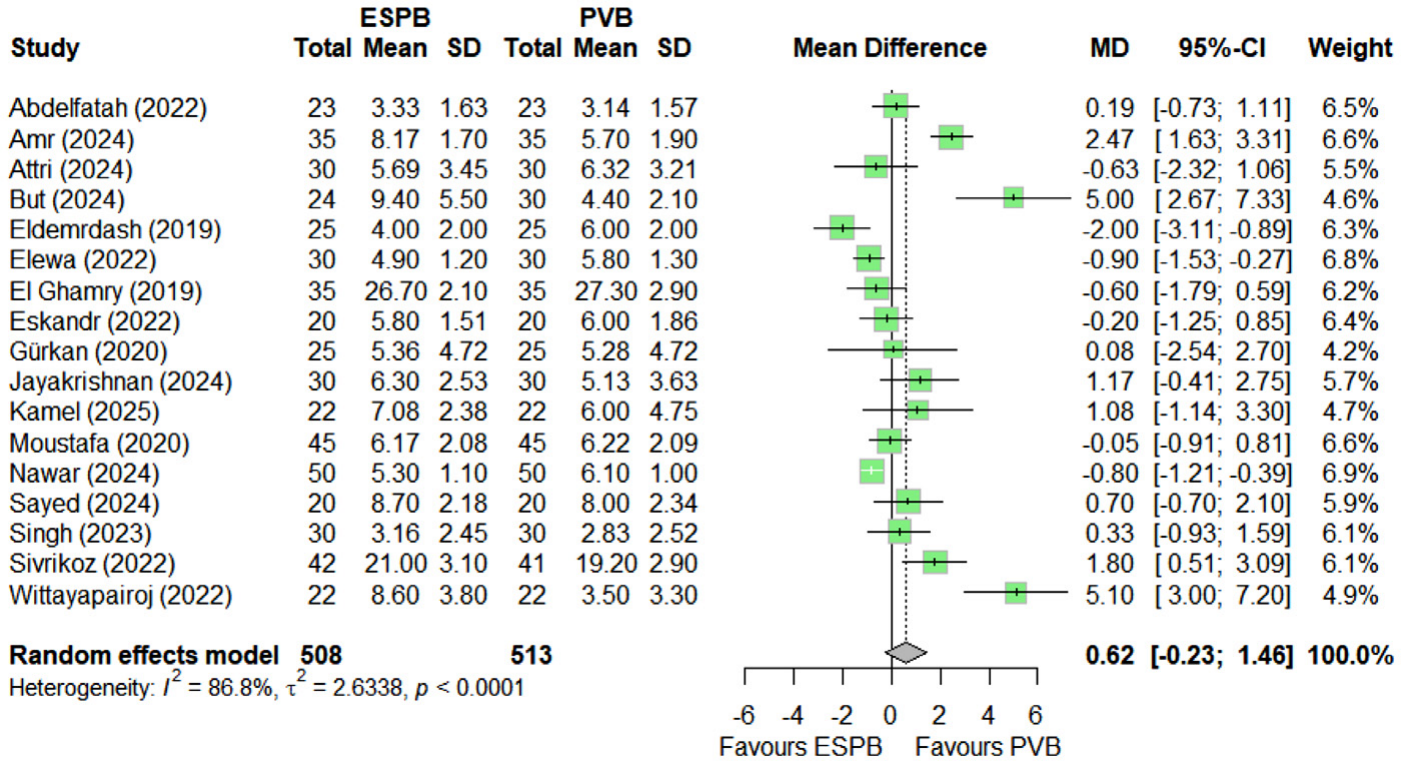
Forest plots of milligrams of morphine equivalents at 24 hours between the groups.

Subgroup analyses by surgery type and axillary involvement did not show significant effect modification. Sensitivity analysis excluding studies at high risk of bias produced results consistent with the primary analysis. Post-hoc meta-regression suggested that country, patient age, and local anesthetic choice may contribute to heterogeneity. However, these findings should be considered exploratory because they were based on study-level data and may be confounded by differences in surgical practice, block technique, perioperative analgesic protocols, and local anesthetic regimens.

Pain outcomes were generally comparable between techniques. Although pain at rest at extubation statistically favored PVB, no significant differences were observed at rest at 6, 12, or 24 hours, or during movement at any assessed time point. Similarly, PONV (OR = 0.93, 95% CI 0.61 to 1.42; *I*^2^ = 0%) and rescue analgesic requirement (OR = 1.13, 95% CI 0.63 to 2.05; *I*^2^ = 42.7%) did not differ significantly between groups (Table 1).

Overall, the available head-to-head randomized evidence does not demonstrate a clinically meaningful difference between ESPB and PVB for postoperative analgesia after breast surgery. However, these findings should not be interpreted as evidence of true equivalence. Confidence in the pooled estimate is limited by very low certainty of evidence, substantial heterogeneity, possible small-study effects, and variability in block technique, local anesthetic regimen, perioperative analgesic protocols, and outcome assessment.

## Study registration

PROSPERO: CRD420251118777.

## Data availability statement

This study was based on aggregate data extracted from previously published randomized trials. All data generated or analyzed during this study are included in this article and its Supplementary Material. Additional analytic materials are available from the corresponding author upon reasonable request.

## Authors’ contributions

BD, ET and YEK conceptualized the manuscript. BD, YEK, CK, ET, MB and ADC collected data and wrote the first draft. CK, BD and ADC conducted literature review and assisted in data analysis. YEK, ET, MB, and BD wrote the final version of the manuscript with critical revisions from ADC and CK. All authors contributed significantly to the study’s design, conduct, and analysis. All authors reviewed and approved the final version of the manuscript for publication.

## Funding

None.

## Appendix Supplementary materials

Image, application 1

## Footnotes

Supplementary material associated with this article can be found in the online version at doi:10.1016/j.bjane.2026.844767.
