# Supplementary material for: Erector spinae plane block vs. paravertebral block for postoperative analgesia in breast surgery: a meta-analysis of randomized trials
Source: Braz J Anesthesiol. 2026 May 28;76(4):844767. doi: 10.1016/j.bjane.2026.844767 (PMC13264338; doi:10.1016/j.bjane.2026.844767)
Supplement: Supplementary file 1 [file mmc1.pdf]

Erector spinae plane block vs paravertebral block for postoperative analgesia in breast surgery: a meta-analysis of randomized trials

Contents

Included studies 2

Search strategy 6

PRISMA flow diagram 7

Excluded studies 8

Study characteristics 9

Risk of Bias assessment 17

Forest and Funnel plots for both primary and secondary outcomes and sensitivity analysis 53

Sensitivity analysis for primary outcome 75

Trial sequential analysis 76

GRADE assessment 77

Meta-regression 78

Subgroup analysis and sensitivity analysis 79

Included studies

1. Eldemrdash AM, Abdelzaam ES. By ultrasonic-guided erector spinae block, thoracic paravertebral block versus serratus anterior plane block by articaine with adrenaline during breast surgery with general anesthesia: a comparative study of analgesic effect post-operatively: double-blind randomized controlled trial. *Open J Anesthesiol* 2019; 9(4):68–82.
2. El Ghamry MR, Amer AF. Role of erector spinae plane block versus paravertebral block in pain control after modified radical mastectomy: a prospective randomised trial. *Indian J Anaesth* 2019; 63:1008–1014.
3. Moustafa MA, Alabd AS, Ahmed AMM, Deghidy EA. Erector spinae versus paravertebral plane blocks in modified radical mastectomy: randomised comparative study of the technique success rate among novice anaesthesiologists. *Indian J Anaesth* 2020; 64:49–54.
4. Swisher MW, Wallace AM, Sztain JF, et al. Erector spinae plane versus paravertebral nerve blocks for postoperative analgesia after breast surgery: a randomized clinical trial. *Reg Anesth Pain Med* 2020; 45:260–266.
5. Gürkan Y, Aksu C, Kuş A, Yörükoğlu UH. Erector spinae plane block and thoracic paravertebral block for breast surgery compared to IV morphine: a randomized controlled trial. *J Clin Anesth* 2020; 59:84–88.
6. Varshney A. Comparison of paravertebral block vs erector spinae plane block in breast surgery patients. *J Adv Med Dent Sci Res* 2020; 8(10):192–195.
7. Agarwal S, Bharati SJ, Bhatnagar S, et al. The comparison of the efficacy of ultrasound-guided paravertebral block versus erector spinae plane block for postoperative analgesia in modified radical mastectomy: a randomized controlled trial. *Saudi J Anaesth* 2021; 15:137–143.

8. Elewa AM, Faisal M, Sjöberg F, Abuelnaga ME. Erector spinae plane block versus paravertebral block in analgesic outcomes following breast surgery. *BMC Anesthesiol* 2023; 23(1):19.
9. Sivrikoz N, Turhan Ö, Ali A, Altun D, Tükenmez M, Sungur Z. Paravertebral block versus erector spinae plane block for analgesia in modified radical mastectomy: a randomized, prospective, double-blind study. *Minerva Anestesiol* 2022; 88:1003–1012.
10. Wittayapairoj A, Sinthuchao N, Somintara O, Thincheelong V, Somdee W. A randomized double-blind controlled study comparing erector spinae plane block and thoracic paravertebral block for postoperative analgesia after breast surgery. *Anesth Pain Med (Seoul)* 2022; 17:445–453.
11. Anandhkumar A, Lenin P, Arulrajan S. Role of presurgical erector spinae plane block versus paravertebral block in pain control and hemodynamic stability after modified radical mastectomy: a prospective randomized trial. *IJMSCR* 2022; 5(6):611–619.
12. Eskandr A, Mahmoud K, Kasemy Z, Mohamed K, Elhennawy T. A comparative study between ultrasound-guided thoracic paravertebral block, pectoral nerves block, and erector spinae block for pain management in cancer breast surgeries: a randomized controlled study. *Rev Esp Anestesiol Reanim (Engl Ed)* 2022; 69:617–624.
13. Abdelfatah A, Abo Elanin T, Hamroush A. Comparative study between ultrasound-guided erector spinae plane block versus paravertebral block for postoperative pain relief in patients undergoing unilateral modified radical mastectomy. *Al-Azhar Int Med J* 2022; 3(4):Article 10.
14. Santonastaso DP, de Chiara A, Righetti R, et al. Efficacy of bi-level erector spinae plane block versus bi-level thoracic paravertebral block for postoperative analgesia in modified radical mastectomy: a prospective randomized comparative study. *BMC Anesthesiol* 2023; 23(1):209.

15. Kangle S, Dudhedia U, Pradhan AS, Nair AS. Prospective comparative analysis of intraoperative and postoperative anesthetic and analgesic effect of ultrasound-guided pectoral with serratus anterior plane block versus thoracic paravertebral versus erector spinae blocks in breast oncosurgeries. *Indian Anaesth Forum* 2023; 24(2):96–102.
16. Singh A, Agrawal N, Baruah U, Sandill S. Comparison of efficacy of erector spinae plane block with paravertebral block for postoperative analgesia in patients undergoing breast cancer surgery. *MAMC J Med Sci* 2023; 9(2):105–114.
17. Sayed JA, Hamed R, Abdelraouf AM, El-Hagagy NYM, El Dean Mousa MB, Abdel-Wahab AH. A comparative study of respiratory effects of erector spinae plane block versus paravertebral plane block for women undergoing modified radical mastectomy. *BMC Anesthesiol* 2024; 24(1):262.
18. But M, Wernicki K, Zieliński J, Szczecińska W. A comparison of the effectiveness of the serratus anterior plane block and erector spinae plane block to that of the paravertebral block in the surgical treatment of breast cancer: a randomized, prospective, single-blinded study. *J Clin Med* 2024; 13(16):4836.
19. Jayakrishnan S, Dua A, Kumar A. Comparison of fascial plane blocks (ESPB vs TPVB) for pain relief following modified radical mastectomy. *J Anaesthesiol Clin Pharmacol* 2024; 40:410–415.
20. CH NR, Youssef GF, Ahmed AA, Abd El-Malek OR, Ahmed RA, Nawar NR. Comparative study between the analgesic efficacy of pectoralis major block II versus erector spinae block versus paravertebral block in postoperative analgesia in modified radical mastectomy. *Rev Chil Anest* 2024; 53(6):605–613.
21. Attri JP, Khetarpal R, Kaur H, Kaur K. Bi-level ultrasound-guided erector spinae block versus paravertebral block in postoperative analgesia after breast cancer surgeries. *Res J Med Sci* 2024; 18:486–492.

22. Yendrapati C, Gopalakrishnaiah C, Vindhya K, Mylabathula NJ. Comparison of feasibility of ultrasound-guided erector spinae plane block versus paravertebral plane block in modified radical mastectomies by anaesthesiology residents: a randomised control trial. *J Cardiovasc Dis Res* 2024; 15(2):147–155.

23. Amr SA, Othman AH, Ahmed EH, Naeem RG, Kamal SM. Comparison between ultrasound-guided erector spinae plane block and paravertebral block on acute and chronic post mastectomy pain after modified radical mastectomy: randomized controlled trial. *BMC Anesthesiol* 2024; 24(1):420.

24. Raft J, Dureau S, Fuzier R, et al. Erector spinae plane block versus paravertebral block for major oncological breast surgery: a multicentre randomised controlled trial. *Br J Anaesth* 2025; 135(3):772–778.

25. Kumar BA, Divyalakshmi T, Brindha R, Pandian N, Periasamy P. Comparison of efficacy of ultrasound-guided erector spinae block vs paravertebral block for postoperative analgesia in breast surgeries. *J Pharm Bioallied Sci* 2025; 17(Suppl 2):S1927–S1929.

26. Kamel MA, Ismael EAM, El Sheikh SMAA, Elrawas MM, Gendy EHS. Ultrasound-guided erector spinae plane block versus thoracic paravertebral block for perioperative analgesia for modified radical mastectomy: a randomized trial. *Anaesth Pain Intensive Care* 2025; 29(4):292–300.

27. Muaziz SS, Khan MJ, Noreen N, Iftikhar SH. Postoperative pain relief in patients undergoing unilateral modified radical mastectomy; comparison between ultrasound-guided erector spinae plane block versus ultrasound-guided thoracic paravertebral block. *Insights J Health Rehabil* 2025; 3(2):21–28.

Search Strategy

**PubMed**
("erector spinae block" OR "erector spinae plane block" OR "ESP block" OR ESPB)<br>AND<br>(“paravertebral block” OR “paravertebral nerve block” OR “thoracic paravertebral block” OR “TPVB” OR “PVB”)<br>AND<br>(“breast surgery” OR “breast cancer surgery” OR “mastectomy” OR “lumpectomy” OR “breast excision”)
**Scopus**
TITLE-ABS-KEY (("erector spinae block" OR "erector spinae plane block" OR "ESP block" OR ESPB) AND<br>("paravertebral block" OR "paravertebral nerve block" OR "thoracic paravertebral block" OR TPVB OR PVB) AND<br>("breast surgery" OR "breast cancer surgery" OR mastectomy OR lumpectomy OR "breast excision"))
**Embase**
('erector spinae block':ti,ab,kw OR 'erector spinae plane block':ti,ab,kw OR 'esp block':ti,ab,kw OR espb:ti,ab,kw) AND ('paravertebral block':ti,ab,kw OR 'paravertebral nerve block':ti,ab,kw OR 'thoracic paravertebral block':ti,ab,kw OR tpvb:ti,ab,kw OR pvb:ti,ab,kw) AND ('breast surgery':ti,ab,kw OR 'breast cancer surgery':ti,ab,kw OR mastectomy:ti,ab,kw OR lumpectomy:ti,ab,kw OR 'breast excision':ti,ab,kw)
**Cochrane Central Register of Controlled Trials (CENTRAL)**
("erector spinae block" OR "erector spinae plane block" OR "ESP block" OR ESPB) AND ("paravertebral block" OR "paravertebral nerve block" OR "thoracic paravertebral block" OR TPVB OR PVB) AND ("breast surgery" OR "breast cancer surgery" OR mastectomy OR lumpectomy OR "breast excision")
**Web of Science**
("erector spinae block" OR "erector spinae plane block" OR "ESP block" OR ESPB) AND ("paravertebral block" OR "paravertebral nerve block" OR "thoracic paravertebral block" OR TPVB OR PVB) AND ("breast surgery" OR "breast cancer surgery" OR mastectomy OR lumpectomy OR "breast excision")
We also reviewed the abstracts of the American Society of Regional Anesthesia (ASRA), European Society of Regional Anaesthesia (ESRA), the Turkish Anesthesiology and Reanimation Congress (TARK), and the Italian Society of Anesthesia, Analgesia, Resuscitation and Intensive Care (SIAARTI) congresses that took place in the past 5 years and specifically analyzed those that were relevant to the subject.

PRISMA 2020 flow diagram

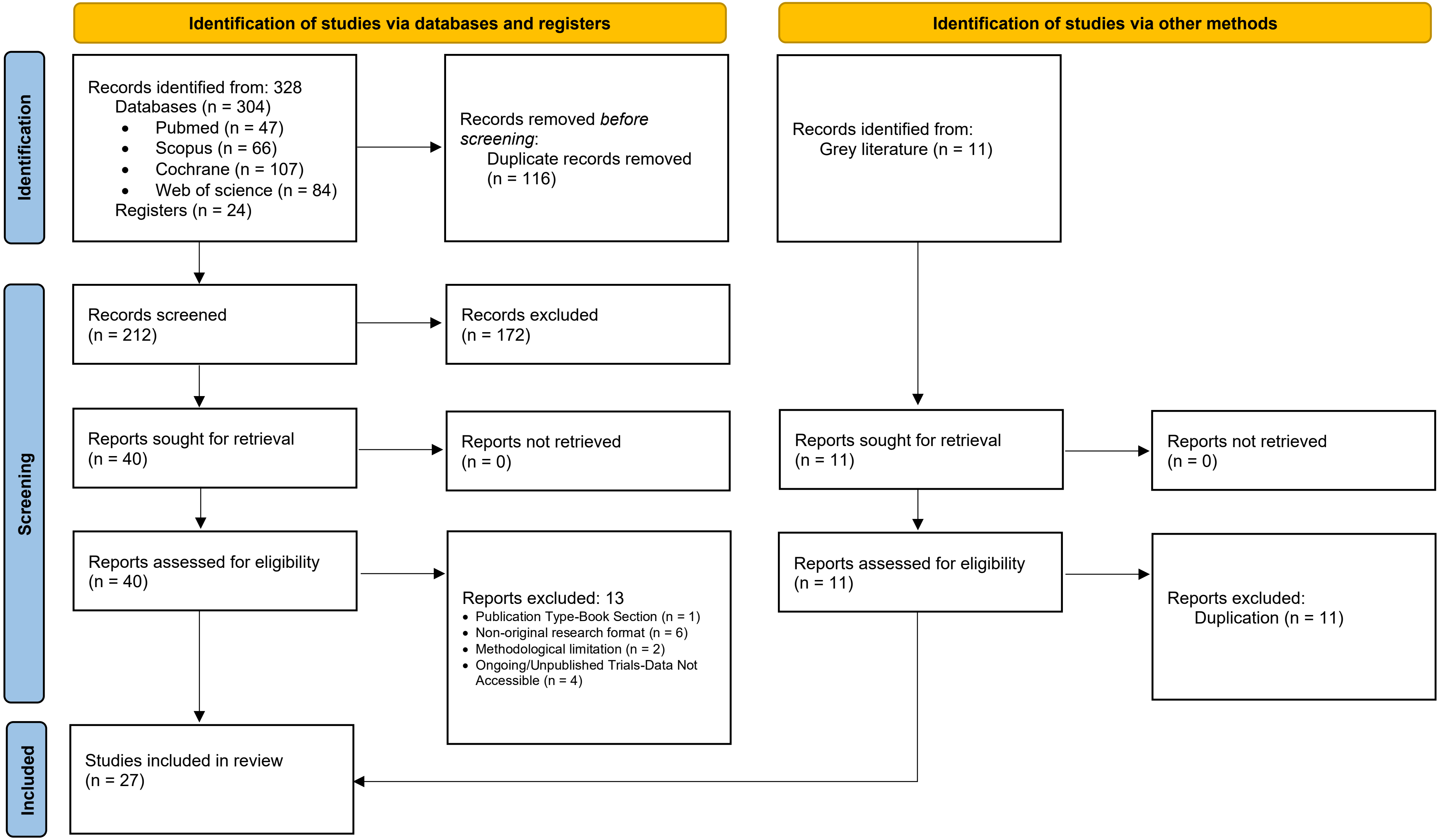

Excluded Studies

1. Ben Aziz M, Hendrix JM, Mukhdomi T. Regional Anesthesia for Breast Reconstruction. 2023 Jul 18. In: StatPearls [Internet]. Treasure Island (FL): StatPearls Publishing; 2025 Jan–.
2. Chin KJ. Erector spinae plane and paravertebral blocks have similar opioid-sparing effects following breast surgery. Reg Anesth Pain Med. 2020 Mar 26:rapm-2020-101365. https://doi.org/10.1136/rapm-2020-101365.
3. De Cassai A, Aksu C, Tulgar S, Ahiskalioglu A. ESP block compared to paravertebral block in breast surgery. Minerva Anestesiol. 2020 Oct;86(10):1116-1117. https://doi.org/10.23736/S0375-9393.20.14630-3.
4. Elewa AM, Faisal M, Sjöberg F, Abuelnaga ME. Erector spinae plane block versus paravertebral block in analgesic outcomes following breast surgery. BMC Anesthesiol. 2023 Jan 11;23(1):19. https://doi.org/10.1186/s12871-022-01950-9.
5. Li XT, Xue FS, Tian T. Is really thoracic paravertebral block superior to erector spinae plane block for pain control after modified radical mastectomy? Minerva Anestesiol. 2023 Sep;89(9):837-839. https://doi.org/10.23736/S0375-9393.23.17283-X.
6. Sethuraman RM. Erector spinae plane block versus paravertebral block in breast surgeries. BMC Anesthesiol. 2022 Dec 28;22(1):408. https://doi.org/10.1186/s12871-022-01946-5.
7. Smits R, Fenten M, Filippini-De Moor G, Van Geffen GJ. Paravertebral block versus erector spinae plane block in patients undergoing breast cancer surgery: a feasibility study. Reg Anesth Pain Med. 2019;44(10):A274. https://doi.org/10.1136/rapm-2019-ESRAABS2019.502.
8. Venu SB, Malick BY, Venugopalan PG. ESPB vs TPVB in modified radical mastectomy. IJAR, 2024, 6.1: 27-31. https://doi.org/10.33545/26648849.2024.v6.i1a.40.
9. Stewart, J. W., Ringqvist, J., Wooldridge, R. D., et al. P. (2021). Erector spinae plane block versus thoracic paravertebral block for pain management after total bilateral mastectomies. Baylor University Medical Center Proceedings, 34(5), 571–574. https://doi.org/10.1080/08998280.2021.1919003.
10. ChiCTR1800015217.
11. NCT03471442.
12. NCT04498234.
13. NCT05590559.

Study characteristics

Author (Year)	Sample size	Country	Age	Surgery	Axilla	Block Characteristics	Adjuvants	General Anaesthesia Protocol	Analgesia Protocol	Main outcome	Conflict of interest
**Eldemrdash (2019)[17]**	**ESPB**:25<br>**PVB**:25	Egypt	18-65	MRM	Yes	2% Artacaine<br>**ESPB**: 20 mL<br>**PVB**: 20 mL<br>**Level:** T4-T5	Adrenaline	**PREMEDICATION:** Midazolam 2 mg IV<br>**INDUCTION**: Fentanyl 2 µg/kg IV, Propofol 2.5 mg/kg IV, Atracurium 0.15 mg/kg IV<br>**MAINTENANCE**: O_2 + Isoflurane 1%, Paracetamol 1 g IV, $ETCO_2$ 35–45 mmHg<br>**REVERSAL**: Ondansetron 4 mg IV, Neostigmine 40 µg/kg IV, Atropine 1 mg IV	PCA Protocol: IV morphine (1 mg/ml) PCA: bolus 1 mg, lockout 10 min, max 4 mg/h.	Postoperative pain scores	No
**El Ghamry (2019)[18]**	**ESPB**:35<br>**PVB**:35	Egypt	20-60	MRM	Yes	0.25% Bupivacaine<br>**ESPB**:20 mL<br>**PVB**: 20 mL<br>**Level:** T5	-	**INDUCTION**: Fentanyl 1 µg/kg, Propofol 2 mg/kg, Cisatracurium 0.15 mg/kg<br>**MAINTENANCE**: Isoflurane 1.5–2%, O_2–air mix, Cisatracurium 0.03 mg/kg as needed, Fentanyl 1 µg/kg for HR/MBP ↑ >20%<br>**REVERSAL**: Neostigmine 2.5 mg + Atropine 1 mg	Morphine 0.1 mg/kg IV for VAS >3	Morphine consumption 24 h	No
**Moustafa (2020)[19]**	**ESPB**:45<br>**PVB**:45	Egypt	40-65	MRM	Yes	0.25% Bupivacaine<br>**ESPB**: 20 mL<br>**PVB**: 20 mL<br>**Level:** T4	-	**INDUCTION**: 2 mg midazolam and 1 $\mu g.kg^{-1}$ fentanyl, 2 $mg.kg^{-1}$ propofol and atracurium 0.25 $mg.kg^{-1}$<br>**MAINTENANCE**: isoflurane 1% in 50% oxygen air mixture	Paracetamol 1 g IV q6h; pain ≥4 treated with morphine 2 mg IV every 5 min until pain <4.	Assess the success rate	No
**Swisher (2020)[20]**						0,5 % Ropivacain<br>**ESPB**: 20 mL for unilateral, 16 mL			Fentanyl 25 µg IV for NRS <5; fentanyl 50 µg	Pain scores within the recovery	

	**ESPB**:50<br>**PVB**:50	USA	>18	Both	Both	on each side for bilateral surgeries<br>**PVB**: like ESPB<br>**Level:** T3 for involving axillary work-T4 for not involving axillay work	Epinephrin e	A combination of inhaled and intravenous anesthetics	IV for NRS ≥5; hydromorphone 0.5 mg IV if fentanyl ineffective; oxycodone 5 mg PO for NRS 4–6 if oral tolerated.	room and opioid requirements in the operating and recovery rooms	Yes
**Gurkan (2020)**[21]	**ESPB**:25<br>**PVB**:25	Türki ye	25-65	Both	NI	0.25% Bupivacaine<br>**ESPB**: 20 mL<br>**PVB**: 20 mL<br>**Level:** T4	-	**INDUCTION**: Propofol 2–3 mg/kg, Fentanyl 2 µg/kg, Rocuronium 0.6 mg/kg<br><br>**MAINTENANCE**: Desflurane + N_2O/O_2 (2:1) with 3 L fresh gas flow	PCA: Morphine 0.5 mg/mL (1 mg bolus, 8 min lockout, 6 mg/h limit) Paracetamol 1 g IV q6h	Total morphine consumption 24 h	No
**Varshney (2020)**[22]	**ESPB**:30<br>**PVB**:30	India	20-50	Ma	NI	**ESPB**:NI<br>**PVB**: NI<br>**Level:**NI	NI	NI	NI	NI	NI
**Agarwal (2021)**[23]	**ESPB**:40<br>**PVB**:40	India	18-70	MRM	Yes	0,5% Ropivacaine<br>**ESPB**: 20 mL<br>**PVB**: 20 mL<br>**Level:** T5-T4	-	**INDUCTION**: 2 µg/kg fentanyl, 1–2 mg/kg propofol, and 0.6 mg/kg rocuronium<br>**MAINTENANCE**: 50% air and oxygen and desflurane (0.8–1 MAC)	Paracetamol 1 g IV 30 min before end of surgery, then 20 mg/kg IV q6h. If NRS >4 → diclofenac 1.5 mg/kg IV; if persistent → tramadol 1 mg/kg IV (repeat as needed, max 100 mg/6h or 400 mg/24h)	Duration of postoperative analgesia (that is, time to first analgesic request from the time of giving block)	No

**Elewa (2022)**[24]	**ESPB**:30<br>**PVB**:30	Egypt	20-60	MRM	Yes	0.25% Bupivacaine<br>**ESPB**: 30 mL<br>**PVB**: 30 mL<br>**Level:** T3-T4		**INDUCTION**: fentanyl (2 mcg.kg^{-1}), propofol (2 mg.kg^{-1}), and cisatracurium (0.15 mg.kg^{-1})<br>**MAINTENANCE**: 1–2 MACs of isoflurane in 2L of 50% oxygen and air mixture, cisatracurium (0.03 mg. kg^{-1})	IV paracetamol 1 g 30 min before end of surgery; PCA morphine (1 mg bolus, 10-min lockout, max 5 mg/h); ketorolac 30 mg IV q12h alternating with paracetamol 1 g IV q12h for 48 h	Total morphine consumption 24 h	No
**Sivrikoz (2022)**[25]	**ESPB**:42<br>**PVB**:41	Türkiye	18-75	MRM	Yes	0,375% Bupivacaine<br>**ESPB**: 20 mL<br>**PVB**: 20 mL<br>**Level:** T4	-	**INDUCTION**: 0.03 mg.kg^{-1} midazolam, 2 mcg.kg^{-1} fentanyl, 2 mg.kg^{-1} propofol and 0.6 mg.kg^{-1} rocuronium<br>**MAINTENANCE**:<br>2-3% sevoflurane in a mixture of 40% O_2 and 60% N_2O	At skin closure: IV paracetamol 1 g + IV morphine 0.03 mg/kg (loading). PCA morphine for 24 h (basal 0.01 mg/kg/h, demand 1 mg, lockout 20 min). Rescue: IV tramadol 0.5 mg/kg if NRS ≥4.	Total morphine consumption 24 h	No
**Wittayapairoj (2022)**[26]	**ESPB**:22<br>**PVB**:22	Thailand	18-75	Ma	Both	0,5% Levobupivacaine<br>**ESPB**: 20 mL<br>**PVB**: 20 mL<br>**Level:** T4	-	**INDUCTION**: Propofol 2–3 mg/kg and fentanyl 1.5–2 μg/kg, cisatracurium 0.1–0.2 mg/kg<br>**MAINTENANCE**: mixture of air-oxygen (FiO_2 0.4) and sevoflurane, fentanyl administered in increments of 25 μg	At end of surgery: IV morphine 3 mg. On-demand: IV morphine 3 mg bolus if NRS >3. Oral acetaminophen 1000 mg q6h +	Total morphine consumption 24 h	No

									IV morphine 2 mg bolus if NRS >3		
**Anandhkumar (2022)**[27]	**ESPB**:27 **PVB**:27	India	20-70	MRM	Yes	0.25% Bupivacaine **ESPB**: 20 mL **PVB**: 20 mL **Level:** T5	-	Standardized general anaesthesia after the block **REVERSAL**: IV neostigmine 50 mcg/kg and glycopyrrolate 10 mcg/kg.	NI	Time to first analgesic request, total intraoperative fentanyl consumption, and total postoperative opioid consumption	No
**Eskandr (2022)**[28]	**ESPB**:20 **PVB**:20	Egypt	18-60	BS	No	0.25% Bupivacaine **ESPB**: 25 mL **PVB**: 25 mL **Level:** T5-T3		**INDUCTION**: IV fentanyl 1 µg/kg, IV propofol 1.5–2 mg/kg until loss of verbal response, IV atracurium 0.5 mg/kg to facilitate intubation. **MAINTENANCE**: Oxygen + isoflurane (MAC 1–1.2)	Max morphine: 10 mg in PACU, 3 mg in ward	Duration of analgesia	No
**Abdelfatah (2022)**[29]	**ESPB**:23 **PVB**:23	Egypt	45-83	MRM	Yes	0.25% Bupivacaine **ESPB**: 30 mL **PVB**: 30 mL **Level:** T4	-	**PREMEDICATION:** IV midazolam (0.05 mg/kg) and fentanyl (25ug) **INDUCTION**: IV midazolam (0.05 mg/kg) and fentanyl (25ug) **MAINTENANCE**: Isoflurane 1–2% in 100% O_2; cisatracurium 0.03 mg/kg (TOF-guided, TOF=2); fentanyl 25 µg if HR/MBP >20% **REVERSAL**: Atropine 0.01 mg/kg + neostigmine 0.04 mg/kg	PCA: Nalbuphine 1 mg/mL (2 mL bolus, 10 min lockout, no basal) + ketorolac 30 mg IV q8h	Intensity of postoperative pain	NI
**Santonastaso (2023)**[30]	**ESPB**:41 **PVB**:41	Italy	18-90	Both	Both	**ESPB**: 24 mL Bupivacaine 0.5% (T2 and T5 bilevel) **PVB**: 16 mL Bupivacaine 0.75%	-	**INDUCTION**: Propofol 2 mg/kg IV, Rocuronium 0.6 mg/kg	Acetaminophen 1 g IV 30 min before end of surgery, then q8h. Pain (rest	NRS pain score 12 h after surgery.	No

						(T2- T3 and T4-T5 bilevel)		**MAINTENANCE**: Propofol infusion 6–9 mg/kg/h, Fentanyl 100 µg IV bolus for HR/MBP ↑ >20%	& movement, NRS) assessed at 0, 2, 6, 12, 24, 36 h. If NRS >3 → ketorolac 30 mg IV (max 3/day); if pain persists ≥30 min → morphine 2 mg IV		
**Kangle (2023)**[31]	**ESPB**:25 **PVB**:25	Oman	18-60	MRM	Yes	20 ml 0.25% bupivacaine + 4.5 ml 2% lidocaine + 0.5 ml clonidine **ESPB**:25 mL **PVB**:25 mL **Level:** T5	Clonidine	**PREMEDICATION:** Glycopyrrolate 4 µg/kg IV, midazolam 0.02 mg/kg IV **INDUCTION**: Propofol 2.0–2.5 mg/kg IV **MAINTENANCE**: 50% air in oxygen with sevoflurane	IV paracetamol 500 mg, IV dexamethasone 8 mg, and magnesium sulfate 1 g in 500 ml were administered	Postoperative pain scores	No
**Singh (2023)**[32]	**ESPB**:30 **PVB**:30	India	18-60	MRM	Yes	0.5% Ropivacaine **ESPB**:22 mL **PVB**:22 mL **Level:** T4, T3-T4	Clonidine	**INDUCTION**: Fentanyl 2 µg/kg IV, Propofol 2–2.5 mg/kg, Vecuronium 0.1 mg/kg **MAINTENANCE**: N_2O 67% / O_2 33% + Isoflurane 0.6–0.8% with supplemental Vecuronium **REVERSAL**: Neostigmine 0.05 mg/kg and glycopyrrolate 0.01 mg/kg	Paracetamol 1 g IV q8h; Tramadol 50 mg IV for VAS >3, repeat after 30 min if needed (max 400 mg/24 h)	Duration of analgesia (block to first rescue analgesia)	No
**Sayed (2024)**[33]	**ESPB**:20 **PVB**:20	Egypt	18-50	MRM	Yes	0.5% Bupivacaine **ESPB**: 20 mL **PVB**:20 mL **Level:** T4-5, T4	-	**PREMEDICATION:** Alprazolam 0.25 mg **INDUCTION**: IV fentanyl 1.5 µg/kg, propofol 1.5–2 mg/kg, cis-atracurium 0.15 mg/kg **MAINTENANCE**: Isoflurane 1–1.5 MAC in 50% air:O_2 cis-atracurium 0.03 mg/kg bolus (TOF-guided)	IV nalbuphine 0.1 mg/kg for VNRS ≥ 4	ESPB vs TPVB on FVC in females after MRM at 24 h	No

**But (2024)**[34]	**ESPB**:24 **PVB**:30	Poland	18-85	MRM	Yes	10 mL of 2% lidocaine with 10 mL of 0.5% bupivacaine **ESPB**: 20 mL **PVB**: 20 mL **Level:** T5	-	**INDUCTION**: Propofol 2 mg/kg IV, Rocuronium 0.6 mg/kg IV **MAINTENANCE**: Inhaled desflurane	NI	Compare to efficacy of the blocks	No
**Jayakrishnan (2024)**[35]	**ESPB**:30 **PVB**:30	India	>18	MRM	Yes	0.25% Bupivacaine **ESPB**: 20 mL **PVB**: 20 mL **Level:** T5	-	**INDUCTION**: Propofol (1–2 mg/kg) and fentanyl (1.5–2.0 μg/kg), atracurium 0.5 mg/kg **MAINTENANCE**: Sevoflurane	Paracetamol 1 g IV after procedure. PCA morphine: 1 mg bolus, 5-min lockout, max 4 doses/h	To compare the analgesia provided by ESPB and TPVB	No
**Nawar (2024)**[36]	**ESPB**:50 **PVB**:50	Egypt	30-70	MRM	Yes	0.25% Bupivacaine **ESPB**: 20 mL **PVB**: 20 mL **Level:** T4	-	**INDUCTION**: Propofol 1.5–2 mg/kg IV, fentanyl 1 μg/kg IV, atracurium 0.5 mg/kg IV for intubation **MAINTENANCE**: Isoflurane 1.2–2% with IV fluids	Morphine 2 mg IV was given until VAS ≤3	Postoperative pain scores	No
**Attri (2024)**[37]	**ESPB**:30 **PVB**:30	India	20-60	Both	NI	0.25% Bupivacaine **ESPB**: 15 mL **PVB**: 15 mL **Level:** T2 and T6 (bilevel)	Dexamethasone	NI	Fentanyl 1 μg/kg IV for VAS >3; if inadequate, Diclofenac 75 mg IM	Block time, analgesia duration (VAS), total rescue analgesia	NI
**Yendrapati (2024)**[38]	**ESPB**:50 **PVB**:50	India	40-65	MRM	NI	0.25% Bupivacaine **ESPB**: 20 mL **PVB**:20 mL **Level:** T4	-	NI	Morphine IV was given within 2 h if VAS >3, repeated until VAS = 3	Block success, ease, hemodynamic response, postop analgesia,	NI

										duration, and adverse effects	
**Amr (2024)**[39]	**ESPB**:35 **PVB**:35	Egypt	20-70	MRM	Yes	0.25% Bupivacaine **ESPB**: 20 mL **PVB**: 20 mL **Level:**T4, T2-4-6	-	**INDUCTION**: fentanyl 0.5 μg/kg, propofol 2 mg/kg, and atracurium 0.5 mg/kg **MAINTENANCE**: 1–1.5 MAC isoflurane with O_2/air (FiO_2 0.5); muscle relaxant every 20 min.	Initial morphine 2 mg IV if pain or VAS ≥3; PCA lockout 5 min, no background infusion.	Total morphine consumption 24 h	No
**Raft (2025)**[40]	**ESPB**:145 **PVB**:147	France	18-85	Both	Yes	0,5 % Ropivacain **ESPB**: 30 mL **PVB**:30 mL **Level:** T3, T2-3	-	**INDUCTION**: propofol and remifentanil (first targeting a 1 ng/ml effect-site concentration; bolus of 0.3 μg/kg remifentanil)	Paracetamol 1 g IV, ketoprofen 1 mg/kg IV, ondansetron 4 mg IV. Morphine 1 mg IV q5min if VAS >3. Oral paracetamol 1 g four times daily + ketoprofen 50 mg four times for 24 h. Oral tramadol 50 mg q4h for breakthrough pain (VAS >3)	Percentage of participants requiring morphine in the first 2 h in PACU	No
**Kumar (2025)**[41]	**ESPB**:30 **PVB**:30	India	18-60	Both	NI	0,125 % Ropivacaine **ESPB**: NI **PVB**:NI **Level:** T2-3 (ESPB)	Dexmedetomidine	NI	NI	Quality of analgesia and the duration of action of the blocks	No

**Kamel (2025)**[42]	**ESPB**:22<br>**PVB**:22	Egypt	18-65	MRM	Yes	0.25% Bupivacaine<br>**ESPB**:25 mL<br>**PVB**: 25 mL<br>**Level:** T5,T3	-	**INDUCTION**: Fentanyl 1 µg/kg IV, propofol 1.5–2 mg/kg, atracurium 0.5 mg/kg<br>**MAINTENANCE**: Sevoflurane 2%, fentanyl 0.1 µg/kg bolus if MBP >20% baseline	Ketorolac 30 mg q8h; rescue: Morphine 3 mg if VAS ≥ 4	Duration of analgesia	No
**Muaziz (2025)**[43]	**ESPB**:58<br>**PVB**:58	Pakistan	18-60	MRM	Yes	0.25% Bupivacaine<br>**ESPB**: 20-30 mL<br>**PVB**:20 mL<br>**Level:** NI	-	NI	NI	Postoperative pain scores	No

Abbrevations: ESPB: Erector Spinae Plane Block, PVB: Paravertebral Block, MRM: Modified Radical Mastectomy, Ma: Mastectomy, BS: Breast conservative surgery, NI: Not Informed, MBP: Mean Blood Pressure, MAC: Minimum Alveolar Concentration, FVC: Forced Vital Capacity, NRS: Numeric Rating Scale, PCA: Patient controlled analgesia, VAS: Visual Analogue Scale, VNRS: Verbal Numeric Rating Scale, IV: Intravenous, IM: Intramuscular, LA: Local Anesthetics, PACU: Post-Anesthesia Care Unit**,** TOF: Train-of-Four**,** USA: United States of America

Risk of Bias Assessment

Risk of bias domains

Study	D1	D2	D3	D4	D5	Overall
Eldemrdash (2019)	+	-	+	-	-	X
El Ghamry (2019)	-	+	+	+	-	-
Moustafa (2020)	+	-	+	+	+	-
Swisher (2020)	+	+	+	+	+	+
Gurkan (2020)	+	+	+	+	+	+
Varshney (2020)	-	X	+	X	-	X
Agarwal (2021)	+	+	+	+	+	+
Elewa (2022)	+	+	+	+	+	+
Sivrikoz (2022)	+	+	+	+	+	+
Wittayapairoj (2022)	+	+	+	+	+	+
Anandhkumar (2022)	+	-	+	X	-	X
Eskandr (2022)	+	+	+	+	+	+
Abdelfatah (2022)	+	-	+	-	-	X
Santonastaso (2023)	+	-	+	-	+	-
Kangle (2023)	+	-	+	-	+	-
Singh (2023)	+	-	+	-	+	-
Sayed (2024)	+	+	+	-	+	-
But (2024)	-	-	-	X	-	X
Jayakrishnan (2024)	-	-	+	-	+	-
Nawar (2024)	+	-	+	-	+	-
Attri (2024)	-	-	+	-	+	-
Yendrapati (2024)	+	-	X	-	-	X
Amr (2024)	+	+	+	+	+	+
Raft (2025)	+	+	+	+	+	+
Kumar (2025)	-	-	+	X	-	X
Kamel (2025)	+	+	+	-	-	-
Muaziz (2025)	-	-	+	X	-	X

Domains:
D1: Bias arising from the randomization process.
D2: Bias due to deviations from intended intervention.
D3: Bias due to missing outcome data.
D4: Bias in measurement of the outcome.
D5: Bias in selection of the reported result.

Judgement
X High
- Some concerns
+ Low

**TİTLE: 1- By Ultrasonic-Guided Erector Spinae Block, Thoracic Paravertebral Block versus Serratus Anterior Plane Block by Articaine with Adrenaline during Breast Surgery with General Anesthesia: A Comparative Study of Analgesic Effect Post-Operatively: Double Blind Randomized, Controlled Trial[17]**			
Domain	Subdomain	Risk of Bias	Justification
1.Bias arising from the randomisation process	1.1. Was the allocation sequence random?	Y/PY	The participants were randomly assigned into three groups 25 in each (ESB, PVB and SPB) by a random sequence number produced by the computer
	1.2. Was the allocation sequence concealed until participants were enrolled and assigned to interventions?	Y/PY	kept in sealed envelopes. The closed envelopes..
	1.3. Did baseline differences between intervention groups suggest a problem with the randomization process?	PN/N	…being comparable to each other with respect to age, weight, side, duration of surgery and ASA status.
	SUBRISK	LOW	
2. Bias due to deviations from intended interventions	2.1. Were participants aware of their assigned intervention during the trial?	NI	
	2.2. Were carers and people delivering the interventions aware of participants' assigned intervention during the trial?	NI	An investigator blinded to group allocation recorded pain scores at 0, 0.5, 1, 2, 4, 6, 8, 12, 24, 36, and 48 h after surgery.
	2.3. If Y/PY/NI to 2.1 or 2.2: Were there deviations from the intended intervention that arose because of the trial context?	PN/N	
	2.4 If Y/PY to 2.3: Were these deviations likely to have affected the outcome?	PN/N	
	2.5. If Y/PY/NI to 2.4: Were these deviations from intended intervention balanced between groups?	PN/N	
	2.6 Was an appropriate analysis used to estimate the effect of assignment to intervention?	Y/PY	
	2.7 If N/PN/NI to 2.6: Was there potential for a substantial impact (to the result) of the failure to analyse participants in the group to which they were randomized?		
		SOME CONCERNS	
Bias due to missing outcome data	3.1 Were data for this outcome available for all, or nearly all, participants randomized?	Y/PY	All of the 80 patients randomised were included in the analysis, no missing data.
	3.2 If N/PN/NI to 3.1: Is there evidence that the result was not biased by missing outcome data?		
	3.3 If N/PN to 3.2: Could missingness in the outcome depend on its true value?		
	3.4 If Y/PY/NI to 3.3: Is it likely that missingness in the outcome depended on its true value?		
		LOW	
Bias in measurement of the outcome	4.1 Was the method of measuring the outcome inappropriate?	Y/PY	VAS scores
	4.2 Could measurement or ascertainment of the outcome have differed between intervention groups?	PN/N	
	4.3 If N/PN/NI to 4.1 and 4.2: Were outcome assessors aware of the intervention received by study participants?	PN/N	Blinding of outcome assessors was not clearly reported
	4.4 If Y/PY/NI to 4.3: Could assessment of the outcome have been influenced by knowledge of intervention received?		
	4.5 If Y/PY/NI to 4.4: Is it likely that assessment of the outcome was influenced by knowledge of intervention received?		
		SOME CONCERNS	
Bias in selection of the reported result	5.1. Were the data that produced this result analysed in accordance with a pre-specified analysis plan that was finalized before unblinded outcome data were available for analysis?	Y/PY	No trial registration available; unclear if all prespecified outcomes were reported, raising potential risk of selective reporting
	5.2. Is the numerical result being assessed likely to have been selected, on the basis of the results, from multiple eligible outcome measurements (e.g. scales, definitions, time points) within the outcome domain?	PN/N	
	5.3. Is the numerical result being assessed likely to have been selected, on the basis of the results, from multiple eligible analyses of the data?	PN/N	

		SOME CONCERNS	
Overall Judgment		LOW	

TITLE: 2- Role of erector spinae plane block versus paravertebral block in pain control after modified radical mastectomy. A prospective randomised trial[18]			
Domain	Subdomain	Risk of Bias	Justification
1.Bias arising from the randomization process	1.1. Was the allocation sequence random?	Y/PY	Patients were randomized (1:1 ratio) into two group
	1.2. Was the allocation sequence concealed until participants were enrolled and assigned to interventions?	NI	
	1.3. Did baseline differences between intervention groups suggest a problem with the randomization process?	PN/N	Two equal groups with no statistically significant differences in age, weight and duration of surgery
	SUBRISK	SOME CONCERNS	
2. Bias due to deviations from intended interventions	2.1. Were participants aware of their assigned intervention during the trial?	PN/N	
	2.2. Were carers and people delivering the interventions aware of participants' assigned intervention during the trial?	PN/N	
	2.3. If Y/PY/NI to 2.1 or 2.2: Were there deviations from the intended intervention that arose because of the trial context?	PN/N	
	2.4 If Y/PY to 2.3: Were these deviations likely to have affected the outcome?	NI	
	2.5. If Y/PY/NI to 2.4: Were these deviations from intended intervention balanced between groups?	NI	
	2.6 Was an appropriate analysis used to estimate the effect of assignment to intervention?	Y/PY	
	2.7 If N/PN/NI to 2.6: Was there potential for a substantial impact (to the result) of the failure to analyse participants in the group to which they were randomized?		
	SUBRISK	LOW	
Bias due to missing outcome data	3.1 Were data for this outcome available for all, or nearly all, participants randomized?	Y/PY	70 patients were randomized and analyzed; no dropouts or exclusions reported; no block failures.
	3.2 If N/PN/NI to 3.1: Is there evidence that the result was not biased by missing outcome data?		
	3.3 If N/PN to 3.2: Could missingness in the outcome depend on its true value?		
	3.4 If Y/PY/NI to 3.3: Is it likely that missingness in the outcome depended on its true value?		
	SUBRISK	LOW	
Bias in measurement of the outcome	4.1 Was the method of measuring the outcome inappropriate?	Y/PY	Primary outcome was 24-h morphine consumption (objective); VAS scores were assessed by a blinded assessor at predefined time points.
	4.2 Could measurement or ascertainment of the outcome have differed between intervention groups?	PN/N	
	4.3 If N/PN/NI to 4.1 and 4.2: Were outcome assessors aware of the intervention received by study participants?	PN/N	
	4.4 If Y/PY/NI to 4.3: Could assessment of the outcome have been influenced by knowledge of intervention received?	NI	
	4.5 If Y/PY/NI to 4.4: Is it likely that assessment of the outcome was influenced by knowledge of intervention received?	NI	
	SUBRISK	LOW	
Bias in selection of the reported result	5.1 Were the data that produced this result analysed in accordance with a pre-specified analysis plan that was finalized before unblinded outcome data were available for analysis?	Y/PY	No trial registration available; unclear if all prespecified outcomes were reported, raising potential risk of selective reporting
	5.2. Is the numerical result being assessed likely to have been selected, on the basis of the results, from multiple eligible outcome measurements (e.g. scales, definitions, time points) within the outcome domain?	PN/N	

	5.3. Is the numerical result being assessed likely to have been selected, on the basis of the results, from multiple eligible analyses of the data?	PN/N	
	SUBRISK	SOME CONCERNS	
Overall Judgment		SOME CONCERNS	

**TİTLE: 3- Erector spinae versus paravertebral plane blocks in modified radical mastectomy: Randomised comparative study of the technique success rate among novice anaesthesiologists[19]**			
Domain	Subdomain	Risk of Bias	Justification
1.Bias arising from the randomization process	1.1. Was the allocation sequence random?	Y/PY	Patients were randomly allocated into two groups using computer-generated random numbers.
	1.2. Was the allocation sequence concealed until participants were enrolled and assigned to interventions?	Y/PY	The group allocation numbers were concealed in sealed opaque envelopes that were opened after enrolment of the patients
	1.3. Did baseline differences between intervention groups suggest a problem with the randomization process?	PN/N	Baseline characteristics were comparable.
	SUBRISK	LOW	
2. Bias due to deviations from intended interventions	2.1. Were participants aware of their assigned intervention during the trial?	NI	The study was described as prospective, randomized, supervised.
	2.2. Were carers and people delivering the interventions aware of participants' assigned intervention during the trial?	NI	
	2.3. If Y/PY/NI to 2.1 or 2.2: Were there deviations from the intended intervention that arose because of the trial context?		
	2.4 If Y/PY to 2.3: Were these deviations likely to have affected the outcome?		
	2.5. If Y/PY/NI to 2.4: Were these deviations from intended intervention balanced between groups?		
	2.6 Was an appropriate analysis used to estimate the effect of assignment to intervention?		
	2.7 If N/PN/NI to 2.6: Was there potential for a substantial impact (to the result) of the failure to analyse participants in the group to which they were randomized?		
	SUBRISK	SOME CONCERNS	
Bias due to missing outcome data	3.1 Were data for this outcome available for all, or nearly all, participants randomized?	Y/PY	No loss to follow-up reported; all randomized patients analyzed per group.
	3.2 If N/PN/NI to 3.1: Is there evidence that the result was not biased by missing outcome data?		
	3.3 If N/PN to 3.2: Could missingness in the outcome depend on its true value?		
	3.4 If Y/PY/NI to 3.3: Is it likely that missingness in the outcome depended on its true value?		
	SUBRISK	LOW	
Bias in measurement of the outcome	4.1 Was the method of measuring the outcome inappropriate?	PN/N	Primary outcome = success rate of block within 10 min, judged by consultants; postoperative VAS and morphine use assessed by a blinded investigator.
	4.2 Could measurement or ascertainment of the outcome have differed between intervention groups?	PN/N	
	4.3 If N/PN/NI to 4.1 and 4.2: Were outcome assessors aware of the intervention received by study participants?	PN/N	
	4.4 If Y/PY/NI to 4.3: Could assessment of the outcome have been influenced by knowledge of intervention received?		
	4.5 If Y/PY/NI to 4.4: Is it likely that assessment of the outcome was influenced by knowledge of intervention received?		
	SUBRISK	LOW	

Bias in selection of the reported result	5.1. Were the data that produced this result analysed in accordance with a pre-specified analysis plan that was finalized before unblinded outcome data were available for analysis?	Y/PY	A sample size of 90 patients (45 blocks per group) was found to achieve 81% power to detect a difference of 30% success rate between the ESPB technique compared to the paravertebral technique at significance level of 5%.
	5.2. Is the numerical result being assessed likely to have been selected, on the basis of the results, from multiple eligible outcome measurements (e.g. scales, definitions, time points) within the outcome domain?	PN/N	
	5.3. Is the numerical result being assessed likely to have been selected, on the basis of the results, from multiple eligible analyses of the data?	PN/N	
	SUBRISK	LOW	
Overall Judgment		SOME CONCERNS	

**TİTLE-4:Erector spinae plane versus paravertebral nerve blocks for postoperative analgesia after breast surgery: a randomized clinical trial[20]**			
Domain	Subdomain	Risk of Bias	Justification
1.Bias arising from the randomization process	1.1. Was the allocation sequence random?	Y/PY	Participants were randomized using a computer-generated list and opaque, sealed envelopes to one of two treatment groups stratified for unilateral versus bilateral surgery
	1.2. Was the allocation sequence concealed until participants were enrolled and assigned to interventions?	Y/PY	
	1.3. Did baseline differences between intervention groups suggest a problem with the randomization process?	PN/N	
	SUBRISK	LOW	
2. Bias due to deviations from intended interventions	2.1. Were participants aware of their assigned intervention during the trial?	PN/N	Participants were subject-blinded; all blocks done by fellowship-trained attendings/fellows under ultrasound guidance
	2.2. Were carers and people delivering the interventions aware of participants' assigned intervention during the trial?	PN/N	Personnel all masked to treatment group allocation. Data from the operating and recovery rooms was therefore collected by masked observers. We chose to limit our masked pain and opioid assessments to the recovery room which included the dual primary outcome measures.
	2.3. If Y/PY/NI to 2.1 or 2.2: Were there deviations from the intended intervention that arose because of the trial context?		
	2.4 If Y/PY to 2.3: Were these deviations likely to have affected the outcome?		
	2.5. If Y/PY/NI to 2.4: Were these deviations from intended intervention balanced between groups?		
	2.6 Was an appropriate analysis used to estimate the effect of assignment to intervention?	Y/PY	Two-sample Mann-Whitney test or Chi-squared test for two proportions, as appropriate. Noninferiority: Wilcoxon-Mann-Whitney test.
	2.7 If N/PN/NI to 2.6: Was there potential for a substantial impact (to the result) of the failure to analyse participants in the group to which they were randomized?		
	SUBRISK	LOW	
Bias due to missing outcome data	3.1 Were data for this outcome available for all, or nearly all, participants randomized?	Y/PY	100 patients randomized (50 ESPB, 50 PVB). All completed follow-up and were analyzed; no losses reported.
	3.2 If N/PN/NI to 3.1: Is there evidence that the result was not biased by missing outcome data?		
	3.3 If N/PN to 3.2: Could missingness in the outcome depend on its true value?		
	3.4 If Y/PY/NI to 3.3: Is it likely that missingness in the outcome depended on its true value?		
	SUBRISK	LOW	

Bias in measurement of the outcome	4.1 Was the method of measuring the outcome inappropriate?	PN/N	Primary outcomes: PACU pain scores (NRS) and opioid use. PACU outcomes recorded by nurses blinded to allocation; follow-up also blinded.
	4.2 Could measurement or ascertainment of the outcome have differed between intervention groups?	PN/N	
	4.3 If N/PN/NI to 4.1 and 4.2: Were outcome assessors aware of the intervention received by study participants?	PN/N	
	4.4 If Y/PY/NI to 4.3: Could assessment of the outcome have been influenced by knowledge of intervention received?		
	4.5 If Y/PY/NI to 4.4: Is it likely that assessment of the outcome was influenced by knowledge of intervention received?		
	SUBRISK	LOW	
Bias in selection of the reported result	5.1. Were the data that produced this result analysed in accordance with a pre-specified analysis plan that was finalized before unblinded outcome data were available for analysis?	Y/PY	Primary and secondary outcomes were reported as planned; analyses consistent with protocol.
	5.2. Is the numerical result being assessed likely to have been selected, on the basis of the results, from multiple eligible outcome measurements (e.g. scales, definitions, time points) within the outcome domain?		
	5.3. Is the numerical result being assessed likely to have been selected, on the basis of the results, from multiple eligible analyses of the data?		
	SUBRISK	LOW	
Overall Judgment		LOW	

TITLE-5: Erector spinae plane block and thoracic paravertebral block for breast surgery compared to IV-morphine: A randomized controlled trial[21]

Domain	Subdomain	Risk of Bias	Justification
1.Bias arising from the randomization process	1.1. Was the allocation sequence random?	Y/PY	Randomized, parallel-group, double-blind
	1.2. Was the allocation sequence concealed until participants were enrolled and assigned to interventions?	Y/PY	Randomization of participants was done by computer-generated random number table utilizing sequentially numbered sealed opaque envelopes for concealment.
	1.3. Did baseline differences between intervention groups suggest a problem with the randomization process?	PN/N	Patients were comparable among the groups with respect to age, height, ASA physical status, and duration of surgery.
	SUBRISK	LOW	
2. Bias due to deviations from intended interventions	2.1. Were participants aware of their assigned intervention during the trial?	PN/N	The patient and the assessor were blinded toward group allocation, pain modality treatment provided, and study outcome
	2.2. Were carers and people delivering the interventions aware of participants' assigned intervention during the trial?	PN/N	The patient and the assessor were blinded toward group allocation, pain modality treatment provided, and study outcome.
	2.3. If Y/PY/NI to 2.1 or 2.2: Were there deviations from the intended intervention that arose because of the trial context?		
	2.4. If Y/PY to 2.3: Were these deviations likely to have affected the outcome?		
	2.5. If Y/PY/NI to 2.4: Were these deviations from intended intervention balanced between groups?		
	2.6. Was an appropriate analysis used to estimate the effect of assignment to intervention?	Y/PY	
	2.7. If N/PN/NI to 2.6: Was there potential for a substantial impact (to the result) of the failure to analyse participants in the group to which they were randomized?		

	SUBRISK	LOW	
Bias due to missing outcome data	3.1 Were data for this outcome available for all, or nearly all, participants randomized?	Y/PY	80 patients randomized; all completed the study and were included in analysis. No dropouts, exclusions, or block failures reported.
	3.2 If N/PN/NI to 3.1: Is there evidence that the result was not biased by missing outcome data?		
	3.3 If N/PN to 3.2: Could missingness in the outcome depend on its true value?		
	3.4 If Y/PY/NI to 3.3: Is it likely that missingness in the outcome depended on its true value?		
	SUBRISK	LOW	
Bias in measurement of the outcome	4.1 Was the method of measuring the outcome inappropriate?	Y/PY	
	4.2 Could measurement or ascertainment of the outcome have differed between intervention groups?	PN/N	
	4.3 If N/PN/NI to 4.1 and 4.2: Were outcome assessors aware of the intervention received by study participants?	PN/N	
	4.4 If Y/PY/NI to 4.3: Could assessment of the outcome have been influenced by knowledge of intervention received?		
	4.5 If Y/PY/NI to 4.4: Is it likely that assessment of the outcome was influenced by knowledge of intervention received?		
	SUBRISK	LOW	
Bias in selection of the reported result	5.1. Were the data that produced this result analysed in accordance with a pre-specified analysis plan that was finalized before unblinded outcome data were available for analysis?	Y/PY	Outcomes and analyses consistent with protocol; selective reporting unlikely.
	5.2. Is the numerical result being assessed likely to have been selected, on the basis of the results, from multiple eligible outcome measurements (e.g. scales, definitions, time points) within the outcome domain?	PN/N	
	5.3. Is the numerical result being assessed likely to have been selected, on the basis of the results, from multiple eligible analyses of the data?	PN/N	
	SUBRISK	LOW	
Overall Judgment		LOW	

**TITLE-6: Comparison of paravertebral block vs erector spinae plane block in breast surgery patients[22]**			
Domain	Subdomain	Risk of Bias	Justification
1.Bias arising from the randomization process	1.1. Was the allocation sequence random?	NI	The study mentions patients were randomized into two groups (n=30 each),
	1.2. Was the allocation sequence concealed until participants were enrolled and assigned to interventions?	NI	Allocation concealment was ensured as the numbers were put into sealed opaque envelopes and drawn up by the anesthetist scheduled to administer the block.
	1.3. Did baseline differences between intervention groups suggest a problem with the randomization process?		Among the two groups, there was no difference in age, body mass index, education, ASA status, location and volume of breast tumour excised or the duration of surgery
	SUBRISK	SOME CONCERNS	
2. Bias due to deviations from intended interventions	2.1. Were participants aware of their assigned intervention during the trial?	NI	
	2.2. Were carers and people delivering the interventions aware of participants' assigned intervention during the trial?	Y	Single anesthetist administered both GA and block, unblinded; participants and clinicians were not blinded, increasing risk of performance bias
	2.3. If Y/PY/NI to 2.1 or 2.2: Were there deviations from the intended intervention that arose because of the trial context?	NI	
	2.4 If Y/PY to 2.3: Were these deviations likely to have affected the outcome?	NI	
	2.5. If Y/PY/NI to 2.4: Were these deviations from intended intervention balanced between groups?		

	2.6 Was an appropriate analysis used to estimate the effect of assignment to intervention?	NI	
	2.7 If N/PN/NI to 2.6: Was there potential for a substantial impact (to the result) of the failure to analyse participants in the group to which they were randomized?		
	SUBRISK	HIGH	
Bias due to missing outcome data	3.1 Were data for this outcome available for all, or nearly all, participants randomized?	Y/PY	No losses to follow-up reported; outcomes available for all randomized patient
	3.2 If N/PN/NI to 3.1: Is there evidence that the result was not biased by missing outcome data?		
	3.3 If N/PN to 3.2: Could missingness in the outcome depend on its true value?		
	3.4 If Y/PY/NI to 3.3: Is it likely that missingness in the outcome depended on its true value?		
	SUBRISK	LOW	
Bias in measurement of the outcome	4.1 Was the method of measuring the outcome inappropriate?	Y/PY	VAS
	4.2 Could measurement or ascertainment of the outcome have differed between intervention groups?	NI	Outcomes could be influenced by lack of blinding of outcome assessors and patients.
	4.3 If N/PN/NI to 4.1 and 4.2: Were outcome assessors aware of the intervention received by study participants?	NI	
	4.4 If Y/PY/NI to 4.3: Could assessment of the outcome have been influenced by knowledge of intervention received?	NI	
	4.5 If Y/PY/NI to 4.4: Is it likely that assessment of the outcome was influenced by knowledge of intervention received?	NI	
	SUBRISK	HIGH	
Bias in selection of the reported result	5.1. Were the data that produced this result analysed in accordance with a pre-specified analysis plan that was finalized before unblinded outcome data were available for analysis?	PN/N	No pre-registered protocol or trial registration available. Selective reporting cannot be excluded.
	5.2. Is the numerical result being assessed likely to have been selected, on the basis of the results, from multiple eligible outcome measurements (e.g. scales, definitions, time points) within the outcome domain?		
	5.3. Is the numerical result being assessed likely to have been selected, on the basis of the results, from multiple eligible analyses of the data?		
	SUBRISK	LOW	
Overall Judgment		HIGH	

TITLE-7: **The comparison of the efficacy of ultrasound-guided paravertebral block versus erector spinae plane block for postoperative analgesia in modified radical mastectomy: A randomized controlled trial**[23]			
Domain	Subdomain	Risk of Bias	Justification
1.Bias arising from the randomization process	1.1. Was the allocation sequence random?	Y/PY	The patients were randomly divided into two equal groups by computer-generated random number table.
	1.2. Was the allocation sequence concealed until participants were enrolled and assigned to interventions?	Y/PY	The group allocation numbers were concealed in sealed opaque envelopes that was opened by an anesthesiologist not involved in the study.
	1.3. Did baseline differences between intervention groups suggest a problem with the randomization process?	PN/N	The groups were comparable with respect to age, weight, ASA physical status, and the duration of surgery
	SUBRISK	LOW	
2. Bias due to deviations from intended interventions	2.1. Were participants aware of their assigned intervention during the trial?	NI	
	2.2. Were carers and people delivering the interventions aware of participants' assigned intervention during the trial?	PN/N	The observer who collected perioperative data was blinded to the technique of analgesia used.
	2.3. If Y/PY/NI to 2.1 or 2.2: Were there deviations from the intended intervention that arose because of the trial context?		

	2.4 If Y/PY to 2.3: Were these deviations likely to have affected the outcome?		
	2.5. If Y/PY/NI to 2.4: Were these deviations from intended intervention balanced between groups?		
	2.6 Was an appropriate analysis used to estimate the effect of assignment to intervention?	Y/PY	
	2.7 If N/PN/NI to 2.6: Was there potential for a substantial impact (to the result) of the failure to analyse participants in the group to which they were randomized?		
	SUBRISK	LOW	
Bias due to missing outcome data	3.1 Were data for this outcome available for all, or nearly all, participants randomized?	Y/PY	All randomized patients (n=60) completed the study and were included in the analysis; no dropouts reported.
	3.2 If N/PN/NI to 3.1: Is there evidence that the result was not biased by missing outcome data?		
	3.3 If N/PN to 3.2: Could missingness in the outcome depend on its true value?		
	3.4 If Y/PY/NI to 3.3: Is it likely that missingness in the outcome depended on its true value?		
	SUBRISK	LOW	
Bias in measurement of the outcome	4.1 Was the method of measuring the outcome inappropriate?	Y/PY	NRS
	4.2 Could measurement or ascertainment of the outcome have differed between intervention groups?	PN/N	
	4.3 If N/PN/NI to 4.1 and 4.2: Were outcome assessors aware of the intervention received by study participants?	PN/N	
	4.4 If Y/PY/NI to 4.3: Could assessment of the outcome have been influenced by knowledge of intervention received?		
	4.5 If Y/PY/NI to 4.4: Is it likely that assessment of the outcome was influenced by knowledge of intervention received?		
	SUBRISK	LOW	
Bias in selection of the reported result	5.1. Were the data that produced this result analysed in accordance with a pre-specified analysis plan that was finalized before unblinded outcome data were available for analysis?	Y/PY	The sample size was calculated based on a study by Kapil Gupta et al.
	5.2. Is the numerical result being assessed likely to have been selected, on the basis of the results, from multiple eligible outcome measurements (e.g. scales, definitions, time points) within the outcome domain?	PN/N	
	5.3. Is the numerical result being assessed likely to have been selected, on the basis of the results, from multiple eligible analyses of the data?	PN/N	
	SUBRISK	LOW	
Overall Judgment		LOW	

**Title 8- Comparison between erector spinae plane block and paravertebral block regarding postoperative analgesic consumption Following breast surgery: a randomized controlled study[24]**	C o m p a ri s o n b

	etweenerectorspinaeplaneblockandparavertebralblo

	ck regarding postoperative analgesic consumption follow

ing breast surgery: a randomized controlled study

Domain	Subdomain	Risk of Bias	Justification
1.Bias arising from the randomization process	1.1. Was the allocation sequence random?	Y/PY	Randomized, single blind study: randomly allocated by computer generated random numbers into two groups
	1.2. Was the allocation sequence concealed until participants were enrolled and assigned to interventions?	NI	
	1.3. Did baseline differences between intervention groups suggest a problem with the randomization process?	PN/N	Demographic profile and baseline hemodynamic parameters were equivalent in both groups

	SUBRISK	LOW	
2. Bias due to deviations from intended interventions	2.1. Were participants aware of their assigned intervention during the trial?	PN/N	Blocks performed by an anesthesiologist not involved in assessments; patients and outcome assessors blinded. Standardized anesthetic and analgesic protocols were used.
	2.2. Were carers and people delivering the interventions aware of participants' assigned intervention during the trial?	Y/PY	
	2.3. If Y/PY/NI to 2.1 or 2.2: Were there deviations from the intended intervention that arose because of the trial context?	PN/N	
	2.4 If Y/PY to 2.3: Were these deviations likely to have affected the outcome?	PN/N	
	2.5. If Y/PY/NI to 2.4: Were these deviations from intended intervention balanced between groups?		
	2.6 Was an appropriate analysis used to estimate the effect of assignment to intervention?	Y/PY	
	2.7 If N/PN/NI to 2.6: Was there potential for a substantial impact (to the result) of the failure to analyse participants in the group to which they were randomized?		
	SUBRISK	LOW	
Bias due to missing outcome data	3.1 Were data for this outcome available for all, or nearly all, participants randomized?	Y/PY	
	3.2 If N/PN/NI to 3.1: Is there evidence that the result was not biased by missing outcome data?		
	3.3 If N/PN to 3.2: Could missingness in the outcome depend on its true value?		
	3.4 If Y/PY/NI to 3.3: Is it likely that missingness in the outcome depended on its true value?		
	SUBRISK	LOW	
Bias in measurement of the outcome	4.1 Was the method of measuring the outcome inappropriate?	Y/PY	VAS, Time to first rescue analgesia
	4.2 Could measurement or ascertainment of the outcome have differed between intervention groups?	PN/N	
	4.3 If N/PN/NI to 4.1 and 4.2: Were outcome assessors aware of the intervention received by study participants?	PN/N	Authors involved in data collection were blinded to the block
	4.4 If Y/PY/NI to 4.3: Could assessment of the outcome have been influenced by knowledge of intervention received?		
	4.5 If Y/PY/NI to 4.4: Is it likely that assessment of the outcome was influenced by knowledge of intervention received?		
	SUBRISK	LOW	
Bias in selection of the reported result	5.1. Were the data that produced this result analysed in accordance with a pre-specified analysis plan that was finalized before unblinded outcome data were available for analysis?	Y/PY	A sample size of 14 patients per group was required to detect 9.32 mg differences between the means of 24 h postoperative morphine consumption between the ESPB and control groups at a standard deviation of 7.44 with 90% power and a 5% level of significance.
	5.2. Is the numerical result being assessed likely to have been selected, on the basis of the results, from multiple eligible outcome measurements (e.g. scales, definitions, time points) within the outcome domain?	PN/N	
	5.3. Is the numerical result being assessed likely to have been selected, on the basis of the results, from multiple eligible analyses of the data?	PN/N	
	SUBRISK	LOW	
Overall Judgment		LOW	

TITLE-9: Paravertebral block versus erector spinae plane block for analgesia in modified radical mastectomy: a randomized, prospective, double-blind study[25]

Domain	Subdomain	Risk of Bias	Justification
1.Bias arising from the randomization process	1.1. Was the allocation sequence random?	Y/PY	The patients were randomly allocated by a computer-generated random number table to three groups
	1.2. Was the allocation sequence concealed until participants were enrolled and assigned to interventions?	Y/PY	Allocation concealment was ensured by having the random group assignment enclosed in a sealed opaque envelope.
	1.3. Did baseline differences between intervention groups suggest a problem with the randomization process?	PN/N	
	SUBRISK	LOW	
2. Bias due to deviations from intended interventions	2.1. Were participants aware of their assigned intervention during the trial?	PN/N	Patients and assessors were blinded.
	2.2. Were carers and people delivering the interventions aware of participants' assigned intervention during the trial?	PN/N	
	2.3. If Y/PY/NI to 2.1 or 2.2: Were there deviations from the intended intervention that arose because of the trial context?		
	2.4 If Y/PY to 2.3: Were these deviations likely to have affected the outcome?		
	2.5. If Y/PY/NI to 2.4: Were these deviations from intended intervention balanced between groups?		
	2.6 Was an appropriate analysis used to estimate the effect of assignment to intervention?	Y/PY	
	2.7 If N/PN/NI to 2.6: Was there potential for a substantial impact (to the result) of the failure to analyse participants in the group to which they were randomized?		
	SUBRISK	LOW	
Bias due to missing outcome data	3.1 Were data for this outcome available for all, or nearly all, participants randomized?	Y/PY	All randomized patients were analyzed; no losses to follow-up or exclusions reported.
	3.2 If N/PN/NI to 3.1: Is there evidence that the result was not biased by missing outcome data?		
	3.3 If N/PN to 3.2: Could missingness in the outcome depend on its true value?		
	3.4 If Y/PY/NI to 3.3: Is it likely that missingness in the outcome depended on its true value?		
	SUBRISK	LOW	
Bias in measurement of the outcome	4.1 Was the method of measuring the outcome inappropriate?	Y/PY	
	4.2 Could measurement or ascertainment of the outcome have differed between intervention groups?	PN/N	
	4.3 If N/PN/NI to 4.1 and 4.2: Were outcome assessors aware of the intervention received by study participants?	PN/N	
	4.4 If Y/PY/NI to 4.3: Could assessment of the outcome have been influenced by knowledge of intervention received?		
	4.5 If Y/PY/NI to 4.4: Is it likely that assessment of the outcome was influenced by knowledge of intervention received?		
	SUBRISK	LOW	
Bias in selection of the reported result	5.1. Were the data that produced this result analysed in accordance with a pre-specified analysis plan that was finalized before unblinded outcome data were available for analysis?	Y/PY	Sample size was estimated using pain scores as the primary variable
	5.2. Is the numerical result being assessed likely to have been selected, on the basis of the results, from multiple eligible outcome measurements (e.g. scales, definitions, time points) within the outcome domain?	PN/N	
	5.3. Is the numerical result being assessed likely to have been selected, on the basis of the results, from multiple eligible analyses of the data?	PN/N	
	SUBRISK	LOW	
Overall Judgment		LOW	

TITLE-10: A randomized double-blind controlled study comparing erector spinae plane block and thoracic paravertebral block for postoperative analgesia after breast surgery[26]			
Domain	Subdomain	Risk of Bias	Justification
1.Bias arising from the randomization process	1.1. Was the allocation sequence random?	Y/PY	Before surgery patients were randomly allocated according to the computer generated sequence into two equal groups
	1.2. Was the allocation sequence concealed until participants were enrolled and assigned to interventions?	Y/PY	
	1.3. Did baseline differences between intervention groups suggest a problem with the randomization process?	PN/N	The two groups were comparable with respect to age, sex, BMI, ASA physical status, surgical procedures and surgical time
	SUBRISK	LOW	
2. Bias due to deviations from intended interventions	2.1. Were participants aware of their assigned intervention during the trial?	PN/N	Blocks performed by anesthesiologists not involved in assessments. Patients, surgeons, and assessors were blinded. Standardized anesthesia and postoperative analgesia protocols applied.
	2.2. Were carers and people delivering the interventions aware of participants' assigned intervention during the trial?	PN/N	
	2.3. If Y/PY/NI to 2.1 or 2.2: Were there deviations from the intended intervention that arose because of the trial context?		
	2.4 If Y/PY to 2.3: Were these deviations likely to have affected the outcome?		
	2.5. If Y/PY/NI to 2.4: Were these deviations from intended intervention balanced between groups?		
	2.6 Was an appropriate analysis used to estimate the effect of assignment to intervention?	Y/PY	
	2.7 If N/PN/NI to 2.6: Was there potential for a substantial impact (to the result) of the failure to analyse participants in the group to which they were randomized?		
	SUBRISK	LOW	
Bias due to missing outcome data	3.1 Were data for this outcome available for all, or nearly all, participants randomized?	Y/PY	All randomized patients (n=70) completed the study and were included in analysis; no dropouts reported.
	3.2 If N/PN/NI to 3.1: Is there evidence that the result was not biased by missing outcome data?		
	3.3 If N/PN to 3.2: Could missingness in the outcome depend on its true value?		
	3.4 If Y/PY/NI to 3.3: Is it likely that missingness in the outcome depended on its true value?		
	SUBRISK	LOW	
Bias in measurement of the outcome	4.1 Was the method of measuring the outcome inappropriate?	Y/PY	
	4.2 Could measurement or ascertainment of the outcome have differed between intervention groups?	PN/N	
	4.3 If N/PN/NI to 4.1 and 4.2: Were outcome assessors aware of the intervention received by study participants?	PN/N	
	4.4 If Y/PY/NI to 4.3: Could assessment of the outcome have been influenced by knowledge of intervention received?		
	4.5 If Y/PY/NI to 4.4: Is it likely that assessment of the outcome was influenced by knowledge of intervention received?		
	SUBRISK	LOW	
Bias in selection of the reported result	5.1. Were the data that produced this result analysed in accordance with a pre-specified analysis plan that was finalized before unblinded outcome data were available for analysis?	Y/PY	Sample size was calculated using the program on the web-site https://clincalc.com/stats/samplesize.aspx). A pilot study (n = 10) conducted at our institution revealed that patients undergoing PVB for unilateral mastectomy reported a mean 24-h morphine consumption of 6.5 mg, with a stan-dard deviation of 1.5 mg (unpublished data).

	5.2. Is the numerical result being assessed likely to have been selected, on the basis of the results, from multiple eligible outcome measurements (e.g. scales, definitions, time points) within the outcome domain?	PN/N	
	5.3. Is the numerical result being assessed likely to have been selected, on the basis of the results, from multiple eligible analyses of the data?	PN/N	
	SUBRISK	LOW	
Overall Judgment		LOW	

TİTLE: 11- Role Of Presurgical Erector Spinae Plane Block Versus Paravertebral Block In Pain Control And Hemodynamic Stability After Modified Radical Mastectomy-A ProspectiveRandomized Trial [27]			
Domain	Subdomain	Risk of Bias	Justification
1.Bias arising from the randomization process	1.1. Was the allocation sequence random?	Y/PY	
	1.2. Was the allocation sequence concealed until participants were enrolled and assigned to interventions?	Y/PY	
	1.3. Did baseline differences between intervention groups suggest a problem with the randomization process?	PN/N	
	SUBRISK	LOW	
2. Bias due to deviations from intended interventions	2.1. Were participants aware of their assigned intervention during the trial?	Y/PY	No mention of blinding for patients, anesthesiologists, or outcome assessors. Interventions delivered by the same anesthesia team who knew the allocation.
	2.2. Were carers and people delivering the interventions aware of participants' assigned intervention during the trial?	NI	.
	2.3. If Y/PY/NI to 2.1 or 2.2: Were there deviations from the intended intervention that arose because of the trial context?		
	2.4 If Y/PY to 2.3: Were these deviations likely to have affected the outcome?		
	2.5. If Y/PY/NI to 2.4: Were these deviations from intended intervention balanced between groups?		
	2.6 Was an appropriate analysis used to estimate the effect of assignment to intervention?		
	2.7 If N/PN/NI to 2.6: Was there potential for a substantial impact (to the result) of the failure to analyse participants in the group to which they were randomized?		
		SOME CONCERNS	
Bias due to missing outcome data	3.1 Were data for this outcome available for all, or nearly all, participants randomized?	Y/PY	
	3.2 If N/PN/NI to 3.1: Is there evidence that the result was not biased by missing outcome data?		
	3.3 If N/PN to 3.2: Could missingness in the outcome depend on its true value?		
	3.4 If Y/PY/NI to 3.3: Is it likely that missingness in the outcome depended on its true value?		
		LOW	
Bias in measurement of the outcome	4.1 Was the method of measuring the outcome inappropriate?	PN/N	Pain scores (VAS) and opioid consumption assessed without blinding; outcomes could have been influenced by knowledge of the intervention.
	4.2 Could measurement or ascertainment of the outcome have differed between intervention groups?	NI	
	4.3 If N/PN/NI to 4.1 and 4.2: Were outcome assessors aware of the intervention received by study participants?	NI	
	4.4 If Y/PY/NI to 4.3: Could assessment of the outcome have been influenced by knowledge of intervention received?	NI	

	4.5 If Y/PY/NI to 4.4: Is it likely that assessment of the outcome was influenced by knowledge of intervention received?	NI	
		HIGH	
Bias in selection of the reported result	5.1. Were the data that produced this result analysed in accordance with a pre-specified analysis plan that was finalized before unblinded outcome data were available for analysis?	Y/PY	No trial registration or published protocol found. Selective outcome reporting cannot be ruled out.
	5.2. Is the numerical result being assessed likely to have been selected, on the basis of the results, from multiple eligible outcome measurements (e.g. scales, definitions, time points) within the outcome domain?	PN/N	
	5.3. Is the numerical result being assessed likely to have been selected, on the basis of the results, from multiple eligible analyses of the data?	PN/N	
		SOME CONCERNS	
Overall Judgment		HIGH	

TITLE-12: A comparative study between ultrasound-guided thoracic paravertebral block, pectoral nerves block, and erector spinae block for pain management in cancer breast surgeries. A randomized controlled study [28]

Domain	Subdomain	Risk of Bias	Justification
1.Bias arising from the randomization process	1.1. Was the allocation sequence random?	Y/PY	Randomized, single blind study: randomly allocated by computer generated random numbers into two groups
	1.2. Was the allocation sequence concealed until participants were enrolled and assigned to interventions?	Y/PY	
	1.3. Did baseline differences between intervention groups suggest a problem with the randomization process?	PN/N	Demographic profile and baseline hemodynamic parameters were equivalent in both groups
	SUBRISK	LOW	
2. Bias due to deviations from intended interventions	2.1. Were participants aware of their assigned intervention during the trial?	PN/N	Blocks performed by an anesthesiologist not involved in assessments; patients and outcome assessors blinded. Standardized anesthetic and analgesic protocols were used.
	2.2. Were carers and people delivering the interventions aware of participants' assigned intervention during the trial?	Y/PY	
	2.3. If Y/PY/NI to 2.1 or 2.2: Were there deviations from the intended intervention that arose because of the trial context?	PN/N	
	2.4 If Y/PY to 2.3: Were these deviations likely to have affected the outcome?	PN/N	
	2.5. If Y/PY/NI to 2.4: Were these deviations from intended intervention balanced between groups?		
	2.6 Was an appropriate analysis used to estimate the effect of assignment to intervention?	Y/PY	
	2.7 If N/PN/NI to 2.6: Was there potential for a substantial impact (to the result) of the failure to analyse participants in the group to which they were randomized?		
	SUBRISK	LOW	
Bias due to missing outcome data	3.1 Were data for this outcome available for all, or nearly all, participants randomized?	Y/PY	
	3.2 If N/PN/NI to 3.1: Is there evidence that the result was not biased by missing outcome data?		
	3.3 If N/PN to 3.2: Could missingness in the outcome depend on its true value?		
	3.4 If Y/PY/NI to 3.3: Is it likely that missingness in the outcome depended on its true value?		
	SUBRISK	LOW	
Bias in measurement of the outcome	4.1 Was the method of measuring the outcome inappropriate?	Y/PY	VAS, Time to first rescue analgesia
	4.2 Could measurement or ascertainment of the outcome have differed between intervention groups?	PN/N	
	4.3 If N/PN/NI to 4.1 and 4.2: Were outcome assessors aware of the intervention received by study participants?	PN/N	Authors involved in data collection were blinded to the block

	4.4 If Y/PY/NI to 4.3: Could assessment of the outcome have been influenced by knowledge of intervention received?		
	4.5 If Y/PY/NI to 4.4: Is it likely that assessment of the outcome was influenced by knowledge of intervention received?		
	SUBRISK	LOW	
Bias in selection of the reported result	5.1. Were the data that produced this result analysed in accordance with a pre-specified analysis plan that was finalized before unblinded outcome data were available for analysis?	Y/PY	A sample size of 14 patients per group was required to detect 9.32 mg differences between the means of 24 h postoperative morphine consumption between the ESPB and control groups at a standard deviation of 7.44 with 90% power and a 5% level of significance.
	5.2. Is the numerical result being assessed likely to have been selected, on the basis of the results, from multiple eligible outcome measurements (e.g. scales, definitions, time points) within the outcome domain?	PN/N	
	5.3. Is the numerical result being assessed likely to have been selected, on the basis of the results, from multiple eligible analyses of the data?	PN/N	
	SUBRISK	LOW	
Overall Judgment		LOW	

**TİTLE: 13- Comparative Study between Ultrasound Guided Erector Spinae Plane Block versus Paravrtebral Block for Postoperative Pain Relief in Patients Undergoing Unilateral Modified Radical Mastectomy[29]**			
Domain	Subdomain	Risk of Bias	Justification
1.Bias arising from the randomization process	1.1. Was the allocation sequence random?	Y/PY	
	1.2. Was the allocation sequence concealed until participants were enrolled and assigned to interventions?	NI	
	1.3. Did baseline differences between intervention groups suggest a problem with the randomization process?	N	Patients balanced for demographics
	SUBRISK	LOW	
2. Bias due to deviations from intended interventions	2.1. Were participants aware of their assigned intervention during the trial?	Y/PY	
	2.2. Were carers and people delivering the interventions aware of participants' assigned intervention during the trial?	Y/PY	Study described as randomized and double-blind; however, anesthesiologists performing the block were aware of group allocation (cannot be blinded). Patients and assessors were blinded.
	2.3. If Y/PY/NI to 2.1 or 2.2: Were there deviations from the intended intervention that arose because of the trial context?	Y/PY	
	2.4 If Y/PY to 2.3: Were these deviations likely to have affected the outcome?		
	2.5. If Y/PY/NI to 2.4: Were these deviations from intended intervention balanced between groups?		
	2.6 Was an appropriate analysis used to estimate the effect of assignment to intervention?	Y/PY	
	2.7 If N/PN/NI to 2.6: Was there potential for a substantial impact (to the result) of the failure to analyse participants in the group to which they were randomized?		
		SOME CONCERNS	
Bias due to missing outcome data	3.1 Were data for this outcome available for all, or nearly all, participants randomized?	Y/PY	
	3.2 If N/PN/NI to 3.1: Is there evidence that the result was not biased by missing outcome data?		
	3.3 If N/PN to 3.2: Could missingness in the outcome depend on its true value?		

	3.4 If Y/PY/NI to 3.3: Is it likely that missingness in the outcome depended on its true value?		
		LOW	
Bias in measurement of the outcome	4.1 Was the method of measuring the outcome inappropriate?	Y/PY	Analgesic consumption and VAS
	4.2 Could measurement or ascertainment of the outcome have differed between intervention groups?	PN/N	
	4.3 If N/PN/NI to 4.1 and 4.2: Were outcome assessors aware of the intervention received by study participants?	Y/PY	INVESTIGATORS NOT BLINDED
	4.4 If Y/PY/NI to 4.3: Could assessment of the outcome have been influenced by knowledge of intervention received?	Y/PY	
	4.5 If Y/PY/NI to 4.4: Is it likely that assessment of the outcome was influenced by knowledge of intervention received?	PN/N	
		SOME CONCERNS	
Bias in selection of the reported result	5.1. Were the data that produced this result analysed in accordance with a pre-specified analysis plan that was finalized before unblinded outcome data were available for analysis?	PN/N	No trial registration or published protocol found. Selective outcome reporting cannot be ruled out.
	5.2. Is the numerical result being assessed likely to have been selected, on the basis of the results, from multiple eligible outcome measurements (e.g. scales, definitions, time points) within the outcome domain?		
	5.3. Is the numerical result being assessed likely to have been selected, on the basis of the results, from multiple eligible analyses of the data?		
		SOME CONCERNS	
Overall Judgment		HIGH	

**TİTLE: 14-Efficacy of bi-level erector spinae plane block versus bi-level thoracic paravertebral block for postoperative analgesia in modified radical mastectomy: a prospective randomized comparative study[30]**			
Domain	Subdomain	Risk of Bias	Justification
1.Bias arising from the randomization process	1.1. Was the allocation sequence random?	Y/PY	
	1.2. Was the allocation sequence concealed until participants were enrolled and assigned to interventions?	Y/PY	
	1.3. Did baseline differences between intervention groups suggest a problem with the randomization process?	N	
	SUBRISK	LOW	
2. Bias due to deviations from intended interventions	2.1. Were participants aware of their assigned intervention during the trial?	Y/PY	Patients and outcome assessors were blinded. However, it is not entirely clear whether intraoperative opioid titration and postoperative rescue analgesia.
	2.2. Were carers and people delivering the interventions aware of participants' assigned intervention during the trial?	Y/PY	
	2.3. If Y/PY/NI to 2.1 or 2.2: Were there deviations from the intended intervention that arose because of the trial context?	PN/N	
	2.4 If Y/PY to 2.3: Were these deviations likely to have affected the outcome?		
	2.5. If Y/PY/NI to 2.4: Were these deviations from intended intervention balanced between groups?		
	2.6 Was an appropriate analysis used to estimate the effect of assignment to intervention?	PN/N	
	2.7 If N/PN/NI to 2.6: Was there potential for a substantial impact (to the result) of the failure to analyse participants in the group to which they were randomized?	PN/N	
		SOME CONCERNS	
Bias due to missing outcome data	3.1 Were data for this outcome available for all, or nearly all, participants randomized?	Y/PY	All randomized patients (n=60) completed the study; no dropouts reported.
	3.2 If N/PN/NI to 3.1: Is there evidence that the result was not biased by missing outcome data?		

	3.3 If N/PN to 3.2: Could missingness in the outcome depend on its true value?		
	3.4 If Y/PY/NI to 3.3: Is it likely that missingness in the outcome depended on its true value?		
		LOW	
Bias in measurement of the outcome	4.1 Was the method of measuring the outcome inappropriate?	Y/PY	First analgesic consumption and VAS
	4.2 Could measurement or ascertainment of the outcome have differed between intervention groups?	PN/N	
	4.3 If N/PN/NI to 4.1 and 4.2: Were outcome assessors aware of the intervention received by study participants?	Y/PY	Not clear if INVESTIGATORS BLINDED
	4.4 If Y/PY/NI to 4.3: Could assessment of the outcome have been influenced by knowledge of intervention received?	Y/PY	
	4.5 If Y/PY/NI to 4.4: Is it likely that assessment of the outcome was influenced by knowledge of intervention received?	PN/N	
		SOME CONCERNS	
Bias in selection of the reported result	5.1. Were the data that produced this result analysed in accordance with a pre-specified analysis plan that was finalized before unblinded outcome data were available for analysis?	Y/PY	
	5.2. Is the numerical result being assessed likely to have been selected, on the basis of the results, from multiple eligible outcome measurements (e.g. scales, definitions, time points) within the outcome domain?	PN/N	
	5.3. Is the numerical result being assessed likely to have been selected, on the basis of the results, from multiple eligible analyses of the data?	PN/N	
		LOW	
Overall Judgment		SOME CONCERNS	

**TİTLE: 15-Prospective comparative analysis of intraoperative and postoperative anesthetic and analgesic effect of ultrasound-guided pectoral with serratus anterior plane block versus thoracic paravertebral versus erector spinae blocks in breast oncosurgeries[31]**			
Domain	Subdomain	Risk of Bias	Justification
1.Bias arising from the randomization process	1.1. Was the allocation sequence random?	Y/PY	Randomization performed using a computer-generated sequence
	1.2. Was the allocation sequence concealed until participants were enrolled and assigned to interventions?	Y/PY	Allocation concealment with sealed opaque envelopes
	1.3. Did baseline differences between intervention groups suggest a problem with the randomization process?	N	Baseline characteristics comparable
	SUBRISK	LOW	
2. Bias due to deviations from intended interventions	2.1. Were participants aware of their assigned intervention during the trial?	Y/PY	Described as randomized and double-blind. However, anesthesiologists administering the blocks could not be blinded due to procedural differences.
	2.2. Were carers and people delivering the interventions aware of participants' assigned intervention during the trial?	Y/PY	.
	2.3. If Y/PY/NI to 2.1 or 2.2: Were there deviations from the intended intervention that arose because of the trial context?	PN/N	
	2.4 If Y/PY to 2.3: Were these deviations likely to have affected the outcome?		
	2.5. If Y/PY/NI to 2.4: Were these deviations from intended intervention balanced between groups?		
	2.6 Was an appropriate analysis used to estimate the effect of assignment to intervention?	PN/N	
	2.7 If N/PN/NI to 2.6: Was there potential for a substantial impact (to the result) of the failure to analyse participants in the group to which they were randomized?	PN/N	

		SOME CONCERNS	
Bias due to missing outcome data	3.1 Were data for this outcome available for all, or nearly all, participants randomized?	Y/PY	
	3.2 If N/PN/NI to 3.1: Is there evidence that the result was not biased by missing outcome data?		
	3.3 If N/PN to 3.2: Could missingness in the outcome depend on its true value?		
	3.4 If Y/PY/NI to 3.3: Is it likely that missingness in the outcome depended on its true value?		
		LOW	
Bias in measurement of the outcome	4.1 Was the method of measuring the outcome inappropriate?	PN/N	First analgesic consumption and VAS
	4.2 Could measurement or ascertainment of the outcome have differed between intervention groups?	PN/N	
	4.3 If N/PN/NI to 4.1 and 4.2: Were outcome assessors aware of the intervention received by study participants?		Primary outcomes were pain scores and opioid consumption. Pain scores are subjective, and while assessors were blinded, patients might infer their group from the block effect
	4.4 If Y/PY/NI to 4.3: Could assessment of the outcome have been influenced by knowledge of intervention received?		
	4.5 If Y/PY/NI to 4.4: Is it likely that assessment of the outcome was influenced by knowledge of intervention received?		
		LOW	
Bias in selection of the reported result	5.1. Were the data that produced this result analysed in accordance with a pre-specified analysis plan that was finalized before unblinded outcome data were available for analysis?	Y/PY	Based on the results of a previous study, assuming a standard deviation of 10 mm, the sample size that was enrolled to detect a difference of 10 mm on a VAS<br>of 10 cm, with an alpha error of 0.05% and 80% as the power of the study was 65.
	5.2. Is the numerical result being assessed likely to have been selected, on the basis of the results, from multiple eligible outcome measurements (e.g. scales, definitions, time points) within the outcome domain?	PN/N	
	5.3. Is the numerical result being assessed likely to have been selected, on the basis of the results, from multiple eligible analyses of the data?	PN/N	
		LOW	
Overall Judgment		SOME CONCERNS	

TİTLE: 16-Comparison of Efficacy of Erector Spinae Plane Block with Paravertebral Block for Postoperative Analgesia in Patients Undergoing Breast Cancer Surgery[32]			
Domain	Subdomain	Risk of Bias	Justification
1.Bias arising from the randomization process	1.1. Was the allocation sequence random?	Y/PY	Randomization performed using a computer-generated sequence
	1.2. Was the allocation sequence concealed until participants were enrolled and assigned to interventions?	Y/PY	Allocation concealment with sealed opaque envelopes
	1.3. Did baseline differences between intervention groups suggest a problem with the randomization process?	N	Groups were comparable at baseline.
	SUBRISK	LOW	
2. Bias due to deviations from intended interventions	2.1. Were participants aware of their assigned intervention during the trial?	Y/PY	Although described as randomized and blinded, the anesthesiologists performing the blocks could not be blinded due to the nature of ESPB vs TPVB.
	2.2. Were carers and people delivering the interventions aware of participants' assigned intervention during the trial?	Y/PY	.
	2.3. If Y/PY/NI to 2.1 or 2.2: Were there deviations from the intended intervention that arose because of the trial context?	PN/N	
	2.4 If Y/PY to 2.3: Were these deviations likely to have affected the outcome?		
	2.5. If Y/PY/NI to 2.4: Were these deviations from intended intervention balanced between groups?		

	2.6 Was an appropriate analysis used to estimate the effect of assignment to intervention?	PN/N	
	2.7 If N/PN/NI to 2.6: Was there potential for a substantial impact (to the result) of the failure to analyse participants in the group to which they were randomized?	PN/N	
		SOME CONCERNS	
Bias due to missing outcome data	3.1 Were data for this outcome available for all, or nearly all, participants randomized?	Y/PY	
	3.2 If N/PN/NI to 3.1: Is there evidence that the result was not biased by missing outcome data?		
	3.3 If N/PN to 3.2: Could missingness in the outcome depend on its true value?		
	3.4 If Y/PY/NI to 3.3: Is it likely that missingness in the outcome depended on its true value?		
		LOW	
Bias in measurement of the outcome	4.1 Was the method of measuring the outcome inappropriate?	PN/N	First analgesic consumption and VAS
	4.2 Could measurement or ascertainment of the outcome have differed between intervention groups?	PN/N	
	4.3 If N/PN/NI to 4.1 and 4.2: Were outcome assessors aware of the intervention received by study participants?	NI	Pain scores (VAS) are subjective; even with blinded assessors, patients could infer their group from block efficacy.
	4.4 If Y/PY/NI to 4.3: Could assessment of the outcome have been influenced by knowledge of intervention received?	NI	
	4.5 If Y/PY/NI to 4.4: Is it likely that assessment of the outcome was influenced by knowledge of intervention received?	PN/N	
		SOME CONCERNS	
Bias in selection of the reported result	5.1. Were the data that produced this result analysed in accordance with a pre-specified analysis plan that was finalized before unblinded outcome data were available for analysis?	Y/PY	Based on the results of a previous study, [13] assuming a standard deviation of 10 mm, the sample size that was enrolled to detect a difference of 10 mm on a VAS of 10 cm, with an alpha error of 0.05% and 80% as the power of the study was 65.
	5.2. Is the numerical result being assessed likely to have been selected, on the basis of the results, from multiple eligible outcome measurements (e.g. scales, definitions, time points) within the outcome domain?	PN/N	
	5.3. Is the numerical result being assessed likely to have been selected, on the basis of the results, from multiple eligible analyses of the data?	PN/N	
		LOW	
Overall Judgment		SOME CONCERNS	

TİTLE: 17-A comparative study of respiratory effects of erector spinae plane block versus paravertebral plane block for women undergoing modified radical mastectomy[33]			
Domain	Subdomain	Risk of Bias	Justification
1.Bias arising from the randomization process	1.1. Was the allocation sequence random?	Y/PY	Randomization performed using a computer-generated sequence
	1.2. Was the allocation sequence concealed until participants were enrolled and assigned to interventions?	Y/PY	Allocation concealment with sealed opaque envelopes
	1.3. Did baseline differences between intervention groups suggest a problem with the randomization process?	N	Baseline characteristics well balanced.
	SUBRISK	LOW	
2. Bias due to deviations from intended interventions	2.1. Were participants aware of their assigned intervention during the trial?	PN/N	
	2.2. Were carers and people delivering the interventions aware of participants' assigned intervention during the trial?	PN/N	.
	2.3. If Y/PY/NI to 2.1 or 2.2: Were there deviations from the intended intervention that arose because of the trial context?		

	2.4 If Y/PY to 2.3: Were these deviations likely to have affected the outcome?		
	2.5. If Y/PY/NI to 2.4: Were these deviations from intended intervention balanced between groups?		
	2.6 Was an appropriate analysis used to estimate the effect of assignment to intervention?	Y/PY	
	2.7 If N/PN/NI to 2.6: Was there potential for a substantial impact (to the result) of the failure to analyse participants in the group to which they were randomized?		
		LOW	
Bias due to missing outcome data	3.1 Were data for this outcome available for all, or nearly all, participants randomized?	Y/PY	All patients completed follow-up; no attrition reported
	3.2 If N/PN/NI to 3.1: Is there evidence that the result was not biased by missing outcome data?		
	3.3 If N/PN to 3.2: Could missingness in the outcome depend on its true value?		
	3.4 If Y/PY/NI to 3.3: Is it likely that missingness in the outcome depended on its true value?		
		LOW	
Bias in measurement of the outcome	4.1 Was the method of measuring the outcome inappropriate?	Y/PY	First analgesic consumption and VAS
	4.2 Could measurement or ascertainment of the outcome have differed between intervention groups?	PN/N	
	4.3 If N/PN/NI to 4.1 and 4.2: Were outcome assessors aware of the intervention received by study participants?	Y/PY	Pain scores are subjective, and although outcome assessors were blinded, patients could infer allocation from block effec
	4.4 If Y/PY/NI to 4.3: Could assessment of the outcome have been influenced by knowledge of intervention received?	Y/PY	
	4.5 If Y/PY/NI to 4.4: Is it likely that assessment of the outcome was influenced by knowledge of intervention received?	PN/N	
		SOME CONCERNS	
Bias in selection of the reported result	5.1. Were the data that produced this result analysed in accordance with a pre-specified analysis plan that was finalized before unblinded outcome data were available for analysis?	Y/PY	
	5.2. Is the numerical result being assessed likely to have been selected, on the basis of the results, from multiple eligible outcome measurements (e.g. scales, definitions, time points) within the outcome domain?	PN/N	
	5.3. Is the numerical result being assessed likely to have been selected, on the basis of the results, from multiple eligible analyses of the data?	PN/N	
		LOW	
Overall Judgment		SOME CONCERNS	

TİTLE: 18-A Comparison of the Effectiveness of the Serratus Anterior Plane Block and Erector Spinae Plane Block to that of the Paravertebral Block in the Surgical Treatment of Breast Cancer—A Randomized, Prospective, Single-Blinded Study[34]			
Domain	Subdomain	Risk of Bias	Justification
1.Bias arising from the randomization process	1.1. Was the allocation sequence random?	Y/PY	A computer-generated table in Microsoft Excel assigned patients to the appropriate groups.
	1.2. Was the allocation sequence concealed until participants were enrolled and assigned to interventions?	Y/PY	
	1.3. Did baseline differences between intervention groups suggest a problem with the randomization process?	N	
	SUBRISK	SOME CONCERNS	
2. Bias due to deviations from intended interventions	2.1. Were participants aware of their assigned intervention during the trial?	PN/N	Although the trial was described as “double-blind,” true blinding was not possible: anesthesiologists performing ESPB vs TPVB inherently knew the allocation.
	2.2. Were carers and people delivering the interventions aware of participants' assigned intervention during the trial?		.
	2.3. If Y/PY/NI to 2.1 or 2.2: Were there deviations from the intended intervention that arose because of the trial context?		
	2.4 If Y/PY to 2.3: Were these deviations likely to have affected the outcome?		
	2.5. If Y/PY/NI to 2.4: Were these deviations from intended intervention balanced between groups?		
	2.6 Was an appropriate analysis used to estimate the effect of assignment to intervention?	Y/PY	
	2.7 If N/PN/NI to 2.6: Was there potential for a substantial impact (to the result) of the failure to analyse participants in the group to which they were randomized?	Y/PY	
		SOME CONCERNS	
Bias due to missing outcome data	3.1 Were data for this outcome available for all, or nearly all, participants randomized?	PN/N	The study reports some patient loss (SAPB (24), PVB (30), and ESPB(24))
	3.2 If N/PN/NI to 3.1: Is there evidence that the result was not biased by missing outcome data?	PN/N	
	3.3 If N/PN to 3.2: Could missingness in the outcome depend on its true value?	PN/N	
	3.4 If Y/PY/NI to 3.3: Is it likely that missingness in the outcome depended on its true value?	NI	
		SOME CONCERNS	
Bias in measurement of the outcome	4.1 Was the method of measuring the outcome inappropriate?	PN/N	VAS
	4.2 Could measurement or ascertainment of the outcome have differed between intervention groups?	NI	
	4.3 If N/PN/NI to 4.1 and 4.2: Were outcome assessors aware of the intervention received by study participants?	NI	Outcomes (pain scores, opioid use) are subjective and prone to influence from unblinded clinicians. Even if assessors were blinded, patient perception and intraoperative management could bias results.
	4.4 If Y/PY/NI to 4.3: Could assessment of the outcome have been influenced by knowledge of intervention received?	NI	
	4.5 If Y/PY/NI to 4.4: Is it likely that assessment of the outcome was influenced by knowledge of intervention received?	NI	
		HİGH	
Bias in selection of the reported result	5.1. Were the data that produced this result analysed in accordance with a pre-specified analysis plan that was finalized before unblinded outcome data were available for analysis?	PN/N	No protocol registration
	5.2. Is the numerical result being assessed likely to have been selected, on the basis of the results, from multiple eligible outcome measurements (e.g. scales, definitions, time points) within the outcome domain?		
	5.3. Is the numerical result being assessed likely to have been selected, on the basis of the results, from multiple eligible analyses of the data?		
		SOME CONCERNS	

Overall Judgment		HİGH	

TİTLE: 19- Comparison of fascial plane blocks (ESPB vs. TPVB) for pain relief following modified radical mastectomy[35]			
Domain	Subdomain	Risk of Bias	Justification
1.Bias arising from the randomization process	1.1. Was the allocation sequence random?	Y/PY	
	1.2. Was the allocation sequence concealed until participants were enrolled and assigned to interventions?	NI	No mention of allocation concealment methods
	1.3. Did baseline differences between intervention groups suggest a problem with the randomization process?	N	
	SUBRISK	SOME CONCERNS	
2. Bias due to deviations from intended interventions	2.1. Were participants aware of their assigned intervention during the trial?	NI	Anesthesiologists performing the blocks could not be blinded.
	2.2. Were carers and people delivering the interventions aware of participants' assigned intervention during the trial?	NI	.
	2.3. If Y/PY/NI to 2.1 or 2.2: Were there deviations from the intended intervention that arose because of the trial context?	PN/N	
	2.4 If Y/PY to 2.3: Were these deviations likely to have affected the outcome?		
	2.5. If Y/PY/NI to 2.4: Were these deviations from intended intervention balanced between groups?		
	2.6 Was an appropriate analysis used to estimate the effect of assignment to intervention?	PN/N	
	2.7 If N/PN/NI to 2.6: Was there potential for a substantial impact (to the result) of the failure to analyse participants in the group to which they were randomized?	PN/N	
		SOME CONCERNS	
Bias due to missing outcome data	3.1 Were data for this outcome available for all, or nearly all, participants randomized?	Y/PY	
	3.2 If N/PN/NI to 3.1: Is there evidence that the result was not biased by missing outcome data?		
	3.3 If N/PN to 3.2: Could missingness in the outcome depend on its true value?		
	3.4 If Y/PY/NI to 3.3: Is it likely that missingness in the outcome depended on its true value?		
		LOW	
Bias in measurement of the outcome	4.1 Was the method of measuring the outcome inappropriate?	PN/N	Pain scores
	4.2 Could measurement or ascertainment of the outcome have differed between intervention groups?	PN/N	
	4.3 If N/PN/NI to 4.1 and 4.2: Were outcome assessors aware of the intervention received by study participants?	Y/PY	Although patients were blinded (blocks after GA), blinding of outcome assessors was not clearly reported.
	4.4 If Y/PY/NI to 4.3: Could assessment of the outcome have been influenced by knowledge of intervention received?	Y/PY	
	4.5 If Y/PY/NI to 4.4: Is it likely that assessment of the outcome was influenced by knowledge of intervention received?	PN/N	
		SOME CONCERNS	
Bias in selection of the reported result	5.1. Were the data that produced this result analysed in accordance with a pre-specified analysis plan that was finalized before unblinded outcome data were available for analysis?	Y/PY	
	5.2. Is the numerical result being assessed likely to have been selected, on the basis of the results, from multiple eligible outcome measurements (e.g. scales, definitions, time points) within the outcome domain?	PN/N	
	5.3. Is the numerical result being assessed likely to have been selected, on the basis of the results, from multiple eligible analyses of the data?	PN/N	
		LOW	

Overall Judgment		SOME CONCERNS	

TİTLE: 20- Comparative study between the analgesic efficacy of pectoralis major block II versus erector spinae block versus paravertebral block in postoperative analgesia in modified radical mastectomy[36]			
Domain	Subdomain	Risk of Bias	Justification
1.Bias arising from the randomization process	1.1. Was the allocation sequence random?	Y/PY	
	1.2. Was the allocation sequence concealed until participants were enrolled and assigned to interventions?	Y/PY	
	1.3. Did baseline differences between intervention groups suggest a problem with the randomization process?	N	
	SUBRISK	LOW	
2. Bias due to deviations from intended interventions	2.1. Were participants aware of their assigned intervention during the trial?	Y/PY	The trial was described as randomized and double-blind, but anesthesiologists performing the interventions could not realistically be blinded.
	2.2. Were carers and people delivering the interventions aware of participants' assigned intervention during the trial?	Y/PY	
	2.3. If Y/PY/NI to 2.1 or 2.2: Were there deviations from the intended intervention that arose because of the trial context?	PN/N	
	2.4 If Y/PY to 2.3: Were these deviations likely to have affected the outcome?		
	2.5. If Y/PY/NI to 2.4: Were these deviations from intended intervention balanced between groups?		
	2.6 Was an appropriate analysis used to estimate the effect of assignment to intervention?	PN/N	
	2.7 If N/PN/NI to 2.6: Was there potential for a substantial impact (to the result) of the failure to analyse participants in the group to which they were randomized?	PN/N	
		SOME CONCERNS	
Bias due to missing outcome data	3.1 Were data for this outcome available for all, or nearly all, participants randomized?	Y/PY	
	3.2 If N/PN/NI to 3.1: Is there evidence that the result was not biased by missing outcome data?		
	3.3 If N/PN to 3.2: Could missingness in the outcome depend on its true value?		
	3.4 If Y/PY/NI to 3.3: Is it likely that missingness in the outcome depended on its true value?		
		LOW	
Bias in measurement of the outcome	4.1 Was the method of measuring the outcome inappropriate?	PN/N	First analgesic consumption and VAS
	4.2 Could measurement or ascertainment of the outcome have differed between intervention groups?	PN/N	
	4.3 If N/PN/NI to 4.1 and 4.2: Were outcome assessors aware of the intervention received by study participants?	Y/PY	Primary outcomes included pain scores and analgesic consumption, which are subjective and may be influenced by non-blinded providers.
	4.4 If Y/PY/NI to 4.3: Could assessment of the outcome have been influenced by knowledge of intervention received?	Y/PY	
	4.5 If Y/PY/NI to 4.4: Is it likely that assessment of the outcome was influenced by knowledge of intervention received?	NI	
		SOME CONCERNS	
Bias in selection of the reported result	5.1. Were the data that produced this result analysed in accordance with a pre-specified analysis plan that was finalized before unblinded outcome data were available for analysis?	Y/PY	Sample size was calculated by G power program, setting power at 80%, medium effect size (0.5), α-error = 0.05.
	5.2. Is the numerical result being assessed likely to have been selected, on the basis of the results, from multiple eligible outcome measurements (e.g. scales, definitions, time points) within the outcome domain?	PN/N	
	5.3. Is the numerical result being assessed likely to have been selected, on the basis of the results, from multiple eligible analyses of the data?	PN/N	
		LOW	
Overall Judgment		SOME CONCERNS	

TİTLE: 21- Bi-Level Ultrasound Guided Erector Spinae Block V/S Paravertebral Block in Post Operative Analgesia After Breast Cancer Surgeries[37]			
Domain	Subdomain	Risk of Bias	Justification
1.Bias arising from the randomization process	1.1. Was the allocation sequence random?	Y/PY	Randomization performed using a computer-generated sequence
	1.2. Was the allocation sequence concealed until participants were enrolled and assigned to interventions?	NI	Allocation concealment with sealed opaque envelopes
	1.3. Did baseline differences between intervention groups suggest a problem with the randomization process?	N	Baseline characteristics comparable
	SUBRISK	LOW	
2. Bias due to deviations from intended interventions	2.1. Were participants aware of their assigned intervention during the trial?	NI	
	2.2. Were carers and people delivering the interventions aware of participants' assigned intervention during the trial?	NI	.
	2.3. If Y/PY/NI to 2.1 or 2.2: Were there deviations from the intended intervention that arose because of the trial context?	PN/N	
	2.4 If Y/PY to 2.3: Were these deviations likely to have affected the outcome?		
	2.5. If Y/PY/NI to 2.4: Were these deviations from intended intervention balanced between groups?		
	2.6 Was an appropriate analysis used to estimate the effect of assignment to intervention?	Y/PY	
	2.7 If N/PN/NI to 2.6: Was there potential for a substantial impact (to the result) of the failure to analyse participants in the group to which they were randomized?		
		SOME CONCERNS	
Bias due to missing outcome data	3.1 Were data for this outcome available for all, or nearly all, participants randomized?	Y/PY	
	3.2 If N/PN/NI to 3.1: Is there evidence that the result was not biased by missing outcome data?		
	3.3 If N/PN to 3.2: Could missingness in the outcome depend on its true value?		
	3.4 If Y/PY/NI to 3.3: Is it likely that missingness in the outcome depended on its true value?		
		LOW	
Bias in measurement of the outcome	4.1 Was the method of measuring the outcome inappropriate?	PN/N	
	4.2 Could measurement or ascertainment of the outcome have differed between intervention groups?	PN/N	
	4.3 If N/PN/NI to 4.1 and 4.2: Were outcome assessors aware of the intervention received by study participants?	NI	
	4.4 If Y/PY/NI to 4.3: Could assessment of the outcome have been influenced by knowledge of intervention received?	Y/PY	
	4.5 If Y/PY/NI to 4.4: Is it likely that assessment of the outcome was influenced by knowledge of intervention received?	NI	
		SOME CONCERNS	
Bias in selection of the reported result	5.1 Were the data that produced this result analysed in accordance with a pre-specified analysis plan that was finalized before unblinded outcome data were available for analysis?	Y/PY	Sample size of 27 was required per group for the comparison keeping in view at most 5% risk, with minimum 80% power and significance level of 5% (significant at 95% confidence interval).
	5.2. Is the numerical result being assessed likely to have been selected, on the basis of the results, from multiple eligible outcome measurements (e.g. scales, definitions, time points) within the outcome domain?	PN/N	
	5.3. Is the numerical result being assessed likely to have been selected, on the basis of the results, from multiple eligible analyses of the data?	PN/N	
		LOW	
Overall Judgment		SOME CONCERNS	

TİTLE: 22- Comparison of Feasibility of Ultrasound Guided Erector Spinae Plane Block versus Paravertebral Plane Block in Modified Radical Mastectomies by Anaesthesiology Residents - A Randomised Control Trail[38]			
Domain	Subdomain	Risk of Bias	Justification
1.Bias arising from the randomization process	1.1. Was the allocation sequence random?	Y/PY	
	1.2. Was the allocation sequence concealed until participants were enrolled and assigned to interventions?	Y/PY	Closed envelope procedure
	1.3. Did baseline differences between intervention groups suggest a problem with the randomization process?	N	
	SUBRISK	LOW	
2. Bias due to deviations from intended interventions	2.1. Were participants aware of their assigned intervention during the trial?	Y/PY	The study was described as randomized, but not all details about blinding were provided
	2.2. Were carers and people delivering the interventions aware of participants' assigned intervention during the trial?	Y/PY	.
	2.3. If Y/PY/NI to 2.1 or 2.2: Were there deviations from the intended intervention that arose because of the trial context?	PN/N	
	2.4 If Y/PY to 2.3: Were these deviations likely to have affected the outcome?		
	2.5. If Y/PY/NI to 2.4: Were these deviations from intended intervention balanced between groups?		
	2.6 Was an appropriate analysis used to estimate the effect of assignment to intervention?	PN/N	
	2.7 If N/PN/NI to 2.6: Was there potential for a substantial impact (to the result) of the failure to analyse participants in the group to which they were randomized?	PN/N	
		SOME CONCERNS	
Bias due to missing outcome data	3.1 Were data for this outcome available for all, or nearly all, participants randomized?	Y/PY	Some patients were excluded from analysis, and the handling of missing data was not clearly explained.
	3.2 If N/PN/NI to 3.1: Is there evidence that the result was not biased by missing outcome data?		
	3.3 If N/PN to 3.2: Could missingness in the outcome depend on its true value?		
	3.4 If Y/PY/NI to 3.3: Is it likely that missingness in the outcome depended on its true value?		
		HIGH	
Bias in measurement of the outcome	4.1 Was the method of measuring the outcome inappropriate?	PN/N	Pain scores
	4.2 Could measurement or ascertainment of the outcome have differed between intervention groups?	PN/N	
	4.3 If N/PN/NI to 4.1 and 4.2: Were outcome assessors aware of the intervention received by study participants?	NI	Outcomes are subjective. While assessors were reportedly blinded, lack of clarity on blinding procedures raises potential detection bias.
	4.4 If Y/PY/NI to 4.3: Could assessment of the outcome have been influenced by knowledge of intervention received?	Y/PY	
	4.5 If Y/PY/NI to 4.4: Is it likely that assessment of the outcome was influenced by knowledge of intervention received?	NI	
		SOME CONCERNS	
Bias in selection of the reported result	5.1. Were the data that produced this result analysed in accordance with a pre-specified analysis plan that was finalized before unblinded outcome data were available for analysis?	N	The study carried out by Moustafa et al. [3] found that the sample size was calculated as 43 and that, after accounting for 2% more for loss, it was computed as 50 in each group.
	5.2. Is the numerical result being assessed likely to have been selected, on the basis of the results, from multiple eligible outcome measurements (e.g. scales, definitions, time points) within the outcome domain?	PN/N	No trial registration or published protocol
	5.3. Is the numerical result being assessed likely to have been selected, on the basis of the results, from multiple eligible analyses of the data?	PN/N	
		SOME CONCERNS	

Overall Judgment		HIGH	

Title 23- Comparison between ultrasound guided erector spinae plane block and paravertebral block on acute and chronic postmastectomy pain after modified radical mastectomy: randomized controlled trial[39]			
Domain	Subdomain	Risk of Bias	Justification
1.Bias arising from the randomization process	1.1. Was the allocation sequence random?	Y/PY	Randomized, single blind study: randomly allocated by computer generated random numbers into two groups
	1.2. Was the allocation sequence concealed until participants were enrolled and assigned to interventions?	NI	
	1.3. Did baseline differences between intervention groups suggest a problem with the randomization process?	PN/N	Baseline characteristics were similar
	SUBRISK	LOW	
2. Bias due to deviations from intended interventions	2.1. Were participants aware of their assigned intervention during the trial?	PN/N	The trial was labeled as randomized and double-blind
	2.2. Were carers and people delivering the interventions aware of participants' assigned intervention during the trial?	Y/PY	
	2.3. If Y/PY/NI to 2.1 or 2.2: Were there deviations from the intended intervention that arose because of the trial context?	PN/N	
	2.4 If Y/PY to 2.3: Were these deviations likely to have affected the outcome?	PN/N	
	2.5. If Y/PY/NI to 2.4: Were these deviations from intended intervention balanced between groups?		
	2.6 Was an appropriate analysis used to estimate the effect of assignment to intervention?	Y/PY	
	2.7 If N/PN/NI to 2.6: Was there potential for a substantial impact (to the result) of the failure to analyse participants in the group to which they were randomized?		
	SUBRISK	LOW	
Bias due to missing outcome data	3.1 Were data for this outcome available for all, or nearly all, participants randomized?	Y/PY	No missing data
	3.2 If N/PN/NI to 3.1: Is there evidence that the result was not biased by missing outcome data?		
	3.3 If N/PN to 3.2: Could missingness in the outcome depend on its true value?		
	3.4 If Y/PY/NI to 3.3: Is it likely that missingness in the outcome depended on its true value?		
	SUBRISK	LOW	
Bias in measurement of the outcome	4.1 Was the method of measuring the outcome inappropriate?	Y/PY	VAS, Time to first rescue analgesia
	4.2 Could measurement or ascertainment of the outcome have differed between intervention groups?	PN/N	
	4.3 If N/PN/NI to 4.1 and 4.2: Were outcome assessors aware of the intervention received by study participants?	PN/N	Authors involved in data collection were blinded to the block
	4.4 If Y/PY/NI to 4.3: Could assessment of the outcome have been influenced by knowledge of intervention received?		
	4.5 If Y/PY/NI to 4.4: Is it likely that assessment of the outcome was influenced by knowledge of intervention received?		
	SUBRISK	LOW	
Bias in selection of the reported result	5.1. Were the data that produced this result analysed in accordance with a pre-specified analysis plan that was finalized before unblinded outcome data were available for analysis?	Y/PY	G*Power 3 software 1 was used to compute the sample size. A minimum of 102 women who were candidates for mastectomy surgery were randomly allocated to one of three equal groups (n=34).
	5.2. Is the numerical result being assessed likely to have been selected, on the basis of the results, from multiple eligible outcome measurements (e.g. scales, definitions, time points) within the outcome domain?	PN/N	
	5.3. Is the numerical result being assessed likely to have been selected, on the basis of the results, from multiple eligible analyses of the data?	PN/N	

	SUBRISK	LOW	
Overall Judgment		LOW	

**Title 24 -Erector spinae plane block versus paravertebral block for major oncological breast surgery: a multicentre randomised controlled trial[40]**			
Domain	Subdomain	Risk of Bias	Justification
1.Bias arising from the randomization process	1.1. Was the allocation sequence random?	Y/PY	Centralized web-based randomization
	1.2. Was the allocation sequence concealed until participants were enrolled and assigned to interventions?	NI	With concealment
	1.3. Did baseline differences between intervention groups suggest a problem with the randomization process?	PN/N	Baseline groups balanced
	SUBRISK	LOW	
2. Bias due to deviations from intended interventions	2.1. Were participants aware of their assigned intervention during the trial?	PN/N	Double-blind
	2.2. Were carers and people delivering the interventions aware of participants' assigned intervention during the trial?	Y/PY	
	2.3. If Y/PY/NI to 2.1 or 2.2: Were there deviations from the intended intervention that arose because of the trial context?	PN/N	
	2.4 If Y/PY to 2.3: Were these deviations likely to have affected the outcome?	PN/N	
	2.5. If Y/PY/NI to 2.4: Were these deviations from intended intervention balanced between groups?		
	2.6 Was an appropriate analysis used to estimate the effect of assignment to intervention?	Y/PY	ITT analysis used; standardized perioperative protocols.
	2.7 If N/PN/NI to 2.6: Was there potential for a substantial impact (to the result) of the failure to analyse participants in the group to which they were randomized?		
	SUBRISK	LOW	
Bias due to missing outcome data	3.1 Were data for this outcome available for all, or nearly all, participants randomized?	Y/PY	ITT and PP analyses consistent.
	3.2 If N/PN/NI to 3.1: Is there evidence that the result was not biased by missing outcome data?		
	3.3 If N/PN to 3.2: Could missingness in the outcome depend on its true value?		
	3.4 If Y/PY/NI to 3.3: Is it likely that missingness in the outcome depended on its true value?		
	SUBRISK	LOW	
Bias in measurement of the outcome	4.1 Was the method of measuring the outcome inappropriate?	Y/PY	VAS, Time to first rescue analgesia
	4.2 Could measurement or ascertainment of the outcome have differed between intervention groups?	PN/N	
	4.3 If N/PN/NI to 4.1 and 4.2: Were outcome assessors aware of the intervention received by study participants?	PN/N	Authors involved in data collection were blinded to the block
	4.4 If Y/PY/NI to 4.3: Could assessment of the outcome have been influenced by knowledge of intervention received?		
	4.5 If Y/PY/NI to 4.4: Is it likely that assessment of the outcome was influenced by knowledge of intervention received?		
	SUBRISK	LOW	
Bias in selection of the reported result	5.1. Were the data that produced this result analysed in accordance with a pre-specified analysis plan that was finalized before unblinded outcome data were available for analysis?	Y/PY	Outcomes reported as prespecified post-hoc superiority analysis clearly labeled exploratory.
	5.2. Is the numerical result being assessed likely to have been selected, on the basis of the results, from multiple	PN/N	Trial registered

	eligible outcome measurements (e.g. scales, definitions, time points) within the outcome domain?		
	5.3. Is the numerical result being assessed likely to have been selected, on the basis of the results, from multiple eligible analyses of the data?	PN/N	
	SUBRISK	LOW	
Overall Judgment		LOW	

TİTLE: 25- Comparison of Efficacy of Ultrasound Guided Erector Spinae Block vs Paravertebral Block for Postoperative Analgesia in Breast Surgeries[41]			
Domain	Subdomain	Risk of Bias	Justification
1.Bias arising from the randomization process	1.1. Was the allocation sequence random?	NI	Randomization/concealment not described
	1.2. Was the allocation sequence concealed until participants were enrolled and assigned to interventions?	N	
	1.3. Did baseline differences between intervention groups suggest a problem with the randomization process?	N	Baseline SBP and VAS imbalanced; outcomes are subjective
	SUBRISK	SOME CONCERNS	
2. Bias due to deviations from intended interventions	2.1. Were participants aware of their assigned intervention during the trial?	Y/PY	Unclear blinding
	2.2. Were carers and people delivering the interventions aware of participants' assigned intervention during the trial?	Y/PY	.
	2.3. If Y/PY/NI to 2.1 or 2.2: Were there deviations from the intended intervention that arose because of the trial context?	PN/N	
	2.4 If Y/PY to 2.3: Were these deviations likely to have affected the outcome?		
	2.5. If Y/PY/NI to 2.4: Were these deviations from intended intervention balanced between groups?		
	2.6 Was an appropriate analysis used to estimate the effect of assignment to intervention?	PN/N	
	2.7 If N/PN/NI to 2.6: Was there potential for a substantial impact (to the result) of the failure to analyse participants in the group to which they were randomized?	PN/N	
		SOME CONCERNS	
Bias due to missing outcome data	3.1 Were data for this outcome available for all, or nearly all, participants randomized?	Y/PY	No missing data
	3.2 If N/PN/NI to 3.1: Is there evidence that the result was not biased by missing outcome data?		
	3.3 If N/PN to 3.2: Could missingness in the outcome depend on its true value?		
	3.4 If Y/PY/NI to 3.3: Is it likely that missingness in the outcome depended on its true value?		
		LOW	
Bias in measurement of the outcome	4.1 Was the method of measuring the outcome inappropriate?	Y/PY	First analgesic consumption and VAS
	4.2 Could measurement or ascertainment of the outcome have differed between intervention groups?	PN/N	
	4.3 If N/PN/NI to 4.1 and 4.2: Were outcome assessors aware of the intervention received by study participants?	Y/PY	Not clear if INVESTIGATORS BLINDED
	4.4 If Y/PY/NI to 4.3: Could assessment of the outcome have been influenced by knowledge of intervention received?	Y/PY	
	4.5 If Y/PY/NI to 4.4: Is it likely that assessment of the outcome was influenced by knowledge of intervention received?	PN/N	
		HIGH	
Bias in selection of the reported result	5.1. Were the data that produced this result analysed in accordance with a pre-specified analysis plan that was finalized before unblinded outcome data were available for analysis?	Y/PY	No preregistered protocol or prespecified primary time-point
	5.2. Is the numerical result being assessed likely to have been selected, on the basis of the results, from multiple	PN/N	

	eligible outcome measurements (e.g. scales, definitions, time points) within the outcome domain?		
	5.3. Is the numerical result being assessed likely to have been selected, on the basis of the results, from multiple eligible analyses of the data?	PN/N	
		SOME CONCERNS	
Overall Judgment		HIGH	

**Title 26 -Erector spinae plane block versus paravertebral block for major oncological breast surgery: a multicentre randomised controlled trial[42]**			
Domain	Subdomain	Risk of Bias	Justification
1.Bias arising from the randomization process	1.1. Was the allocation sequence random?	Y/PY	Computer-generated random sequence
	1.2. Was the allocation sequence concealed until participants were enrolled and assigned to interventions?	NI	Allocation concealment via opaque envelopes
	1.3. Did baseline differences between intervention groups suggest a problem with the randomization process?	PN/N	Baseline balanced
	SUBRISK	LOW	
2. Bias due to deviations from intended interventions	2.1. Were participants aware of their assigned intervention during the trial?	PN/N	Blocks performed by an independent anesthesiologist; outcome assessors blinded; standardized anesthesia/analgesia protocols; all randomized patients analyzed .
	2.2. Were carers and people delivering the interventions aware of participants' assigned intervention during the trial?	Y/PY	
	2.3. If Y/PY/NI to 2.1 or 2.2: Were there deviations from the intended intervention that arose because of the trial context?	PN/N	
	2.4 If Y/PY to 2.3: Were these deviations likely to have affected the outcome?	PN/N	
	2.5. If Y/PY/NI to 2.4: Were these deviations from intended intervention balanced between groups?		
	2.6 Was an appropriate analysis used to estimate the effect of assignment to intervention?	Y/PY	
	2.7 If N/PN/NI to 2.6: Was there potential for a substantial impact (to the result) of the failure to analyse participants in the group to which they were randomized?		
	SUBRISK	LOW	
Bias due to missing outcome data	3.1 Were data for this outcome available for all, or nearly all, participants randomized?	Y/PY	
	3.2 If N/PN/NI to 3.1: Is there evidence that the result was not biased by missing outcome data?		No missing data
	3.3 If N/PN to 3.2: Could missingness in the outcome depend on its true value?		
	3.4 If Y/PY/NI to 3.3: Is it likely that missingness in the outcome depended on its true value?		
	SUBRISK	LOW	
Bias in measurement of the outcome	4.1 Was the method of measuring the outcome inappropriate?	Y/PY	VAS, subjective outcome
	4.2 Could measurement or ascertainment of the outcome have differed between intervention groups?	PN/N	
	4.3 If N/PN/NI to 4.1 and 4.2: Were outcome assessors aware of the intervention received by study participants?	PN/N	Although outcome assessors were blinded, patients were not blinded
	4.4 If Y/PY/NI to 4.3: Could assessment of the outcome have been influenced by knowledge of intervention received?		

	4.5 If Y/PY/NI to 4.4: Is it likely that assessment of the outcome was influenced by knowledge of intervention received?		
	SUBRISK	SOME CONCERNS	
Bias in selection of the reported result	5.1. Were the data that produced this result analysed in accordance with a pre-specified analysis plan that was finalized before unblinded outcome data were available for analysis?	NI	Needed to enroll 22 patients in each group to reject the null hypothesis that the two groups' population means are identical with a power of 0.8, according to these results. The probability of a Type I error in this test of the null hypothesis is 0.05.
	5.2. Is the numerical result being assessed likely to have been selected, on the basis of the results, from multiple eligible outcome measurements (e.g. scales, definitions, time points) within the outcome domain?	PN/N	No Trial registered
	5.3. Is the numerical result being assessed likely to have been selected, on the basis of the results, from multiple eligible analyses of the data?	PN/N	
	SUBRISK	SOME CONCERNS	
Overall Judgment		SOME CONCERNS	

TİTLE: 27- Postoperative pain relief in patients Undergoing unilateral modified radical Mastectomy; comparison between Ultrasound guided erector spinae plane Block versus ultra sound guided thoracic Paravertebral block[43]			
Domain	Subdomain	Risk of Bias	Justification
1.Bias arising from the randomization process	1.1. Was the allocation sequence random?	NI	Block randomization was used, but randomization was performed by a single anesthetist.
	1.2. Was the allocation sequence concealed until participants were enrolled and assigned to interventions?	N	No details on allocation concealment
	1.3. Did baseline differences between intervention groups suggest a problem with the randomization process?	N	
	SUBRISK	SOME CONCERNS	
2. Bias due to deviations from intended interventions	2.1. Were participants aware of their assigned intervention during the trial?	Y/PY	Unclear blinding
	2.2. Were carers and people delivering the interventions aware of participants' assigned intervention during the trial?	Y/PY	No explicit mention of blinding of patients, clinicians, or assessors
	2.3. If Y/PY/NI to 2.1 or 2.2: Were there deviations from the intended intervention that arose because of the trial context?	PN/N	
	2.4 If Y/PY to 2.3: Were these deviations likely to have affected the outcome?		
	2.5. If Y/PY/NI to 2.4: Were these deviations from intended intervention balanced between groups?		
	2.6 Was an appropriate analysis used to estimate the effect of assignment to intervention?	PN/N	
	2.7 If N/PN/NI to 2.6: Was there potential for a substantial impact (to the result) of the failure to analyse participants in the group to which they were randomized?	PN/N	
		SOME CONCERNS	
Bias due to missing outcome data	3.1 Were data for this outcome available for all, or nearly all, participants randomized?	Y/PY	No attrition reported.
	3.2 If N/PN/NI to 3.1: Is there evidence that the result was not biased by missing outcome data?		
	3.3 If N/PN to 3.2: Could missingness in the outcome depend on its true value?		
	3.4 If Y/PY/NI to 3.3: Is it likely that missingness in the outcome depended on its true value?		
		LOW	
Bias in measurement of the outcome	4.1 Was the method of measuring the outcome inappropriate?	PN/N	VAS pain at 12 hours
	4.2 Could measurement or ascertainment of the outcome have differed between intervention groups?	Y/PY	
	4.3 If N/PN/NI to 4.1 and 4.2: Were outcome assessors aware of the intervention received by study participants?	NI	With no patient blinding, risk of biased reporting is present.
	4.4 If Y/PY/NI to 4.3: Could assessment of the outcome have been influenced by knowledge of intervention received?	NI	
	4.5 If Y/PY/NI to 4.4: Is it likely that assessment of the outcome was influenced by knowledge of intervention received?	y	
		HIGH	
Bias in selection of the reported result	5.1. Were the data that produced this result analysed in accordance with a pre-specified analysis plan that was finalized before unblinded outcome data were available for analysis?	Y/PY	No trial registration or published protocol identified
	5.2. Is the numerical result being assessed likely to have been selected, on the basis of the results, from multiple eligible outcome measurements (e.g. scales, definitions, time points) within the outcome domain?	PN/N	
	5.3. Is the numerical result being assessed likely to have been selected, on the basis of the results, from multiple eligible analyses of the data?	PN/N	
		SOME CONCERNS	
Overall Judgment		HIGH	

Forest and Funnel plots for both primary and secondary outcomes and sensitivity analysis

MME consumption at 24h - Funnel Plot

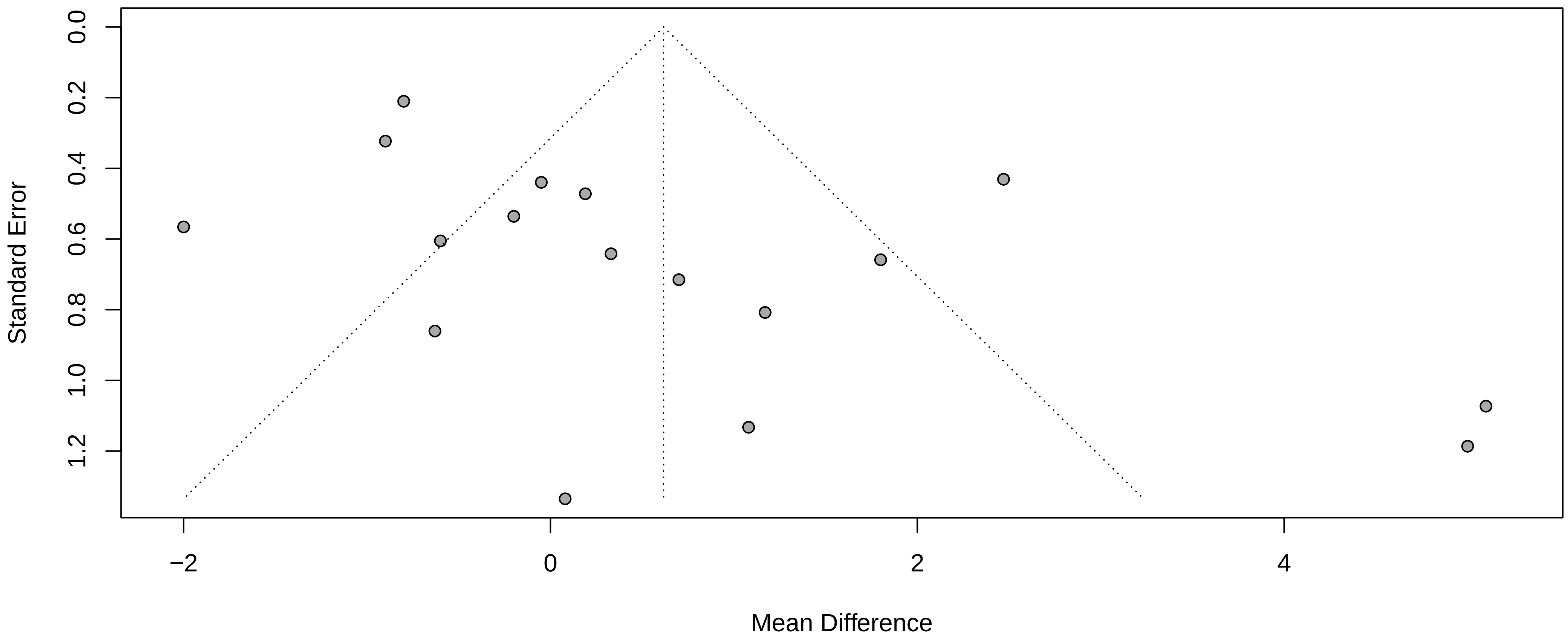

Pain at 0 h on Rest - Forest Plot

Study	ESPB Total	ESPB Mean	ESPB SD	PVB Total	PVB Mean	PVB SD	MD	95%−CI	Weight
Agarwal (2021)	40	2.12	1.45	40	2.21	1.16	−0.09	[−0.67; 0.49]	5.7%
Wittayapairoj (2022)	22	4.41	1.44	22	2.73	1.78	1.68	[0.72; 2.64]	3.4%
Sivrikoz (2022)	42	3.35	0.77	41	2.65	0.77	0.70	[0.37; 1.03]	7.6%
Elewa (2022)	30	1.36	0.78	30	1.36	0.78	0.00	[−0.39; 0.39]	7.1%
Amr (2024)	35	3.14	0.49	35	3.30	0.50	−0.16	[−0.39; 0.07]	8.3%
Sayed (2024)	20	0.15	0.27	20	0.00	0.01	0.15	[0.03; 0.27]	8.9%
Kamel (2025)	22	1.08	2.38	22	0.72	1.59	0.36	[−0.84; 1.56]	2.5%
El Ghamry (2019)	35	1.50	0.77	35	1.64	0.77	−0.14	[−0.50; 0.22]	7.4%
Gurkan (2020)	25	2.00	2.35	25	0.71	1.57	1.29	[0.18; 2.40]	2.8%
Singh (2023)	30	1.30	0.35	30	1.18	0.42	0.12	[−0.08; 0.32]	8.6%
But (2024)	24	3.90	1.60	30	4.00	1.70	−0.10	[−0.98; 0.78]	3.7%
Eldemrdash (2019)	25	2.37	1.35	25	2.37	1.35	0.00	[−0.75; 0.75]	4.5%
Kangle (2023)	25	1.52	1.00	25	0.72	0.97	0.80	[0.25; 1.35]	5.9%
Nawar (2024)	50	1.70	0.50	50	2.00	0.10	−0.30	[−0.44; −0.16]	8.8%
Eskandr (2022)	20	1.49	0.56	20	0.77	0.75	0.72	[0.31; 1.13]	7.0%
Yendrapati (2024)	50	2.60	0.45	50	2.40	1.02	0.20	[−0.11; 0.51]	7.8%
**Random effects model**	**495**			**500**			**0.23**	**[0.01; 0.45]**	**100.0%**

Heterogeneity: $I^2 = 81.4\%$, $\tau^2 = 0.1394$, $p < 0.0001$

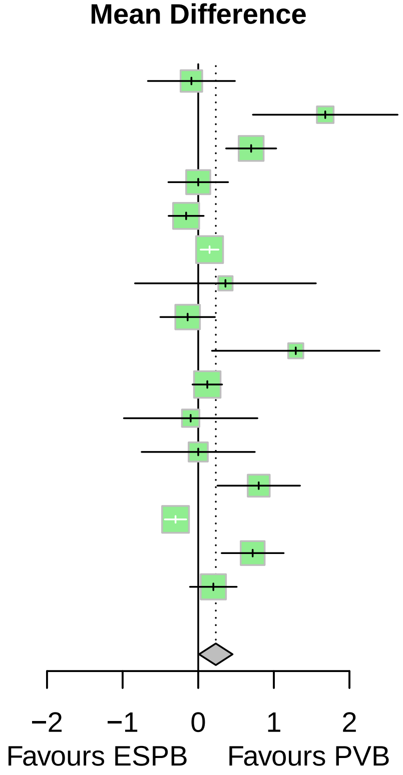

Pain at 0 h on Rest - Funnel Plot

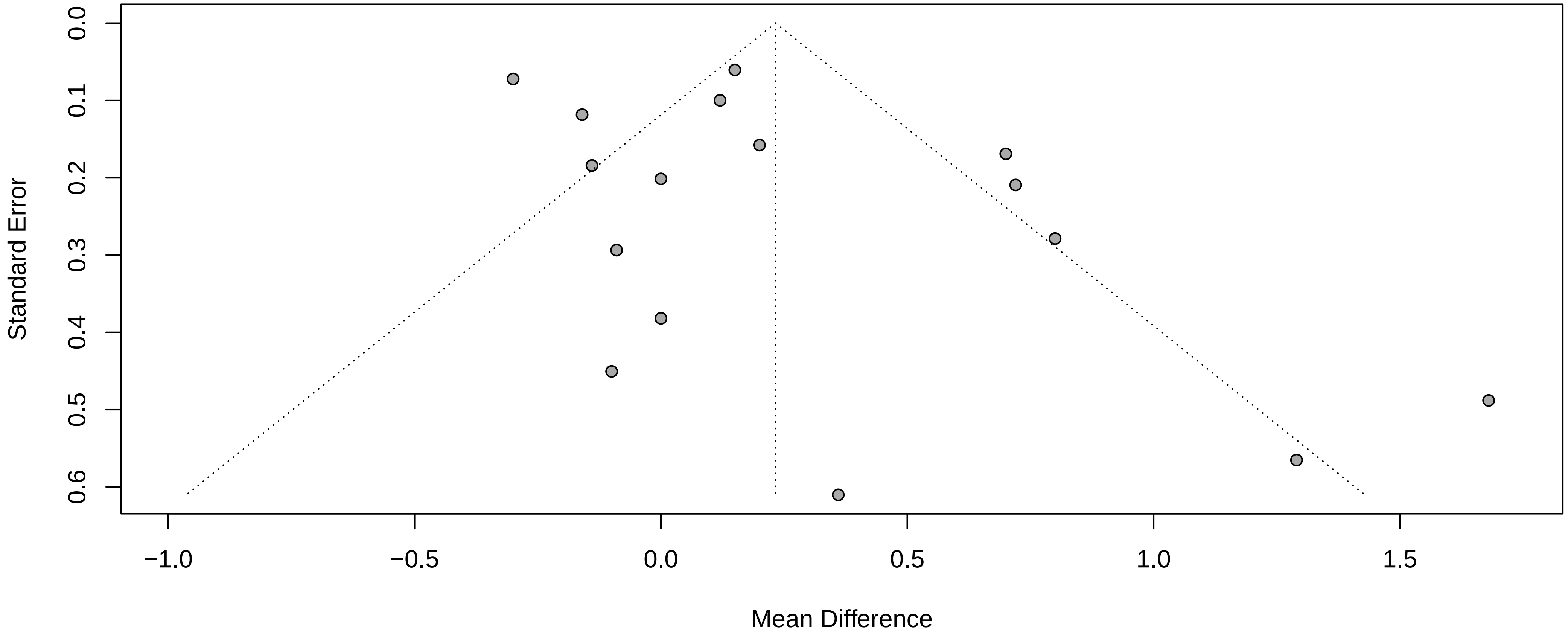

Pain at 0 h on Movement - Forest Plot

Study	ESPB Total	ESPB Mean	ESPB SD	PVB Total	PVB Mean	PVB SD	MD	95%−CI	Weight
Agarwal (2021)	40	3.12	1.45	40	3.64	0.72	−0.52	[−1.02; −0.02]	13.5%
Wittayapairoj (2022)	22	6.05	1.94	22	3.55	2.22	2.50	[1.27; 3.73]	7.7%
Sivrikoz (2022)	42	4.65	0.77	41	4.00	1.54	0.65	[0.12; 1.18]	13.3%
Amr (2024)	35	3.80	0.23	35	3.11	0.65	0.69	[0.46; 0.92]	15.3%
Kamel (2025)	22	1.71	1.58	22	1.71	1.58	0.00	[−0.93; 0.93]	9.8%
Singh (2023)	30	0.90	0.71	30	0.90	0.48	0.00	[−0.31; 0.31]	14.9%
But (2024)	24	4.80	1.40	30	4.80	1.70	0.00	[−0.83; 0.83]	10.7%
Eskandr (2022)	20	2.22	0.41	20	1.88	0.65	0.34	[0.00; 0.68]	14.7%
**Random effects model**	**235**			**240**			**0.36**	**[−0.11; 0.84]**	**100.0%**

Heterogeneity: $I^2 = 82.1\%$, $\tau^2 = 0.3664$, $p < 0.0001$

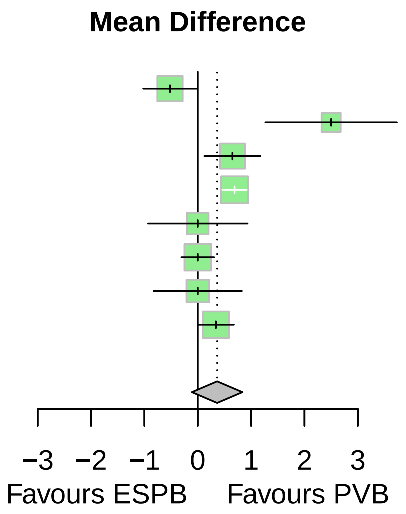

Pain at 0 h on Movement - Funnel Plot

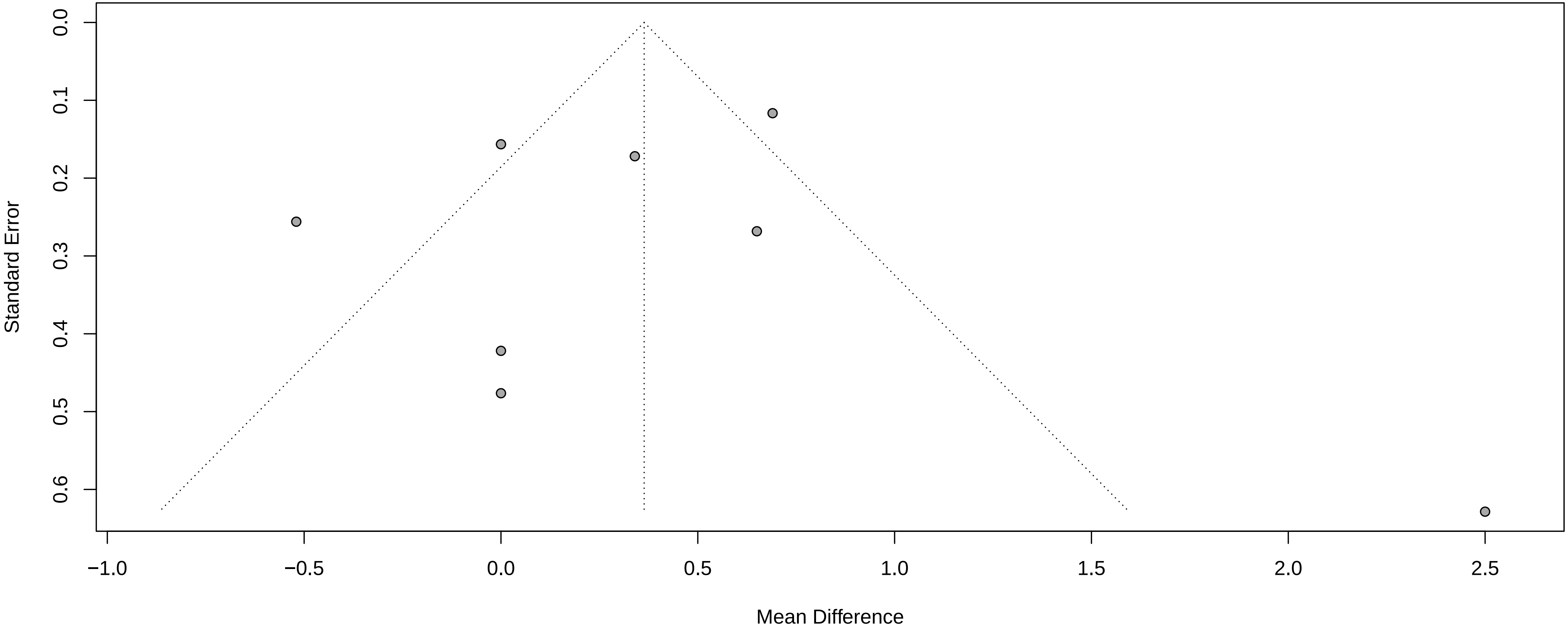

Pain at 6 h on Rest - Forest Plot

Study	ESPB Total	ESPB Mean	ESPB SD	PVB Total	PVB Mean	PVB SD	MD	95%−CI	Weight
Agarwal (2021)	40	2.70	0.58	40	2.64	0.72	0.06	[−0.23; 0.35]	8.0%
Wittayapairoj (2022)	22	2.09	1.72	22	1.32	1.17	0.77	[−0.10; 1.64]	4.4%
Sivrikoz (2022)	42	1.35	2.30	41	0.71	1.54	0.64	[−0.20; 1.48]	4.6%
Elewa (2022)	30	4.00	1.56	30	5.18	1.17	−1.18	[−1.88; −0.48]	5.4%
Amr (2024)	35	3.34	0.78	35	3.26	0.59	0.08	[−0.24; 0.40]	7.8%
Jayakrishnan (2024)	30	0.47	0.50	30	0.47	0.57	0.00	[−0.27; 0.27]	8.1%
Sayed (2024)	20	1.85	0.27	20	1.85	0.27	0.00	[−0.17; 0.17]	8.5%
Kamel (2025)	22	3.00	3.17	22	3.00	3.17	0.00	[−1.87; 1.87]	1.6%
El Ghamry (2019)	35	3.00	1.54	35	3.14	1.54	−0.14	[−0.86; 0.58]	5.3%
Gurkan (2020)	25	1.00	1.57	25	0.82	1.17	0.18	[−0.59; 0.95]	5.0%
Singh (2023)	30	2.43	0.50	30	2.33	0.48	0.10	[−0.15; 0.35]	8.2%
Eldemrdash (2019)	25	2.35	2.37	25	2.64	2.37	−0.29	[−1.60; 1.02]	2.7%
Kangle (2023)	25	1.60	0.91	25	0.92	0.75	0.68	[0.22; 1.14]	6.9%
Nawar (2024)	50	2.90	0.60	50	2.90	0.70	0.00	[−0.26; 0.26]	8.1%
Eskandr (2022)	20	2.44	0.41	20	1.96	0.75	0.48	[0.11; 0.85]	7.5%
Yendrapati (2024)	50	2.80	0.31	50	3.80	0.98	−1.00	[−1.28; −0.72]	8.0%
**Random effects model**	**501**			**500**			**0.02**	**[−0.24; 0.28]**	**100.0%**

Heterogeneity: $I^2 = 81.1\%$, $\tau^2 = 0.1979$, $p < 0.0001$

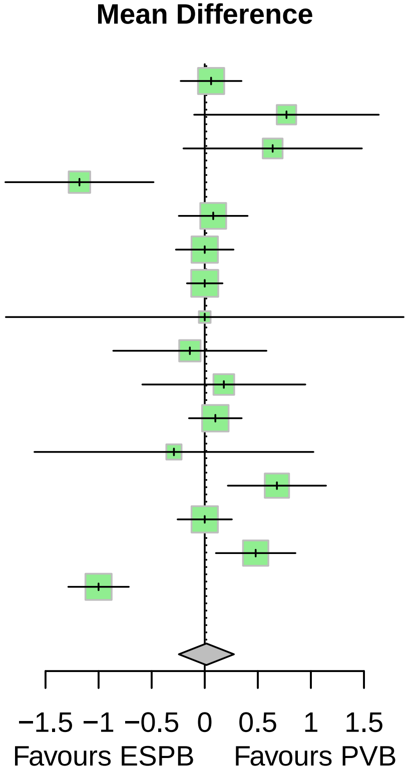

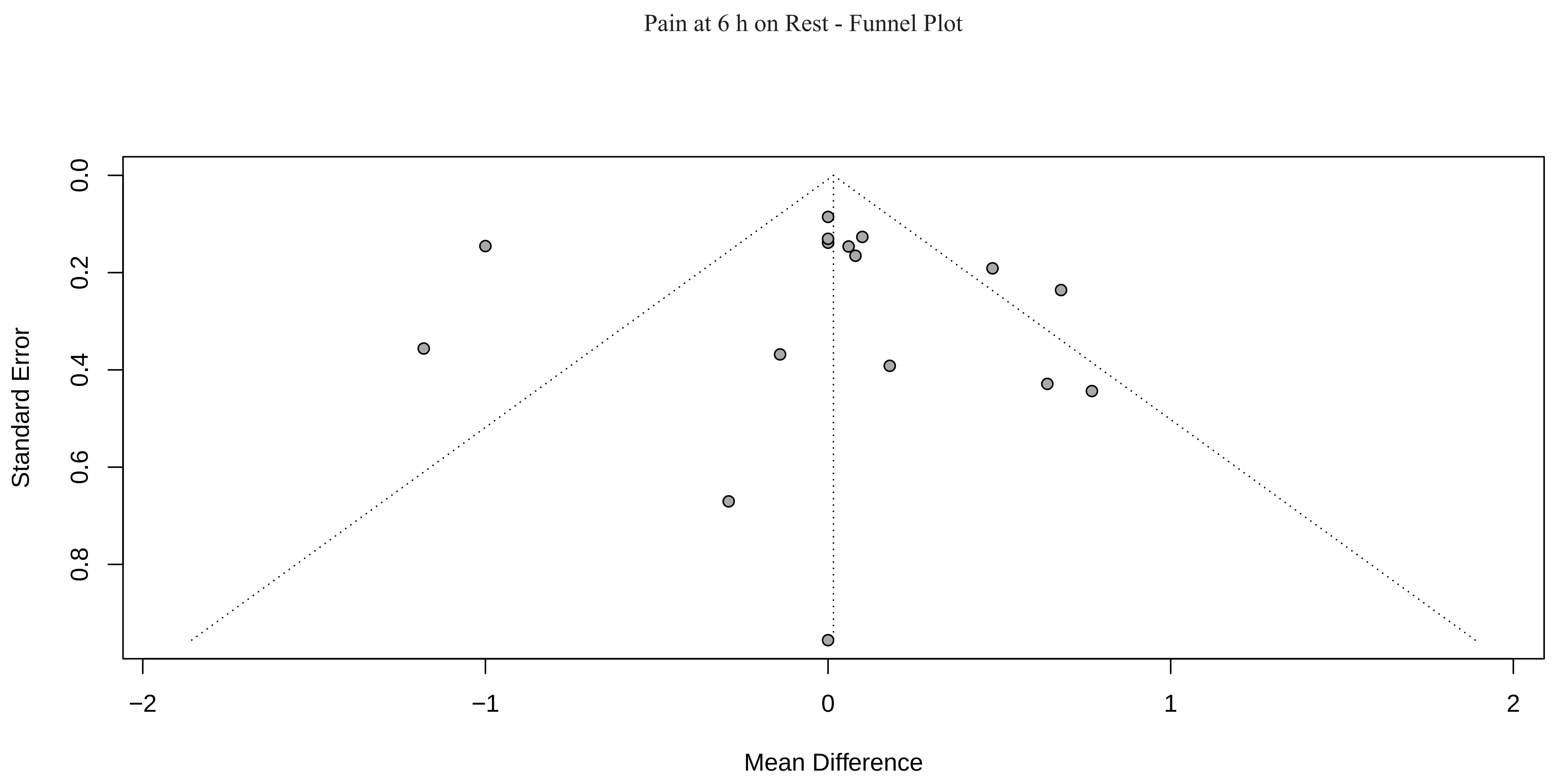
Pain at 6 h on Rest - Funnel Plot
Standard Error
0.0
0.2
0.4
0.6
0.8
−2
−1
0
1
2
Mean Difference

Pain at 6 h on Movement - Forest Plot

Study	ESPB Total	ESPB Mean	ESPB SD	PVB Total	PVB Mean	PVB SD	MD	95%−CI	Weight
Agarwal (2021)	40	2.64	0.72	40	3.00	0.01	−0.36	[−0.58; −0.14]	21.4%
Wittayapairoj (2022)	22	3.41	1.18	22	2.91	1.27	0.50	[−0.22; 1.22]	11.8%
Sivrikoz (2022)	42	2.29	3.07	41	1.42	3.07	0.87	[−0.45; 2.19]	5.5%
Amr (2024)	35	3.83	0.54	35	3.31	0.67	0.52	[0.23; 0.81]	20.3%
Kamel (2025)	22	3.35	2.37	22	3.71	3.17	−0.36	[−2.01; 1.29]	3.8%
Singh (2023)	30	2.63	0.49	30	2.57	0.50	0.06	[−0.19; 0.31]	20.9%
Eskandr (2022)	20	3.52	0.99	20	2.94	0.48	0.58	[0.10; 1.06]	16.3%
**Random effects model**	**211**			**210**			**0.23**	**[−0.12; 0.58]**	**100.0%**

Heterogeneity: $I^2 = 80.7\%$, $\tau^2 = 0.1381$, $p < 0.0001$

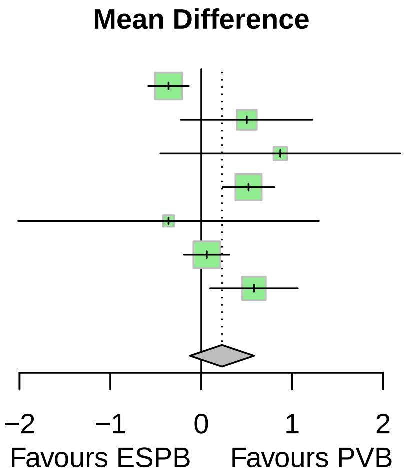

Pain at 6 h on Movement - Funnel Plot

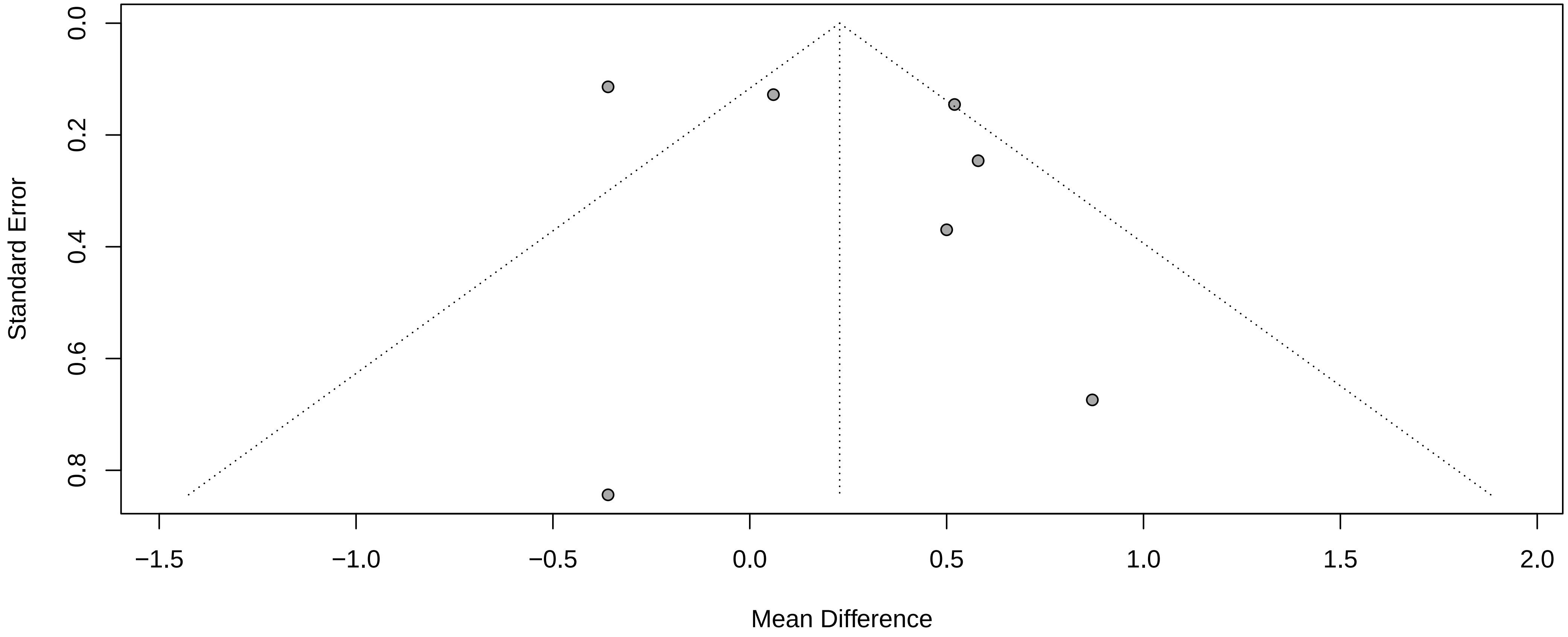

Pain at 12 h on Rest - Forest Plot

Study	ESPB Total	ESPB Mean	ESPB SD	PVB Total	PVB Mean	PVB SD	MD	95%−CI	Weight
Agarwal (2021)	40	1.70	0.58	40	1.64	0.72	0.06	[−0.23; 0.35]	6.9%
Wittayapairoj (2022)	22	1.23	1.11	22	0.68	0.95	0.55	[−0.06; 1.16]	5.7%
Sivrikoz (2022)	42	0.35	0.77	41	0.00	0.01	0.35	[0.12; 0.58]	7.0%
Elewa (2022)	30	5.27	0.97	30	5.82	1.17	−0.55	[−1.09; −0.01]	6.0%
Amr (2024)	35	3.57	0.23	35	3.34	0.83	0.23	[−0.06; 0.52]	6.9%
Jayakrishnan (2024)	30	0.63	0.56	30	0.70	0.59	−0.07	[−0.36; 0.22]	6.9%
Sayed (2024)	20	3.70	0.54	20	2.85	0.80	0.85	[0.43; 1.27]	6.5%
Kamel (2025)	22	2.64	3.96	22	3.00	1.59	−0.36	[−2.14; 1.42]	2.2%
El Ghamry (2019)	35	3.14	1.54	35	3.41	2.12	−0.27	[−1.14; 0.60]	4.7%
Gurkan (2020)	25	0.71	1.57	25	1.00	1.57	−0.29	[−1.16; 0.58]	4.7%
Singh (2023)	30	2.77	0.43	30	2.83	0.38	−0.06	[−0.27; 0.15]	7.1%
Muaziz (2025)	58	2.14	1.02	58	1.10	0.89	1.04	[0.69; 1.39]	6.7%
Eldemrdash (2019)	25	2.63	2.37	25	3.00	3.16	−0.37	[−1.92; 1.18]	2.6%
Kangle (2023)	25	1.52	0.87	25	1.32	0.85	0.20	[−0.28; 0.68]	6.3%
Nawar (2024)	50	2.50	0.70	50	3.60	0.60	−1.10	[−1.36; −0.84]	7.0%
Eskandr (2022)	20	2.90	0.69	20	2.45	0.79	0.45	[−0.01; 0.91]	6.3%
Yendrapati (2024)	50	5.10	0.33	50	6.30	1.45	−1.20	[−1.61; −0.79]	6.5%
**Random effects model**	**559**			**558**			**0.00**	**[−0.31; 0.32]**	**100.0%**

Heterogeneity: $I^2 = 91.0\%$, $\tau^2 = 0.3550$, $p < 0.0001$

Mean Difference

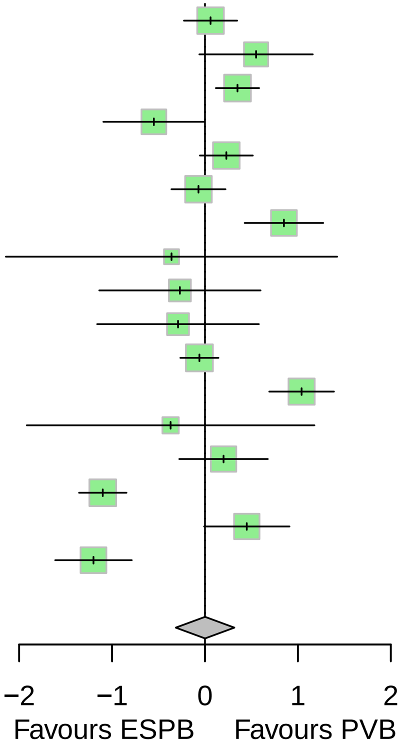

Pain at 12 h on Rest - Funnel Plot

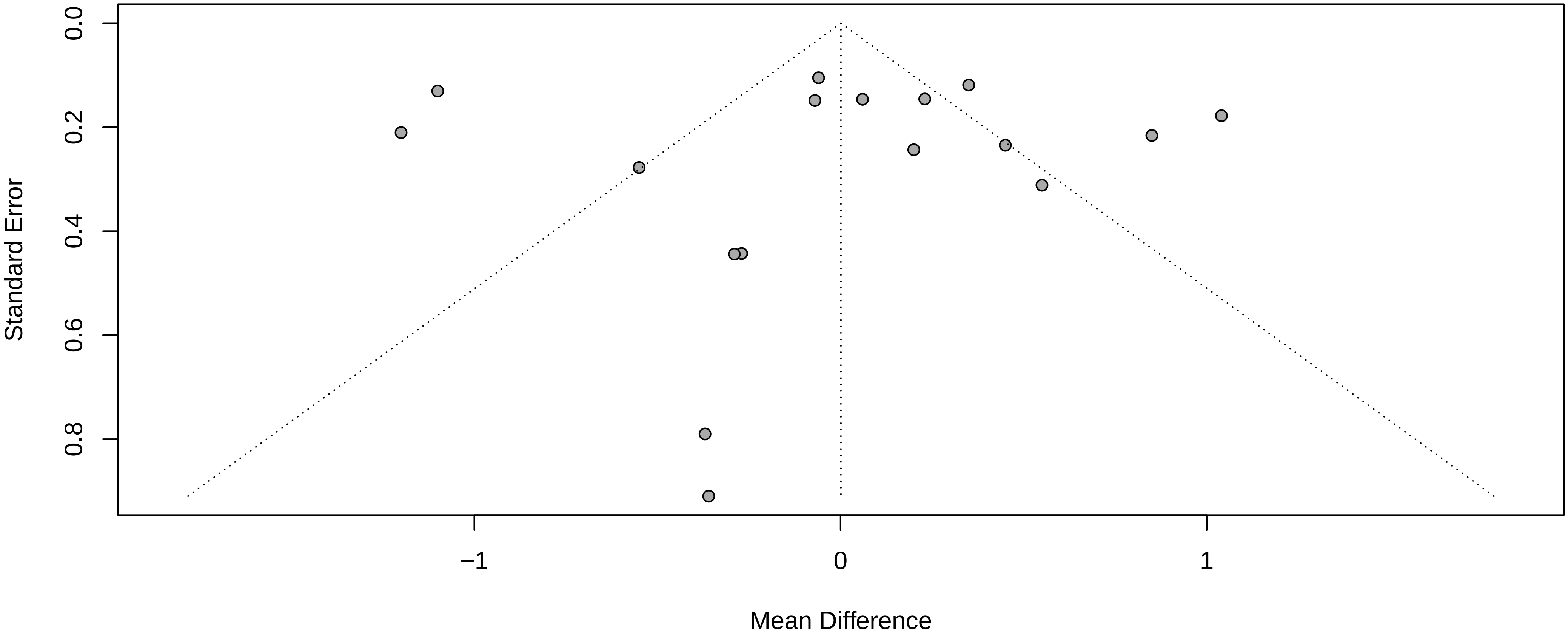

Pain at 12 h on Movement - Forest Plot

Study	ESPB Total	ESPB Mean	ESPB SD	PVB Total	PVB Mean	PVB SD	MD	95%−CI	Weight
Agarwal (2021)	40	2.64	0.72	40	2.70	0.58	−0.06	[−0.35; 0.23]	19.4%
Wittayapairoj (2022)	22	2.64	1.29	22	2.50	1.10	0.14	[−0.57; 0.85]	12.1%
Sivrikoz (2022)	42	1.65	2.30	41	0.71	1.54	0.94	[0.10; 1.78]	10.2%
Amr (2024)	35	4.23	0.52	35	3.43	0.96	0.80	[0.44; 1.16]	18.1%
Kamel (2025)	22	3.71	4.75	22	3.35	2.37	0.36	[−1.86; 2.58]	2.4%
Singh (2023)	30	2.90	0.31	30	3.00	0.01	−0.10	[−0.21; 0.01]	21.6%
Eskandr (2022)	20	3.93	1.00	20	3.27	0.34	0.66	[0.20; 1.12]	16.3%
**Random effects model**	**211**			**210**			**0.34**	**[−0.03; 0.71]**	**100.0%**

Heterogeneity: $I^2 = 82.8\%$, $\tau^2 = 0.1586$, $p < 0.0001$

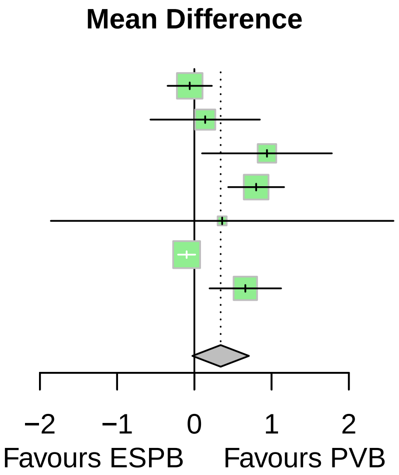

Pain at 12 h on Movement - Funnel Plot

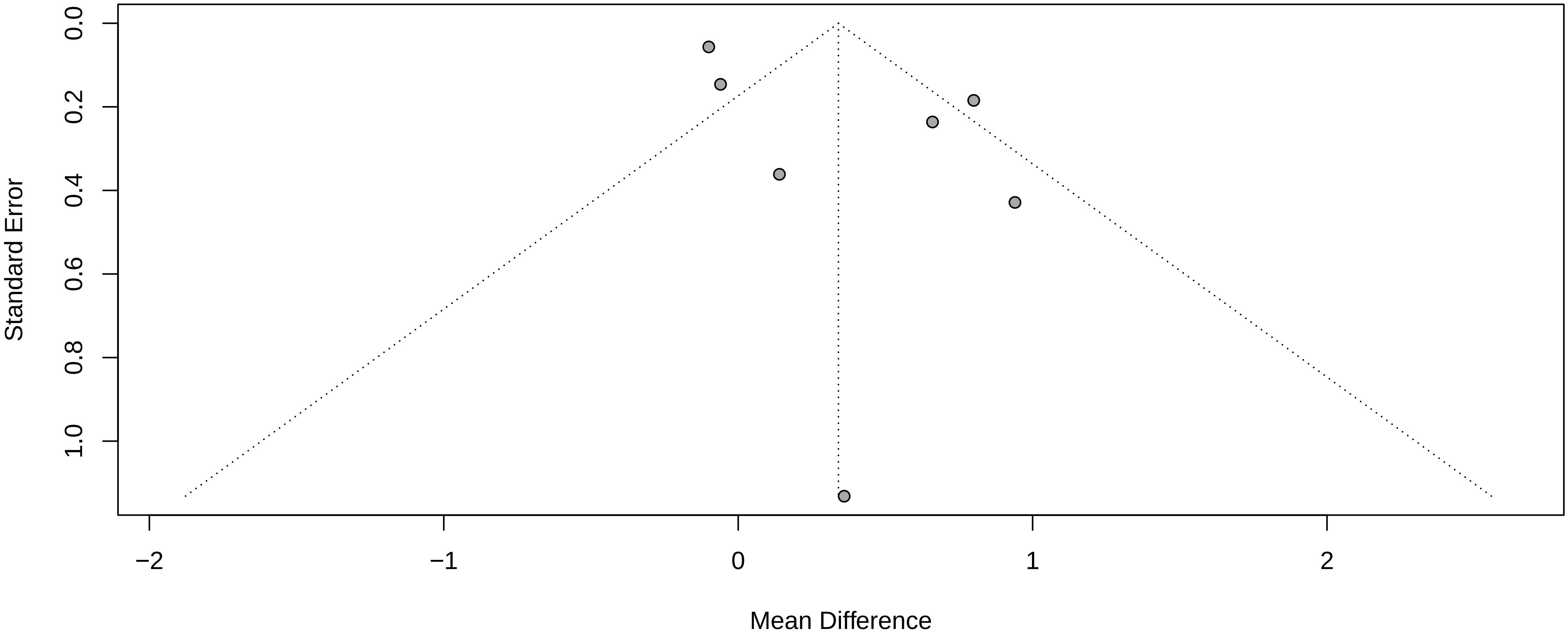

Pain at 24 h on Rest - Forest Plot

Study	ESPB Total	ESPB Mean	ESPB SD	PVB Total	PVB Mean	PVB SD	MD	95%−CI	Weight
Agarwal (2021)	40	1.70	0.58	40	1.64	0.72	0.06	[−0.23; 0.35]	8.0%
Wittayapairoj (2022)	22	0.68	0.95	22	0.50	0.86	0.18	[−0.36; 0.72]	6.1%
Sivrikoz (2022)	42	0.11	0.23	41	0.00	0.01	0.11	[0.04; 0.18]	9.1%
Elewa (2022)	30	4.64	0.78	30	5.18	1.17	−0.54	[−1.04; −0.04]	6.3%
Amr (2024)	35	3.34	0.86	35	2.97	0.79	0.37	[−0.02; 0.76]	7.2%
Jayakrishnan (2024)	30	0.97	0.61	30	0.80	0.61	0.17	[−0.14; 0.48]	7.8%
Sayed (2024)	20	4.30	0.53	20	4.00	1.07	0.30	[−0.22; 0.82]	6.2%
Kamel (2025)	22	2.28	3.17	22	3.00	1.59	−0.72	[−2.20; 0.76]	1.9%
El Ghamry (2019)	35	3.50	2.31	35	3.14	1.54	0.36	[−0.56; 1.28]	3.7%
Gurkan (2020)	25	0.35	0.78	25	0.71	1.57	−0.36	[−1.05; 0.33]	5.0%
Singh (2023)	30	3.60	0.77	30	3.70	0.75	−0.10	[−0.48; 0.28]	7.3%
Eldemrdash (2019)	25	3.00	3.16	25	3.00	3.16	0.00	[−1.75; 1.75]	1.4%
Kangle (2023)	25	1.20	0.57	25	1.16	0.62	0.04	[−0.29; 0.37]	7.7%
Nawar (2024)	50	2.80	0.80	50	3.20	0.40	−0.40	[−0.65; −0.15]	8.2%
Eskandr (2022)	20	3.35	0.67	20	2.41	0.74	0.94	[0.50; 1.38]	6.9%
Yendrapati (2024)	50	4.80	0.14	50	5.70	1.45	−0.90	[−1.30; −0.50]	7.1%
**Random effects model**	**501**			**500**			**−0.01**	**[−0.23; 0.22]**	**100.0%**

Heterogeneity: $I^2 = 77.1\%$, $\tau^2 = 0.1503$, $p < 0.0001$

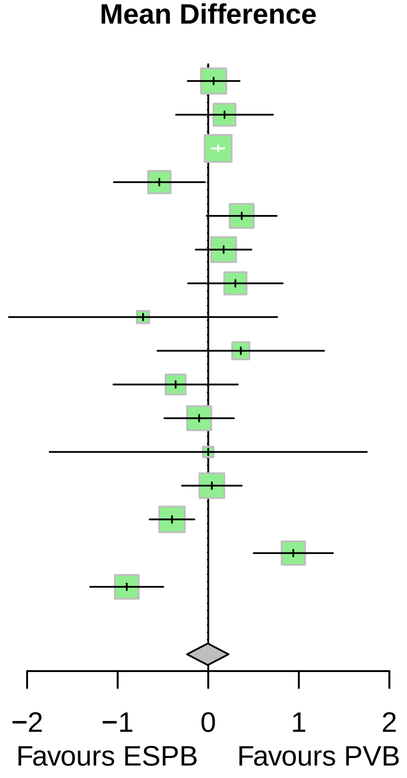

Pain at 24 h on Rest - Funnel Plot

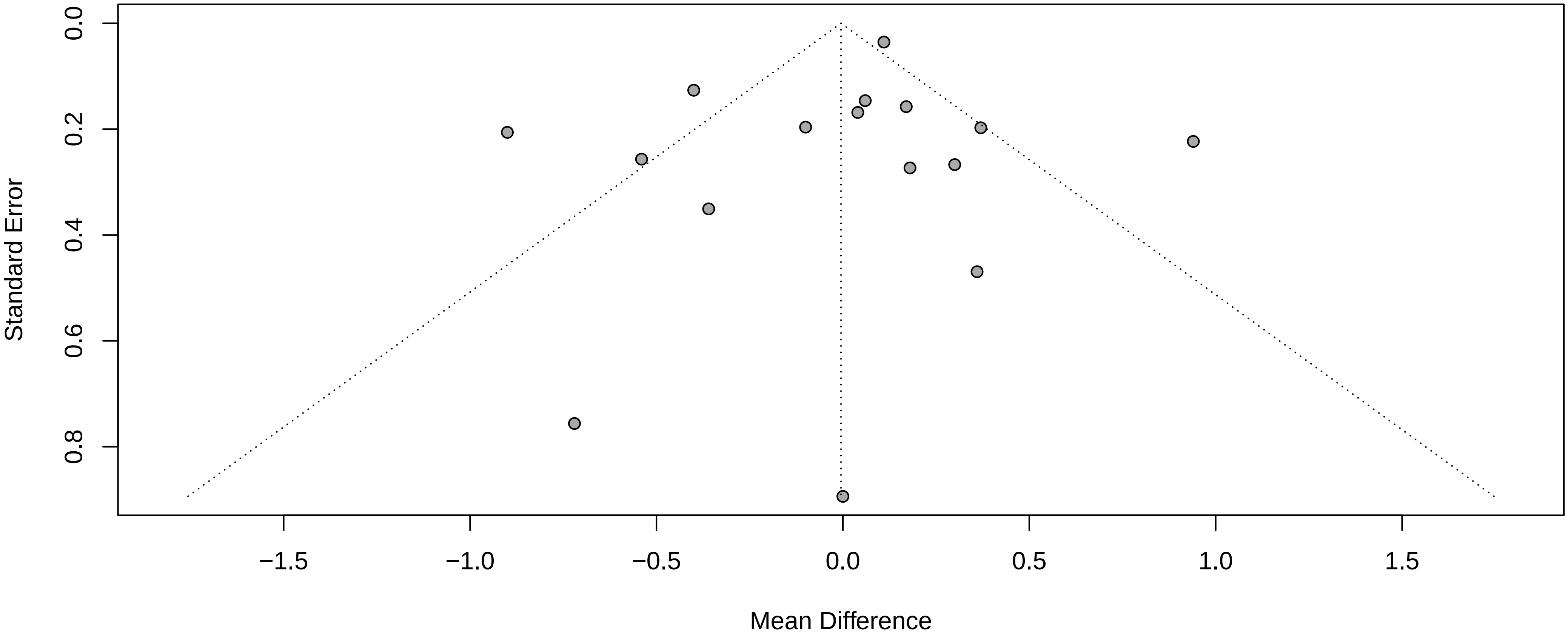

Pain at 24 h on Movement - Forest Plot

Study	ESPB Total	ESPB Mean	ESPB SD	PVB Total	PVB Mean	PVB SD	MD	95%−CI	Weight
Agarwal (2021)	40	2.64	0.72	40	2.70	0.58	−0.06	[−0.35; 0.23]	17.7%
Wittayapairoj (2022)	22	2.09	1.02	22	2.09	1.23	0.00	[−0.67; 0.67]	14.0%
Sivrikoz (2022)	42	1.06	2.30	41	0.00	0.01	1.06	[0.36; 1.76]	13.7%
Amr (2024)	35	3.86	0.59	35	3.00	0.83	0.86	[0.52; 1.20]	17.3%
Kamel (2025)	22	3.00	3.17	22	3.35	2.37	−0.35	[−2.00; 1.30]	6.1%
Singh (2023)	30	3.73	0.91	30	3.90	0.92	−0.17	[−0.63; 0.29]	16.1%
Eskandr (2022)	20	4.61	0.99	20	3.29	0.78	1.32	[0.77; 1.87]	15.2%
**Random effects model**	**211**			**210**			**0.44**	**[−0.06; 0.93]**	**100.0%**

Heterogeneity: $I^2 = 84.6\%$, $\tau^2 = 0.3389$, $p < 0.0001$

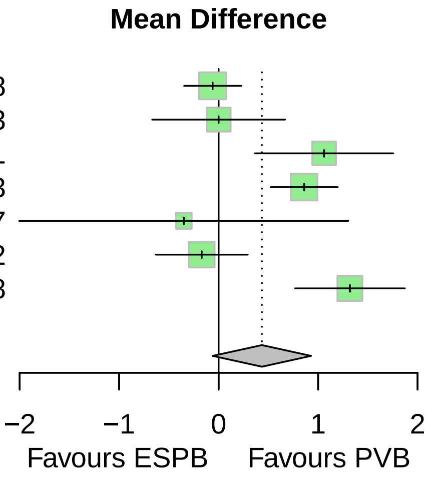

Pain at 24 h on Movement - Funnel Plot

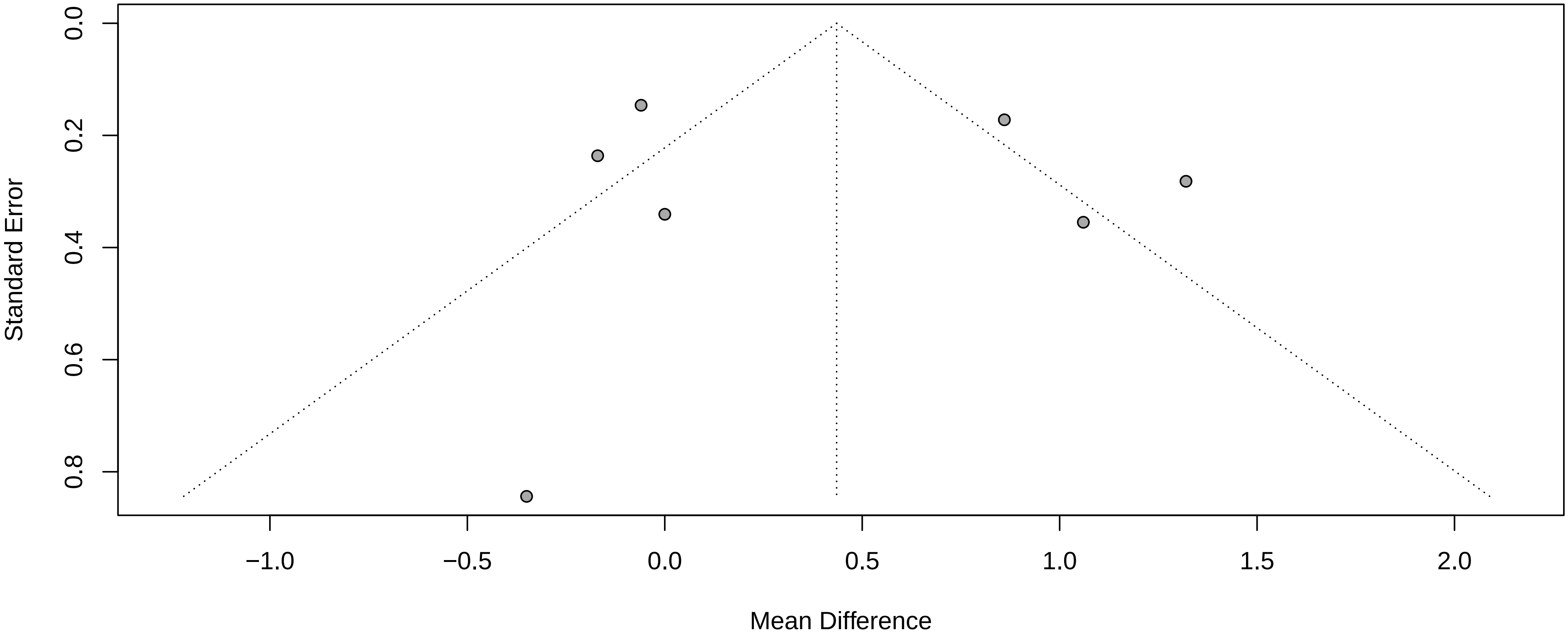

PONV - Forest Plot

Study	ESPB Events	ESPB Total	PVB Events	PVB Total	OR	95%−CI	Weight
Agarwal (2021)	2	40	3	40	0.65	[0.10; 4.11]	5.4%
Wittayapairoj (2022)	3	22	0	22	8.08	[0.39; 166.27]	2.0%
Sivrikoz (2022)	2	42	1	41	2.00	[0.17; 22.95]	3.1%
Elewa (2022)	3	30	3	30	1.00	[0.19; 5.40]	6.4%
Amr (2024)	7	35	6	35	1.21	[0.36; 4.04]	12.5%
Abdelfatah (2022)	1	23	2	23	0.48	[0.04; 5.66]	3.0%
El Ghamry (2019)	10	35	12	35	0.77	[0.28; 2.11]	17.8%
Gurkan (2020)	7	25	2	25	4.47	[0.83; 24.19]	6.4%
Singh (2023)	5	30	4	30	1.30	[0.31; 5.40]	9.0%
Varshney (2020)	8	30	10	30	0.73	[0.24; 2.21]	14.8%
Anandhkumar (2022)	3	27	7	27	0.36	[0.08; 1.56]	8.4%
Eskandr (2022)	4	20	4	20	1.00	[0.21; 4.71]	7.6%
Yendrapati (2024)	1	50	4	50	0.23	[0.03; 2.18]	3.7%
**Random effects model**		**409**		**408**	**0.93**	**[0.61; 1.42]**	**100.0%**

Heterogeneity: $I^2 = 0.0\%$, $\tau^2 < 0.0001$, $p = 0.6241$

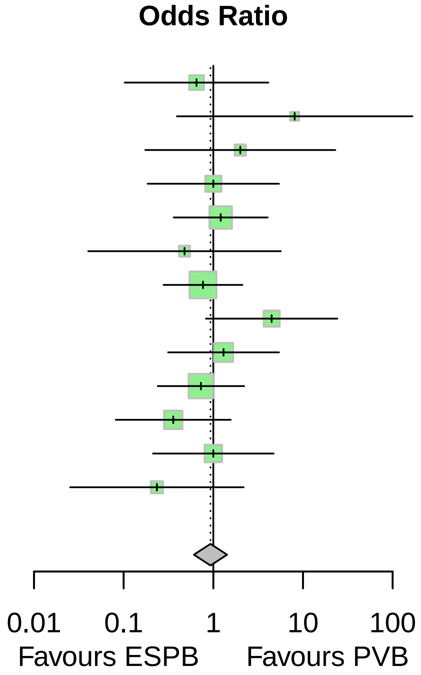

PONV - Funnel Plot

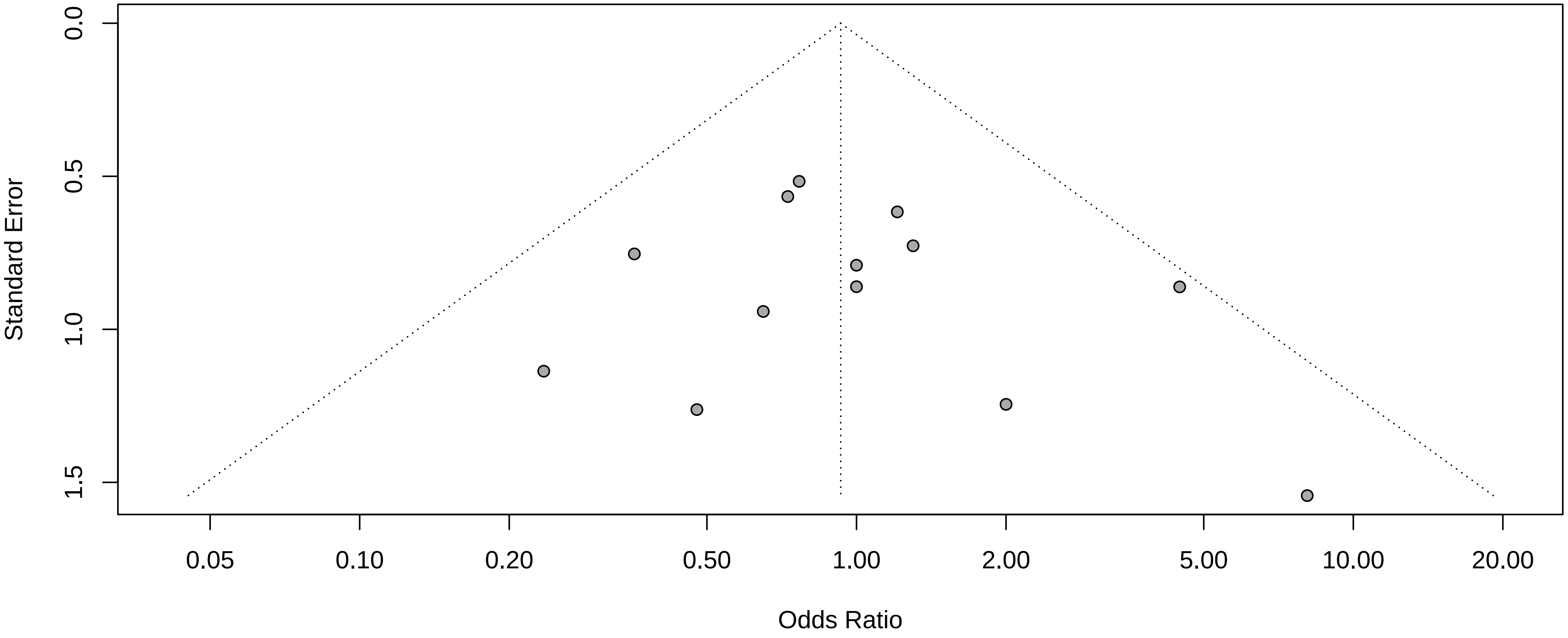

Rescue Analgesia - Forest Plot

Study	ESPB Events	ESPB Total	PVB Events	PVB Total	OR	95%−CI	Weight
Wittayapairoj (2022)	22	22	14	22	26.38	[1.41; 492.81]	4.1%
Sivrikoz (2022)	2	42	1	41	2.00	[0.17; 22.95]	5.9%
Kamel (2025)	10	22	8	22	1.46	[0.44; 4.88]	24.0%
Abdelfatah (2022)	6	23	7	23	0.81	[0.22; 2.92]	21.1%
Singh (2023)	19	30	17	30	1.32	[0.47; 3.72]	32.6%
Kangle (2023)	2	25	7	25	0.22	[0.04; 1.21]	12.3%
**Random effects model**		**164**		**163**	**1.13**	**[0.63; 2.05]**	**100.0%**

Heterogeneity: $I^2 = 42.7\%$, $\tau^2 < 0.0001$, $p = 0.1208$

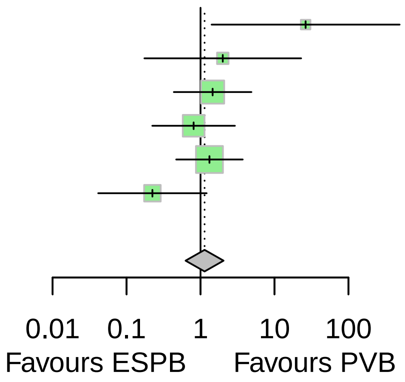

Rescue Analgesia - Funnel Plot

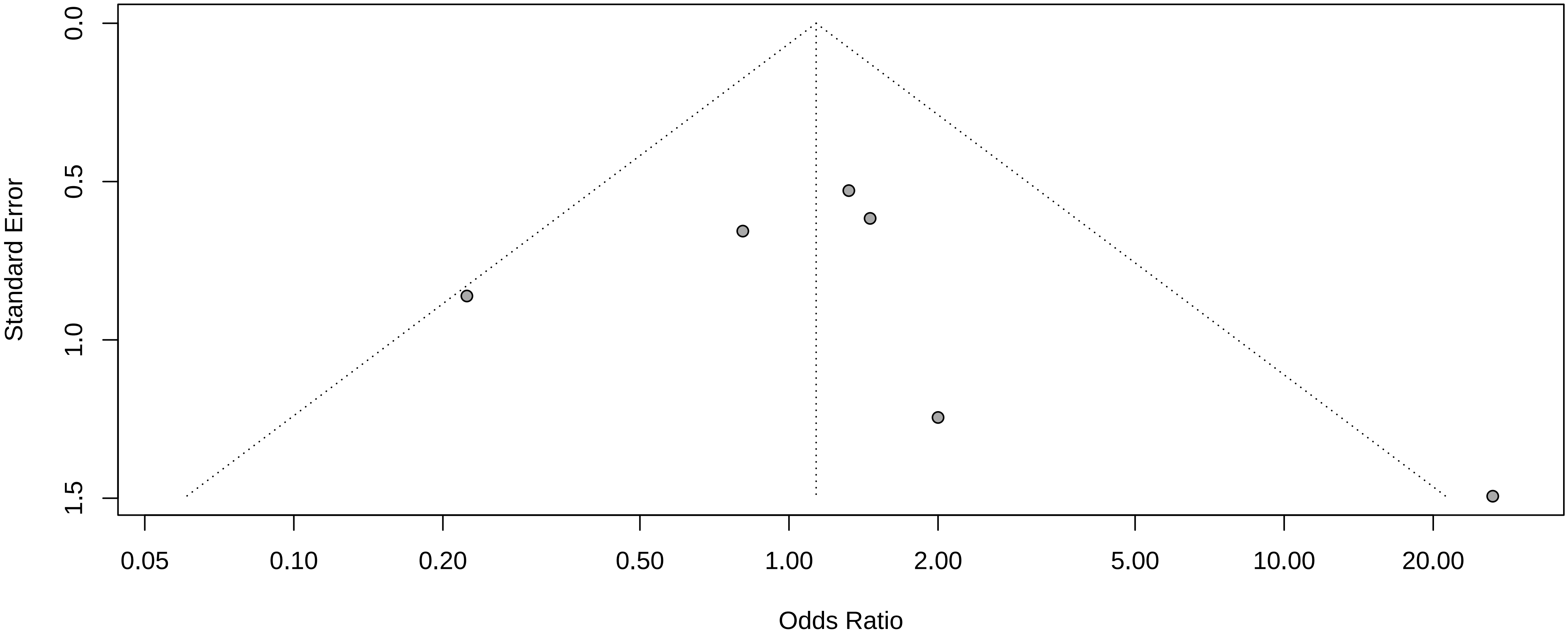

Sensitivity analysis for primary outcome

Study	ESPB Total	ESPB Mean	ESPB SD	PVB Total	PVB Mean	PVB SD	MD	95%-CI	Weight
Amr (2024)	35	8.17	1.70	35	5.70	1.90	2.47	[1.63; 3.31]	8.3%
Attri (2024)	30	5.69	3.45	30	6.32	3.21	-0.63	[-2.32; 1.06]	6.5%
Elewa (2022)	30	4.90	1.20	30	5.80	1.30	-0.90	[-1.53; -0.27]	8.6%
El Ghamry (2019)	35	26.70	2.10	35	27.30	2.90	-0.60	[-1.79; 0.59]	7.6%
Eskandr (2022)	20	5.80	1.51	20	6.00	1.86	-0.20	[-1.25; 0.85]	7.9%
Gürkan (2020)	25	5.36	4.72	25	5.28	4.72	0.08	[-2.54; 2.70]	4.6%
Jayakrishnan (2024)	30	6.30	2.53	30	5.13	3.63	1.17	[-0.41; 2.75]	6.7%
Kamel (2025)	22	7.08	2.38	22	6.00	4.75	1.08	[-1.14; 3.30]	5.3%
Moustafa (2020)	45	6.17	2.08	45	6.22	2.09	-0.05	[-0.91; 0.81]	8.2%
Nawar (2024)	50	5.30	1.10	50	6.10	1.00	-0.80	[-1.21; -0.39]	8.9%
Sayed (2024)	20	8.70	2.18	20	8.00	2.34	0.70	[-0.70; 2.10]	7.1%
Singh (2023)	30	3.16	2.45	30	2.83	2.52	0.33	[-0.93; 1.59]	7.4%
Sivrikoz (2022)	42	21.00	3.10	41	19.20	2.90	1.80	[0.51; 3.09]	7.4%
Wittayapairoj (2022)	22	8.60	3.80	22	3.50	3.30	5.10	[3.00; 7.20]	5.6%
**Random effects model**	**436**			**435**			**0.58**	**[-0.21; 1.37]**	**100.0%**

Heterogeneity: $I^2 = 85.7\%$, $\tau^2 = 1.7799$, $p < 0.0001$

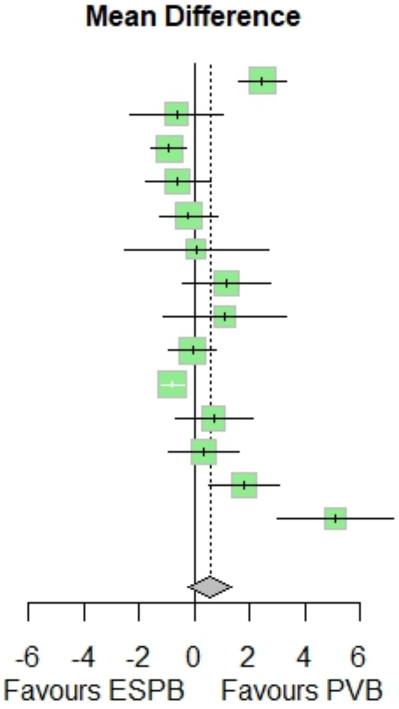

Trial sequential analysis

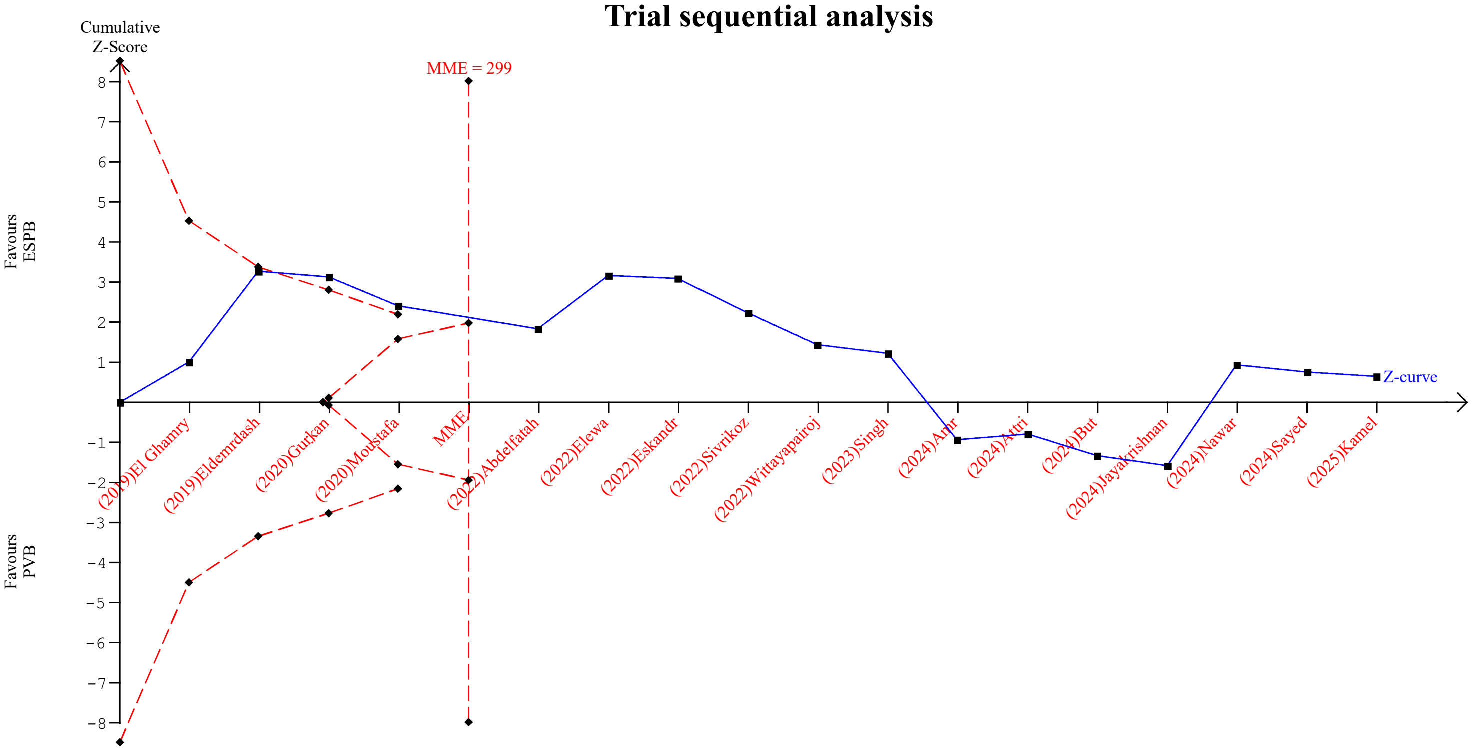

Certainty assessment						
Participants (studies) Follow-up	Risk of bias	Inconsistency	Indirectness	Imprecision	Publication bias	Overall certainty of evidence
**24-hour opioid consumption**						
1021 (17 RCTs)	serious[a]	very serious[b]	not serious	not serious	serious[c]	⊕○○○ Very Low
**Pain at rest – 0 postoperative hours**						
475 (8 RCTs)	serious[a]	serious[d]	not serious	serious[e]	none	⊕⊕○○ Low
**Pain at rest – 6 postoperative hours**						
475 (8 RCTs)	serious[a]	serious[d]	not serious	serious[e]	none	⊕⊕○○ Low
**Pain at rest – 24 postoperative hours**						
421 (7 RCTs)	serious[a]	serious[d]	not serious	serious[e]	none	⊕⊕○○ Low
**Pain on movement – 24 postoperative hours**						
421 (7 RCTs)	serious[a]	serious[d]	not serious	serious[e]	none	⊕⊕○○ Low
**Need for rescue analgesia**						
327 (6 RCTs)	serious[a]	serious[d]	not serious	serious[e]	none	⊕⊕○○ Low
**Postoperative nausea and vomiting**						
817 (13 RCTs)	serious[a]	not serious	not serious	not serious	none	⊕⊕⊕○ Modarete

Explanations

[a]Several included trials were at high risk of bias and many had some concerns, mainly related to randomization and blinding.

[b]Very substantial heterogeneity was present ($I^2 > 75\%$), with important between-study variability not fully explained.

[c]Funnel plot assessment and a significant Egger's test suggested potential publication bias/small-study effects.

[d]Although a statistically significant difference was observed, the magnitude of effect was small and did not reach a clinically meaningful threshold, with confidence intervals indicating limited clinical relevance.

[e]Confidence intervals crossed the line of no effect and the estimated effect size was small.

Meta-Regression

Variable		Estimate (95%CI)	p value	R2
**Country**	India	0.37 (-1.28;2.03)	0.658	57.11%
	Poland	5.07 (1.85;8.27)	0.002	
	Thailand	5.17 (2.11;8.21)	< 0.001	
	Turkiye	1.33 (-0.15;2.83)	0.153	
**Age > 65**	Yes	1.96 (0.51;3.40)	0.008	32.57%
**LA**	Bupivacaine	2.53 (-0.27;5.32)	0.077	40.21%
	Levobupivacaine	7.10 (2.88;11.31)	< 0.001	
	Ropivacaine	2.33 (-1.53;6.19)	0.237	
**Adjuvants**	Yes	-1.70 (-3.81;0.42)	0.116	8.95%
**Volume**	mL	-0.08 (-2.65;7.30)	0.496	0.0%
**Concentration**	%	-1.00 (-2.99;1.00)	0.329	0.27%

Abbrevations: LA: Local Anesthetic.

Subgroup analysis and sensitivity analysis

**Outcome**	**N**	**ESPB**	**PVB**	**MD (95% CI)**	**I^2 (%)**	**p value**
**Risk of bias (Sensitivity analysis)**						
**Low**	14	436	435	0.58 (-0.21; 1.37)	85.7	0.151
**Type of surgery**						
**Outcome**	**N**	**ESPB**	**PVB**	**MD (95% CI)**	**I^2 (%)**	
**Breast conservative surgery**	1	25	25	-0.20 (-1.25;0.85)	NA	
**Mastectomy**	20	658	663	0.80 (-0.19;1.82)	89.2	
**Both**	6	321	323	-0.42 (-1.84;1.00)	0	
**Surgery involves the axillary region**						
**Yes**	16	631	638	0.66 (-0.38;1.70)	89.6	
**No**	1	20	20	-0.20 (-1.25;-0.85)	N/A	
**Both/No information**	10	353	353	-0.76 (-1.28;0.85)	83	

Abbrevations: CI: Confidence Interval, ESPB: Erector Spinae Plane Block, N/A: Not Applicable, N: Number of studies, MD: Mean Difference, PVB: Paravertebral Block.
